# Antiproliferative Activity and Impact on Human Gut Microbiota of New *O*-Alkyl Derivatives of Naringenin and Their Oximes

**DOI:** 10.3390/ijms24129856

**Published:** 2023-06-07

**Authors:** Joanna Kozłowska, Anna Duda-Madej, Dagmara Baczyńska

**Affiliations:** 1 Department of Food Chemistry and Biocatalysis, Faculty of Biotechnology and Food Science, Wrocław University of Environmental and Life Sciences, C.K. Norwida 25, 50-375 Wrocław, Poland; 2 Department of Microbiology, Faculty of Medicine, Wroclaw Medical University, Chałubińskiego 4, 50-368 Wrocław, Poland; anna.duda-madej@umw.edu.pl; 3 Department of Molecular and Cellular Biology, Faculty of Pharmacy with Division of Laboratory Diagnostics, Wroclaw Medical University, Borowska 211A, 50-556 Wrocław, Poland; dagmara.baczynska@umw.edu.pl

**Keywords:** anticancer activity, colorectal cancer, *Enterococcus faecalis*, *Escherichia coli*, HT-29, human microbiota, naringenin, *O*-alkyl derivatives, oximes, *Staphylococcus aureus*

## Abstract

Naringenin is a 5,7,4′-trihydroxyflavanone naturally occurring mainly in citrus fruits, characterized by a wide spectrum of biological activity. Chemical modifications based on alkylation and oximation in most cases increase its bioactivity. The aim of our research was to evaluate the antiproliferative activity and influence on selected representatives of the human gut microbiota of new synthesized *O*-alkyl derivatives (**A1**–**A10**) and their oximes (**B1**–**B10**), which contain hexyl, heptyl, octyl, nonyl and undecyl chains attached to the C-7 or to both the C-7 and C-4′ positions in naringenin. To the best of our knowledge, compounds **A3**, **A4**, **A6**, **A8**–**A10** and **B3**–**B10** have not been described in the scientific literature previously. The anticancer activity was tested on human colon cancer cell line HT-29 and mouse embryo fibroblasts 3T3-L1 using the sulforhodamine B (SRB) and 3-(4,5-dimethylthiazol-2-yl)-2,5-diphenyl-2H-tetrazolium bromide (MTT) assays. We also determined the impacts of all compounds on the growth of Gram-positive and Gram-negative bacterial strains, such as *Staphylococcus aureus*, *Enterococcus faecalis* and *Escherichia coli*. The antimicrobial activity was expressed in terms of minimal inhibitory concentrations (MIC) and minimal bactericidal concentrations (MBC) values. For 7,4′-di-*O*-hexylnaringenin (**A2**), 7-*O*-undecylnaringenin (**A9**) and their oximes (**B2**, **B9**), which were safe for microbiota (MIC > 512 µg/mL) and almost all characterized by high cytotoxicity against the HT-29 cell line (**A2**: IC_50_ > 100 µg/mL; **A9**: IC_50_ = 17.85 ± 0.65 µg/mL; **B2**: IC_50_ = 49.76 ± 1.63 µg/mL; **B9**: IC_50_ = 11.42 ± 1.17 µg/mL), apoptosis assays were performed to elucidate their mechanisms of action. Based on our results, new compound **B9** induced an apoptotic process via caspase 3/7 activation, which proved its potential as an anticancer agent.

## 1. Introduction

Recent statistical data show that cancer represents one of the most important global public health problems and the second leading cause of death in the United States [1]. Among all types, colorectal cancer ranks third as the most often diagnosed cancer, and it accounted for 9.4% of deaths in 2020 around the world [2,3]. It is estimated that by 2035, the number of new cases could be doubled [3]. Among the risk factors for colorectal cancer are (1) a family and personal medical history, such as genetic predispositions; (2) inflammatory bowel disease (IBD) or colon polyps; (3) lifestyle, including food habits, cigarette smoking, alcohol consumption and physical inactivity; (4) and others, e.g., gut microbiota, age, gender and socioeconomic factors [4].

Nowadays, there are many therapeutic options with various routes of drug administration, and the most popular are intravenous, intraarterial, intraperitoneal and intrathecal forms [5,6]. However, they are uncomfortable for patients and also may result in side effects, e.g., gastrointestinal problems or anaphylactic reactions. In addition, with long-term use, they affect the circulatory, urinary and nervous systems, as well as the lower respiratory tract. The oral form is the most convenient for the patient, but, due to the small number of this type of drugs, it is the least commonly used route in treatment [7]. Therefore, to prevent the development and influence the inhibition of the early stage of the disease, our dietary patterns play an important role. The diet should be balanced and abundant not only in vitamins—especially vitamins B and E, amino acids and their derivatives, e.g., acetyl-L-carnitine, fatty acids, probiotics and prebiotics—and medicinal plants, but also in vegetables, fruits, spices and plant extracts, which are major sources of valuable bioactive secondary metabolites, such as terpenoids, alkaloids and flavonoids, characterized by anticancer properties [8,9,10,11]. It should be mentioned that bioactive compounds play a crucial role in the prevention of cancer by affecting the regulation of the gut microbiota [12]. However, they could also interact with drugs used in chemotherapy and cause many undesirable effects; therefore, their selection requires continuous research.

Naringenin is a 5,7,4′-trihydroxyflavanone commonly and abundantly found in many citrus fruits, such as grapefruit, sweet orange, pomelo, mandarin and bergamot, but also in ashwagandha, cocoa, cherries or plant-derived products, such as juices [13]. It is known that naringenin provides health-promoting properties, such as antioxidant [14], antibacterial, antifungal [15], anti-inflammatory, antiviral [16]—including activity against COVID-19 [17]—and anticancer activity [18].

Furthermore, the search for new compounds of natural origin or chemical analogues of natural substances has attracted significant interest among scientists. Our previous research showed that modifications of naringenin by alkylation and oximation strongly increased its biological properties, such as antioxidant, antibacterial and anticancer, and also exhibited an effect on the probing behavior of *Myzus persicae* (Sulz.) [19,20,21,22]. The introduction of an alkyl chain into the naringenin molecule increases its lipophilicity, which enables it to penetrate into cells and enhances its anticancer activity [23,24]. Oximes are one of the most commonly used derivatives containing a nitrogen atom in medicinal chemistry, and they can be easily obtained by chemical synthesis from carbonyl compounds. In addition, they are known as the protective groups of carbonyl compounds and are also involved as intermediates in the Beckmann rearrangement in the synthesis of β-lactams [25]. Recent studies found that *O*-alkyl derivatives containing six to twelve carbon atoms in the chain attached to the C-7 position in naringenin exhibited strong cytotoxic effects against melanoma (B16–F10) cells, with an IC_50_ value in range of 22.6–25.4 µM [26]. However, in the same studies, 7-*O*-butylnaringenin possessed weaker activity, and, interestingly, an ether derivative with sixteen carbon chains lost its activity [26]. In the case of human colorectal carcinoma RKO cells, Lee and coworkers observed that the most effective were 7-*O*-benzyl- and 7-*O*-(*m*-metoxybenzyl)-substituted naringenin. Both induced apoptosis via the activation of caspase, intracellular reactive oxygen species (ROS) production and sustained extracellular signal-regulated kinase (ERK) activation [27]. Moreover, Zhang et al. showed that 7,4′-di-*O*-methylnaringenin inhibited the proliferation of the HCT-116 colon cancer cell line and induced apoptosis through the activation of p53 protein expression [28]. Moreover, the introduction an oxime moiety to naringenin resulted in a two-times greater cytotoxic effect against human breast (MCF-7) and human colon cancer cells (HT-29) [29]. Additionally, oxime ether derivatives showed cytotoxic activity against human leukemia (HL-60), gynecological cancer cell lines (HeLa, SiHa) and breast cancers (MCF-7, MDA-MB-231). As in the case of the above-mentioned derivatives, their mechanism of action is related to the induction of apoptosis by the activation of caspase 3 [30]. Interestingly, Ferreira et al. showed that 7-*O*- and/or 7,4′-di-*O*-alkylation with the simultaneous incorporation of nitrogen atoms into the C-4 position in naringenin significantly improved its anticancer activity [31].

In this paper, we present the antiproliferative activity of new *O*-alkyl derivatives and their oximes against human colon cancer cell line HT-29, and we compare it with the activity against mouse fibroblast cell line 3T3-L1. Considering the protection of the intestinal microbiome, we determine the effects of the synthesized compounds on selected representatives of the human gut microbiota, such as *Staphylococcus aureus*, *Enterococcus faecalis* and *Escherichia coli*, physiologically inhabiting the terminal gastrointestinal tract. Based on the results, we select compounds to evaluate their mechanisms of action as potential anticancer agents that would not have a negative impact on the human microbiota.

## 2. Results and Discussion

### 2.1. Chemistry

**Scheme 1:**
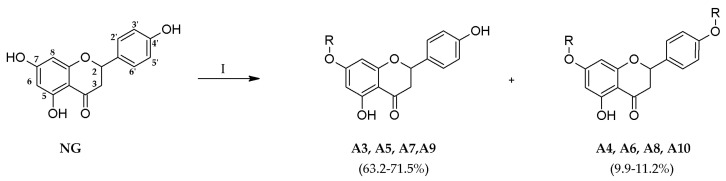
Synthesis of 7-*O*-alkyl (**A3**, **A5**, **A7**, **A9**) and 7,4′-di-*O*-alkyl (**A4**, **A6**, **A8**, **A10**) derivatives of naringenin; 5,7,4′-tri-*O*-alkyl derivatives were not observed. (I): alkyl iodide, anhydrous K_2_CO_3_, DMF, r.t., 17–18 h; **NG**—naringenin, **A3** and **A4**: R = (CH_2_)_6_-CH_3_, **A5** and **A6**: R = (CH_2_)_7_-CH_3_, **A7** and **A8**: R = (CH_2_)_8_-CH_3_, **A9** and **A10**: R = (CH_2_)_10_-CH_3_.

In our previous studies, we described the simultaneous preparation of 7-*O*-hexylnaringenin (**A1**) and 7,4′-di-*O*-hexylnaringenin (**A2**), which were further modified to 7-*O*-hexylnaringenin oxime (**B1**) and 7,4′-*O*-hexylnaringenin oxime (**B2**), respectively [32]. In this paper, we focus on extending this group of compounds. In the first stage of our research, we synthesized new *O*-alkyl derivatives of naringenin containing heptyl, octyl, nonyl and undecyl chain(s) attached to the C-7 or C-7 and C-4′ position(s) in naringenin (**NG**). Ether derivatives (**A3**–**A10**) were obtained in the reaction of naringenin with an appropriate amount of alkyl iodide in the presence of anhydrous potassium carbonate conducted in an organic solvent—*N*,*N*-dimethylformamide (DMF) (Scheme 1). In our previous work, the use of DMF resulted in obtaining not only mono- and di- but also tri-*O*-alkyl derivatives [20,21]. However, ether derivatives containing three carbon chains exhibit low solubility and generally low antibacterial and anticancer activity; thus, in our research, we focused only on 7-*O*- and 7,4′-di-*O*-alkyl derivatives. In this study, no 5,7,4′-tri-*O*-alkyl derivatives were obtained under the reaction conditions. Moreover, we observed that the use of DMF instead of anhydrous acetone resulted in a shorter reaction time [21].

To our knowledge, among the synthesized *O*-alkyl derivatives of naringenin, compounds **A3**, **A4**, **A6**, **A8**, **A9** and **A10** are new and have never been described in the literature. A recent report presented by Albuquerque de Oliveira Mendes et al. showed that the addition of potassium *tert*-butoxide and alkyl halide (chloride or bromide) to naringenin dissolved in DMF resulted in obtaining 7-*O*-octyl- and 7-*O*-nonylnaringenin with yields of 23% and 30%, respectively [26]. According to our methodology, we obtained, in a shortened time, the same products, but with 2–3 times higher yields (Table 1).

**Scheme 2:**
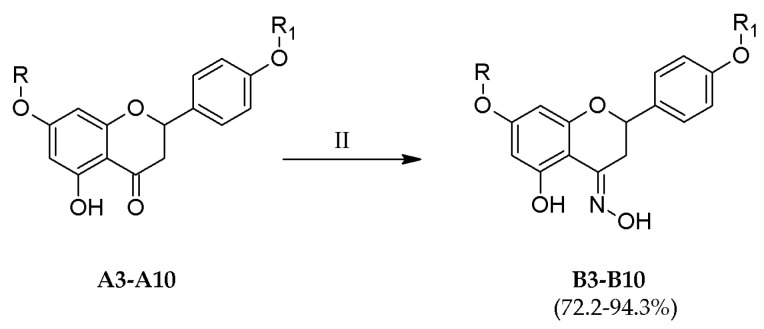
Synthesis of oximes (**B3**–**B10**). (II): NH_2_OH·HCl, CH_3_COONa, EtOH, 45 °C, 17–27 h; **A3** and **B3**: R = (CH_2_)_6_-CH_3_, R_1_ = H, **A4** and **B4**: R = R_1_ = (CH_2_)_6_-CH_3_, **A5** and **B5**: R = (CH_2_)_7_-CH_3_, R_1_ = H, **A6** and **B6**: R = R_1_ = (CH_2_)_7_-CH_3_, **A7** and **B7**: R = (CH_2_)_8_-CH_3_, R_1_ = H, **A8** and **B8**: R = R_1_ = (CH_2_)_8_-CH_3_, **A9** and **B9**: R = (CH_2_)_10_-CH_3_, R_1_ = H, **A10** and **B10**: R = R_1_ = (CH_2_)_10_-CH_3_.

In the next step, we synthesized oximes of *O*-alkyl derivatives of naringenin **B3**–**B10** in a reaction with hydroxylamine hydrochloride and sodium acetate conducted in anhydrous ethanol (Scheme 2). All compounds **B3**–**B10** are new and were obtained in a maximum reaction time of 27 h, with isolated yields of up to 94%.

The structures of the obtained compounds were confirmed using ^1^H, ^13^C, ^1^H-^1^H (COSY) and ^1^H-^13^C (HSQC) NMR spectra and high-resolution mass spectrometry (HRMS). Analysis of the ^1^H NMR spectra confirmed the attachment of the alkyl chain to the C-7 position (**A3**, **A5**, **A7**, **A9**) and to the C-7 and C-4′ positions (**A4**, **A6**, **A8**, **A10**). In the case of mono- and disubstituted naringenin derivatives, a singlet from OH-5 was observed in the range of 12.02–12.01 ppm, and, for 7-*O*-alkyl naringenin, a broad singlet in the range of 5.38–5.03 ppm from OH-4′ was identified. Furthermore, characteristic of the flavanone skeleton doublets of doublets from H-2, H-3a and H-3b were detected for each derivative. Analysis of the ^13^C NMR spectra confirmed the presence of the carbonyl group as a signal at 196.17–196.07 ppm.

In the case of oximes, analysis of the ^1^H NMR spectrum proved the presence of an oxime moiety. With the exception of singlets from OH-5 or both OH-5 and OH-4′, a characteristic singlet from NOH at 11.01–11.00 ppm was observed. Moreover, a downshifted signal from C=NOH on the ^13^C NMR spectrum at 154.88–153.88 ppm was observed.

The isolated yields of all compounds and their melting points are shown in Table 1.

### 2.2. Influence of O-Alkyl Derivatives of Naringenin and Their Oximes on Human Microbiota

To evaluate the antimicrobial potential of the synthesized compounds (**A1**–**A10**, **B1**–**B10**), the minimal inhibitory concentrations (MIC) and minimal bactericidal concentrations (MBC) were determined against three representatives of the human microbiota, *S. aureus* ATCC 25923, *E. faecalis* ATCC 29212 and *E. coli* K12. All tested *O*-alkyl derivatives of naringenin (**A1**–**A10**) and their oximes (**B1**–**B10**) had no effect on the growth of *E. coli*, the most numerous Gram-negative bacterium in the intestinal microbiome (MIC and MBC values > 512 µg/mL). With regard to the activity of naringenin (**NG**) and its oxime (**NGOX**), the MIC value was 512 µg/mL and the MBC was above the tested concentration range (>512 µg/mL). Significantly different results were observed for almost all compounds for both tested Gram-positive strains (Table 2).

In the case of *S. aureus*, the complete inhibition of growth was observed in the presence of *O*-alkyl derivatives containing hexyl, heptyl and nonyl groups attached to the C-7 position in naringenin (**A1**, **A3** and **A7**), with the MIC value of 16 µg/mL. However, 7-*O*-octylnaringenin (**A5**) showed the strongest activity against this bacterial strain (MIC = 8 µ g/mL). Among monosubstituted analogues of naringenin, 7-*O*-undecylnaringenin (**A9**) had no influence on the growth of *S. aureus* (MIC > 512 µg/mL). Interestingly, di-*O*-alkyl derivatives of naringenin containing six, seven and eight carbon chains (**A2**, **A4** and **A6**) also had no effect on this Gram-positive strain (MIC > 512 µg/mL). Surprising results were observed for compounds with longer alkyl chains—**A8** (with two *O*-nonyl groups) and **A10** (with two *O*-undecyl groups)—which exhibited weak activity, with the MIC values of 512 µg/mL and 256 µg/mL, respectively. These results showed that the elongation of the carbonyl chains, which were attached to the C-7 and C-4′ positions, had a superior impact on the multiplication of *S. aureus* in comparison to di-*O*-hexyl, di-*O*-heptyl and di-*O*-octyl naringenin ethers.

For oximes, the strongest activity against *S. aureus* was observed for 7-*O*-hexylnaringenin oxime (**B1**), with the MIC value of 8 µg/mL. Excluding compound **B7** (with the MIC = 128 µg/mL), we observed that the potency of oximes **B3**, **B5** and **B9** decreased with the lengthening of the carbon chain and led to the complete inhibition of growth (MIC for **B9** > 512 µg/mL). Furthermore, none of di-*O*-alkyl derivatives (**B2**, **B4**, **B6**, **B8** and **B10**) showed activity against this representative of the intestinal microflora. Summarizing, we observed that the introduction of an oxime moiety resulted in 4–32 times weaker activity against *S. aureus*. Moreover, naringenin oxime, in comparison to naringenin, had a two-fold lower impact on this bacterial strain.

In the case of *E. faecalis*, both 7-*O*-alkyl and 7-di-*O*-alkyl ethers (**A1**–**A7** and **A10**) exhibited strong activity, with MIC values in range of 16–64 µg/mL. The weakest impact on the multiplication of this bacterial strain was observed in the presence of 7,4′-di-*O*-nonylnaringenin (**A8**) and 7-*O*-undecylnaringenin (**A9**) (MIC = 512 µg/mL). Excluding compounds **B1** and **B5** (MIC values on the same level as for **A1** and **A5**, MIC = 16 µg/mL), for oximes of monosubstituted derivatives **B3**, **B7** and **B9**, the incorporation of the NOH group resulted in a two-times lower impact on the growth of *E. faecalis*. Similarly as in the case of *S. aureus*, 7,4′-di-*O*-alkyl oximes (**B2**, **B4**, **B6**, **B8** and **B10**) were safe against this representative of the intestinal microflora (MIC > 512 µg/mL). Interestingly, the oxime group instead of the carbonyl moiety in naringenin resulted in slightly stronger activity against *E. faecalis* (Table 2).

The MBC values of all *O*-alkyl derivatives of naringenin were above 512 µg/mL against all tested strains. However, the introduction of the oxime group increased the killing potential for compounds **B1** (32 µg/mL) and **B3** (128 µg/mL) for *S. aureus*, as well as **B1** (256 µg/mL), **B3** (512 µg/mL) and **B5** (256 µg/mL) for *E. faecalis*. Therefore, these compounds should be excluded when considering the positive effect of naringenin oximes on the human gut microbiome.

### 2.3. Anticancer Activity

#### 2.3.1. Antiproliferative Activity (SRB and MTT Assays)

The anticancer effect of the synthesized naringenin derivatives was investigated on HT-29 colon cancer cells using the 3-(4,5-dimethylthiazol-2-yl)-2,5-diphenyl-2H-tetrazolium bromide (MTT) and sulforhodamine B (SRB) assays. Furthermore, the antiproliferative activity against the mouse fibroblast 3T3-L1 cell line was also determined. The cytotoxicity of all compounds tested by the SRB assay is presented in terms of IC_50_ values in Table 3. We noticed that the attachment of the *O*-hexyl, *O*-heptyl, *O*-octyl, *O*-nonyl or *O*-undecyl chain to the C-7 position improved the anticancer effect of naringenin (for compounds **A1**, **A3**, **A5**, **A7** and **A9** IC_50_ in the range of 14.19–17.85 µg/mL). It is worth noting that chemical modifications of 7-*O*-alkyl derivatives of naringenin, leading to their oximes, resulted in slightly better activity against HT-29 cells (for compounds **B1**, **B3**, **B5**, **B7**, **B9**, IC_50_ values in the range of 9.96–11.42 µg/mL). The most potent anticancer effect was determined for **B5** (IC_50_ 9.96 ± 1.13 µg/mL), followed by **B1**, **B3**, **B7** and **B9**. On the other hand, the incorporation of the NOH moiety into 7-*O*-undecylnaringenin (**A9**) resulted in increased antiproliferative activity, from an IC_50_ value of 17.85 ± 0.65 µg/mL to 11.42 ± 1.17 µg/mL (for compound **B9**). The best improvement in cytotoxicity was observed for 7,4′-di-*O*-hexylnaringenin oxime (**B2**), which was characterized by more than two-times higher anticancer activity in comparison to its *O*-alkyl derivative (IC_50_ for compound **A2** > 100 µg/mL and for **B2** IC_50_ = 49.76 ± 1.63 µg/mL). However, other 7,4′-di-*O*-alkyl derivatives, namely **A4**, **A6**, **A8** and **A10** and their oximes **B4**, **B6**, **B8** and **B10**, had no effect on the proliferation of the HT-29 cell line. Earlier studies have established that HT-29 is one of the most naringenin-resistant colon adenocarcinoma cell lines [33]. This statement was confirmed in our research, in which naringenin showed only a slight cytotoxic effect on the colorectal cancer cell line in the tested concentration range (IC_50_ > 100 µg/mL).

Unfortunately, active *O*-alkyl derivatives and their oximes against the HT-29 colon cancer cell line were not inactive toward mouse fibroblasts 3T3-L1. Naringenin, at a high, nonphysiological concentration, has been shown to have a limited effect on 3T3 cell proliferation and viability [34,35]. Interestingly, the concentration of 100 µM even decreased the p53 activity slightly in NIH 3T3 cells, although the same amount of naringenin reduced the activity of kinase AKT and inhibited insulin-stimulated glucose uptake as well as the adipogenesis of 3T3-L1 cells [36,37,38]. The attachment of an oxime moiety to naringenin led to an increase in cytotoxicity, but only against 3T3-L1 cells. Although Kocyigit et al. observed stronger effects of **NGOX** on HT-29 cells, the IC_50_ value was 175 µg/mL, well above our range [29].

**Figure 1:**
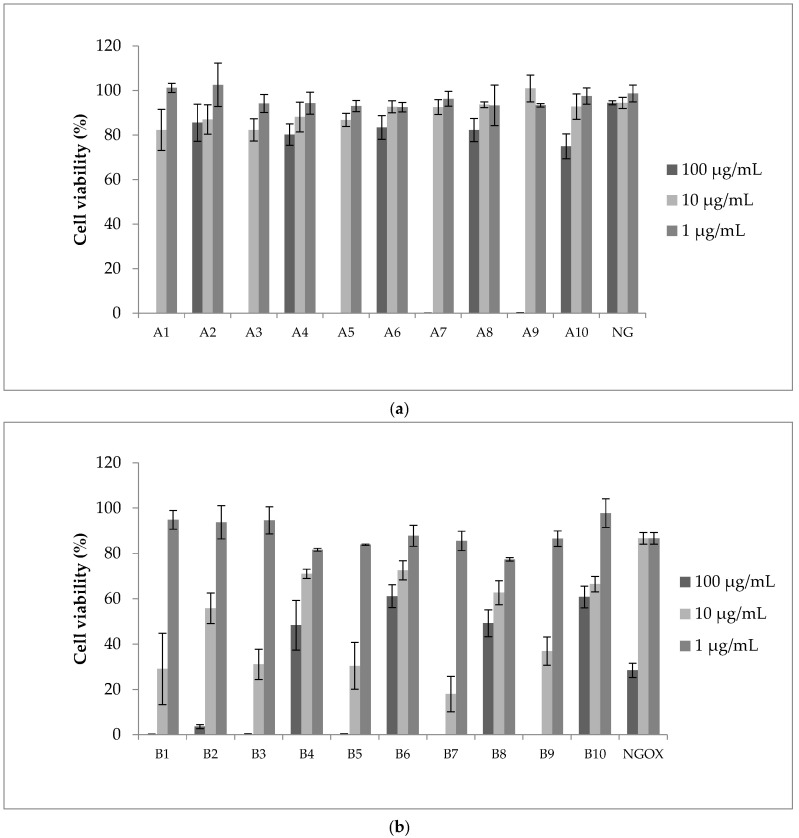
The viability of HT-29 cells treated with derivatives of naringenin from three independent MTT experiments is presented as a percentage of untreated cells: (**a**) viability of cells after *O*-alkyl derivative treatment, (**b**) viability cells after oxime derivative treatment.

The viability of the HT-29 and 3T3-L1 cell lines was determined using the MTT assay in three concentrations: 100, 10 and 1 µg/mL. We noticed that colon adenocarcinoma cells treated with *O*-alkyl derivatives of naringenin **A1**, **A3**, **A5**, **A7** and **A9** (Figure 1a) were completely inhibited only at the highest concentration. However, even at this dose, the effect of naringenin and its derivatives **A2**, **A4**, **A6**, **A8**, **A10** was weak. On the other hand, attaching the oxime group reduced cell viability by more than 50% for most compounds, including **NGOX**. In addition, a more than 50% reduction in cell viability was possible with the 10 µg/mL dose of compounds **B1**, **B3**, **B5**, **B7**, **B9**.

#### 2.3.2. Apoptosis

The balance of the human gut microbiota helps to maintain homeostasis and protects against intestinal disorders, including the development of cancer [39]. Thus, new anticancer drugs should characterized by strong activity against cancer cells and should be safe for the intestinal microflora. Based on the results of naringenin derivatives on the human gut microbiota (MIC and MBC assays) and after the evaluation of the antiproliferative activity of all compounds on the HT-29 and 3T3-L1 cell lines, we selected four derivatives to determine their mechanisms of action. We chose inactive compound **A2** (for HT-29 and 3T3-L1 IC_50_ > 100 µg/mL) and its oxime **B2**, which exhibited cytotoxic effects on HT-29 (IC_50_ = 49.76 ± 1.63 µg/mL), and also compounds **A9** (for HT-29 IC_50_ = 17.85 ± 0.65 µg/mL) and **B9** (for HT-29 IC_50_ = 11.42 ± 1.17 µg/mL). None of the compounds inhibited the growth of *S. aureus* ATCC 25923 and *E. coli* K12 (MIC and MBC value > 512 mg/mL). In the case of *E. faecalis* ATCC 29212, the activity varied, as shown in Table 1.

**Figure 2:**
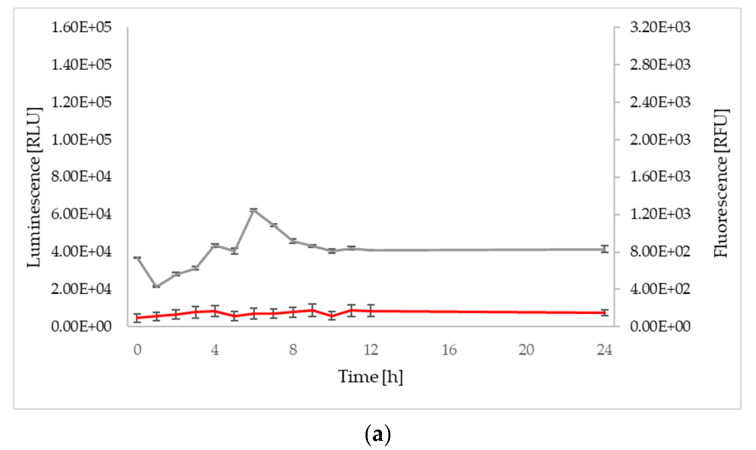

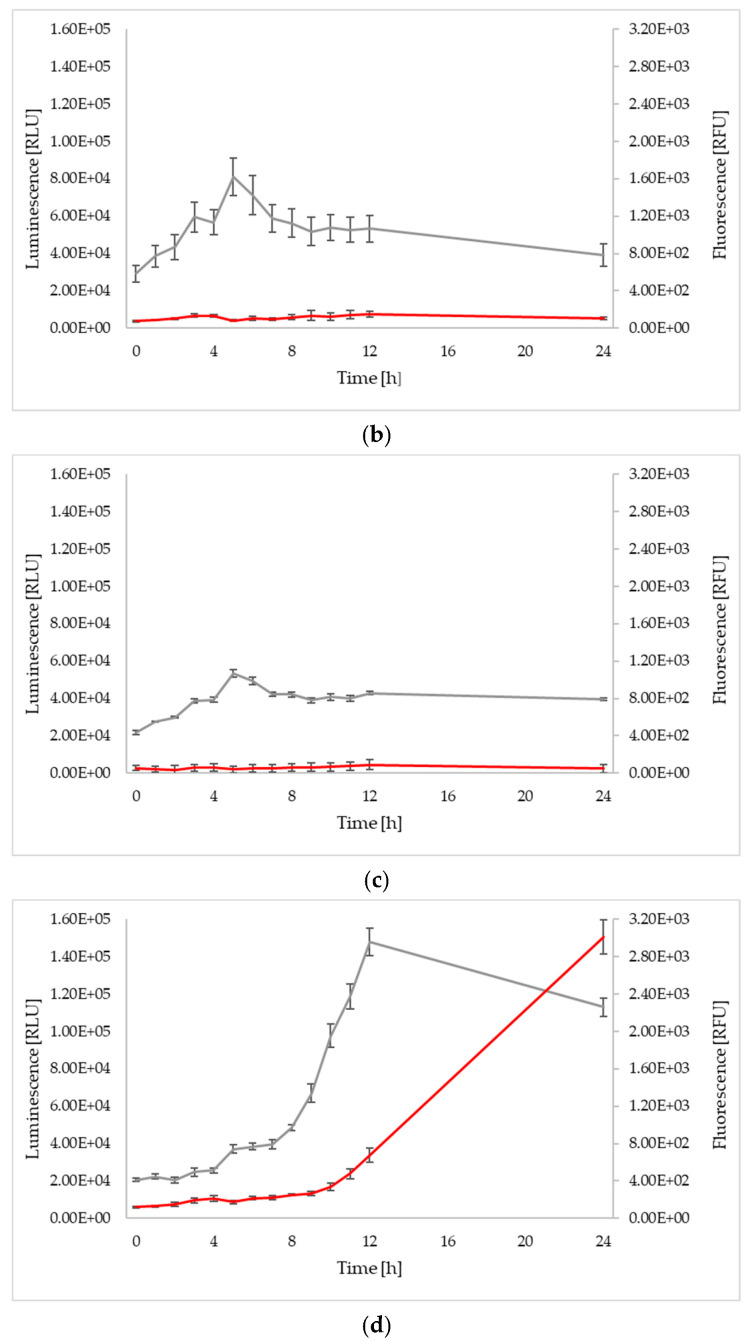
The apoptosis assay. HT-29 cells were treated with 50 µg/mL of naringenin derivatives ((**a**) **A2**, (**b**) **B2**, (**c**) **A9**, (**d**) **B9**), and the RealTime-Glo Annexin V Apoptosis and Necrosis Assay was performed during 24 h incubation. The grey line represents the luminescence signal from annexin V, whereas the red line shows the fluorescence of the necrosis reagent.

Apoptosis is a natural process of genetically programmed cell death. It is necessary for the proper functioning of eukaryotic cells in physiological and pathological processes, including tumorigenesis. Taking into account that the morphological changes occurring in apoptotic cells are accompanied by biochemical changes that primarily affect the cytoplasmic membrane, an assay examining the level of phosphatidylserine was used for the study. This negatively charged anionic phospholipid moves to the outer layer during apoptosis. For this purpose, we tested compounds **A2**, **B2**, **A9**, **B9**, **NG** and **NGOX** at concentrations of 10, 25, 50 and 100 µg/mL. In order to investigate the mechanism of cytotoxicity of the selected derivatives, the RealTime-Glo Annexin V Apoptosis and Necrosis Assay was performed, as presented in Figure 2. We demonstrated that phosphatidylserine exposure (luminescent signal) on the outer side of the cell membrane significantly increased only after treating HT-29 cells with compound **B9** at a concentration of 50 µg/mL (Figure 2d). Indeed, in this particular case, the observed onset of loss of membrane integrity (manifested by the fluorescence signal) was time-shifted with respect to luminescence and occurred more than 5 h later. This confirmed the appearance of apoptosis in the cancer cells tested.

In the case of **A2**, **B2**, **A9**, **NG** and **NGOX**, both signals were similar to those for untreated cells during 12 h incubation. Thus, the mechanism of their cytotoxicity may not involve apoptosis at the applied concentration and time range. Although Kocyigit et al. showed cytotoxicity and apoptosis in HT-29 cells induced by both **NG** and **NGOX** for concentrations of 100 µM and lower, this was not confirmed by Shen et al. or our observations for this range of **NG** doses [29,40]. Furthermore, Lozano-Herrera et al. identified apoptosis as well as necrosis in HT-29 cells after 24 h of treatment with **NG**, but they used a higher concentration of 250 µM, which was still lower than the IC_50_ indicated in our investigation [41]. The pro-apoptotic activity of **NG** has also been demonstrated for other colon adenocarcinoma cell lines, as well as various types of cancer, indicating its interaction with estrogen receptors α and/or β (ESR1, ESR2) and the activation of the p38/MAPK pathway [42].

To confirm the process of apoptosis in HT-29 cells treated with the tested compounds, caspase 3/7 activity was determined using the Caspase-Glo 3/7 Assay. Caspases 3 and 7 belong to the executive caspase subfamily. They are activated in both the intrinsic (fusion of procaspase 9 with the apoptosome) and extrinsic (autoproteolytic activation of procaspase 8) pathways of apoptosis. Moreover, caspase 3 is the most important of all caspases, as it cleaves many proteins crucial for cell skeleton, cell cycle, and maturing mRNA. [43].

**Figure 3:**
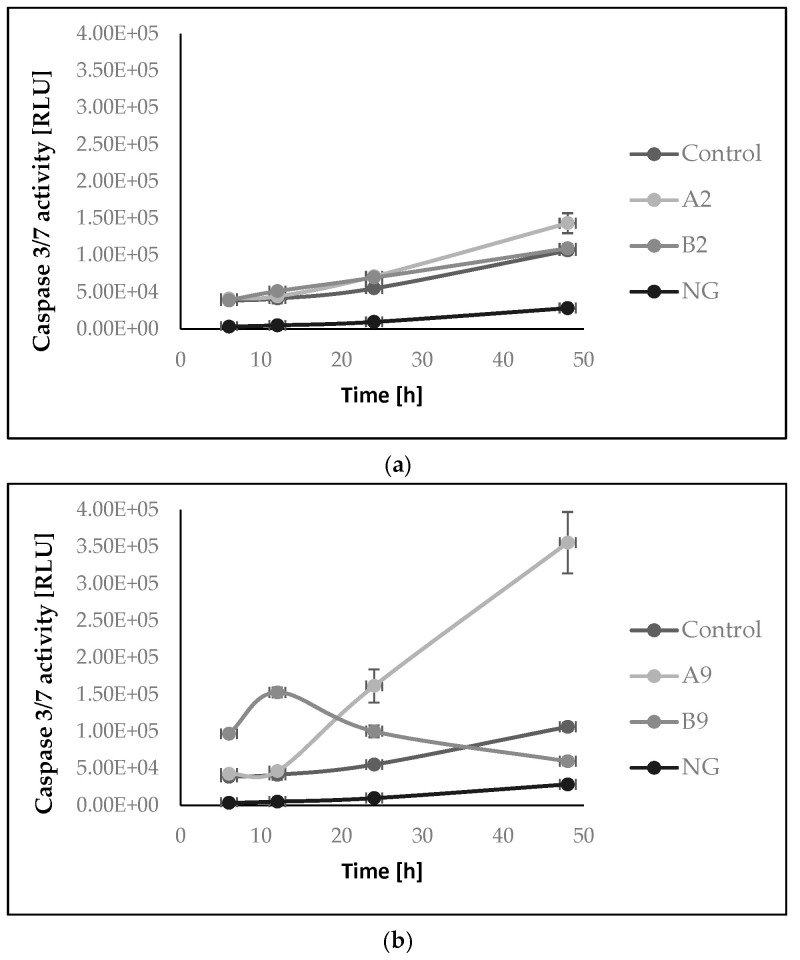
The caspase 3/7 activity. HT-29 cells were treated with 50 µg/mL naringenin and its derivatives ((**a**) **A2** and **B2** (**b**) **A9** and **B9**), and after 6, 12, 24 and 48 h, the Caspase-Glo 3/7 Assay was performed. The results represent luminescence signals—RLU.

Here, we observed caspase 3/7 activation within the first 12 h of incubation, which confirmed the rapid process of apoptosis in HT-29 cells treated with **B9** (Figure 3b). Interestingly, significant caspase 3/7 activation was also detected after **A9** treatment, although the process was slower compared to that with its oxime derivative **B9**. This was likely because the phosphatidylserine signal was not detected during the first 24 h of incubation with **A9**. Similarly, for **NG**, **A2** and **B2,** neither membrane switching of phosphatidylserine nor caspase activation was observed. In our study, the **B9** derivative was the most effective in the activation of apoptosis, while **NG** did not induce apoptosis at the same concentration.

Based on the results, we found that compound **B9** at a concentration of 50 µg/mL could effectively activate programmed cell death pathways in HT-29 cells.

## 3. Materials and Methods

### 3.1. Chemistry

#### 3.1.1. Chemicals

Naringenin and alkyl iodides (1-iodoheptane, 1-iodooctane, 1-iodononane, 1-iodoundecane) were purchased from Sigma-Aldrich Co. (St. Louis, MO, USA); hydroxylamine hydrochloride from LOBA Feinchemie GmbH (Fischamed, Austria); anhydrous sodium acetate, potassium carbonate and *N*,*N*-dimethylformamide (DMF) from Chempur (Piekary Śląskie, Poland).

Anhydrous ethanol for reactions was prepared according to standard procedures. All organic solvents used for extraction and purification (diethyl ether, hexane, ethyl acetate, methylene chloride, chloroform, methanol) were of analytical grade and were purchased from Stanlab (Lublin, Poland).

#### 3.1.2. Analytical Methods

To monitor the progress of reactions, thin layer chromatography (TLC) on silica gel-coated aluminum sheets with a fluorescent indicator was performed (DC-Alufolien, Kieselgel 60 F254; Merck, Darmstadt, Germany). Then, TLC plates were observed under a UV lamp at the wavelengths of 366 nm and 254 nm, and sprayed with a solution of 1% cerium(IV) sulfate and 2% phosphomolybdic acid in 5% sulfuric(VI) acid to visualize the synthesized compounds. Crude reaction products were separated and purified by liquid column chromatography using Kieselgel 60, 230–400-mesh (Merck). The structures of the *O*-alkyl derivatives and their oximes were determined using ^1^H, ^13^C, ^1^H-^1^H (COSY) and ^1^H-^13^C (HSQC) nuclear magnetic resonance (NMR) spectra, which were recorded on a Bruker AvanceTM600 MHz spectrometer (Bruker, Billerica, MA, USA). Samples for NMR analysis were prepared using chloroform-*d* (compounds **A3**–**A10**) and acetone-*d*_6_ (compounds **B3**–**B10**) (Supplementary Materials, Figures S1–S64).

To confirm the molar masses of the synthesized derivatives, high-resolution ESI-MS spectra were measured on a Bruker ESI-Q-TOF Maxis Impact Mass Spectrometer (Bruker). The direct infusion of ESI-MS parameters: the mass spectrometer was operated in positive ion mode with the potential between the spray needle and the orifice as 4.0 kV, a nebulizer pressure of 0.4 bar and a drying gas flow rate of 3.0 L/min at 200 °C. The sample flow rate was 3.0 µL/min. Ionization mass spectra were collected in the range of *m*/*z* 50–1350.

The melting points (uncorrected) were determined with a Boetius apparatus (Jena, Germany).

#### 3.1.3. Synthesis of Ether Naringenin Derivatives **A3**–**A10**

Naringenin (3.68 mmol) was weighed into a round-bottom flask and dissolved in 10 mL of DMF. Then, anhydrous potassium carbonate (5.52 mmol) and an appropriate amount of alkyl iodide (18.40 mmol) were added. Reactions were performed on a magnetic stirrer at room temperature. After the reaction was completed (17–18 h), 20 mL of distilled water and 10 mL of saturated solution of sodium chloride were added, and triplicate extraction with diethyl ether (3 × 50 mL) was carried out. Collected organic fractions were dried over anhydrous sodium sulfate and concentrated on a vacuum evaporator. The crude extract, containing mono- and di-*O*-alkyl derivatives, was separated and purified by liquid column chromatography using a mixture of hexane:methylene chloride:ethyl acetate (5:1:1 *v*:*v*:*v*) as an eluent. Further purification of di-*O*-alkyl derivatives was performed using the same mixture of organic solvents with a volumetric composition of 15:1:1. The spectroscopic and physical data of obtained derivatives **A3**–**A10** are presented below, while those of 7-*O*-hexylnaringenin (**A1**) and 7,4′-di-*O*-hexylnaringenin (**A2**) were presented in our previous work [32].

7-*O*-Heptylnaringenin (**A3**), white powder, ^1^H NMR (600 MHz, chloroform-*d*) δ 12.01 (s, 1H, OH-5), 7.35–7.30 (m, 2H, AA’BB’, H-2′, H-6′), 6.91–6.85 (m, 2H, AA’BB’, H-3′, H-5′), 6.06 (d, *J* = 2.3 Hz, 1H, H-6), 6.03 (d, *J* = 2.3 Hz, 1H, H-8), 5.38–5.30 (m, 2H, H-2 and OH-4′), 3.96 (t, *J* = 6.6 Hz, 2H, -CH_2_-), 3.08 (dd, *J* = 17.1, 13.0 Hz, 1H, H-3a), 2.78 (dd, *J* = 17.1, 3.0 Hz, 1H, H-3b), 1.80–1.73 (m, 2H, -CH_2_-), 1.44–1.38 (m, 2H, -CH_2_-), 1.36–1.27 (m, 6H, 3x-CH_2_-), 0.89 (t, *J* = 6.9 Hz, 3H, -CH_3_); ^13^C NMR (150 MHz, chloroform-*d*) δ 196.17 (C=O), 167.85, 164.19, 163.02, 156.28, 130.70, 128.09, 115.81, 103.13, 95.73, 94.78, 79.05, 68.74, 43.30, 31.86, 29.08, 29.02, 25.98, 22.72, 14.21; HRMS (*m*/*z*): [M + H]^+^ calculated for C_22_H_27_O_5_, 371.1853; found 371.1854.
7,4′-Di-*O*-heptylnaringenin (**A4**), pale yellow powder, ^1^H NMR (600 MHz, chloroform-*d*) δ 12.02 (s, 1H, OH-5), 7.39–7.32 (m, 2H, AA’BB’, H-2′, H-6′), 6.96–6.91 (m, 2H, AA’BB’, H-3′, H-5′), 6.05 (d, *J* = 2.3 Hz, 1H, H-6), 6.03 (d, *J* = 2.3 Hz, 1H, H-8), 5.35 (dd, *J* = 13.0, 3.0 Hz, 1H, H-2), 3.99–3.94 (m, 4H, 2x-CH_2_-), 3.09 (dd, *J* = 17.1, 13.0 Hz, 1H, H-3a), 2.78 (dd, *J* = 17.1, 3.0 Hz, 1H, H-3b), 1.82–1.73 (m, 4H, 2x-CH_2_-), 1.49–1.28 (m, 16H, 8x-CH_2_-), 0.92–0.87 (m, 6H, 2x-CH_3_); ^13^C NMR (150 MHz, chloroform-*d*) δ 196.11 (C=O), 167.74, 164.23, 163.04, 159.77, 130.33, 127.83, 114.91, 103.16, 95.67, 94.71, 79.16, 68.70, 68.29, 43.35, 31.93, 31.87, 29.36, 29.20, 29.10, 29.04, 26.14, 26.00, 22.76, 22.73, 14.24, 14.22; HRMS (*m*/*z*): [M + H]^+^ calculated for C_29_H_41_O_5_, 469.2949; found 469.2947.
7-*O*-Octylnaringenin (**A5**), white powder, ^1^H NMR (600 MHz, chloroform-*d*) δ 12.01 (s, 1H, OH-5), 7.36–7.30 (m, 2H, AA’BB’, H-2′, H-6′), 6.91–6.85 (m, 2H, AA’BB’, H-3′, H-5′), 6.06 (d, *J* = 2.3 Hz, 1H, H-6), 6.03 (d, *J* = 2.3 Hz, 1H, H-8), 5.35 (dd, *J* = 13.0, 3.0 Hz, 1H, H-2), 5.16 (s, 1H, OH-4′), 3.96 (t, *J* = 6.6 Hz, 2H, -CH_2_-), 3.08 (dd, *J* = 17.2, 13.0 Hz, 1H, H-3a), 2.78 (dd, *J* = 17.2, 3.0 Hz, 1H, H-3b), 1.76 (p, *J* = 6.6 Hz, 2H, -CH_2_-), 1.45–1.38 (m, 2H, -CH_2_-), 1.36–1.26 (m, 8H, 4x-CH_2_-), 0.88 (t, *J* = 6.9 Hz, 3H, -CH_3_); ^13^C NMR (150 MHz, chloroform-*d*) δ 196.12 (C=O), 167.82, 164.21, 163.01, 156.23, 130.77, 128.10, 115.81, 103.14, 95.73, 94.77, 79.05, 68.74, 43.33, 31.92, 29.39, 29.33, 29.03, 26.03, 22.78, 14.24; HRMS (*m*/*z*): [M + H]^+^ calculated for C_23_H_29_O_5_, 385.2010; found 385.2005.
7,4′-Di-*O*-octylnaringenin (**A6**), pale yellow powder, ^1^H NMR (600 MHz, chloroform-*d*) δ 12.02 (s, 1H, OH-5), 7.38–7.34 (m, 2H, AA’BB’, H-2′, H-6′), 6.96–6.92 (m, 2H, AA’BB’, H-3′, H-5′), 6.05 (d, *J* = 2.3 Hz, 1H, H-6), 6.02 (d, *J* = 2.3 Hz, 1H, H-8), 5.35 (dd, *J* = 13.0, 3.0 Hz, 1H, H-2), 3.99–3.94 (m, 4H, 2x-CH_2_-), 3.09 (dd, *J* = 17.1, 13.0 Hz, 1H, H-3a), 2.78 (dd, *J* = 17.1, 3.0 Hz, 1H, H-3b), 1.81–1.73 (m, 4H, 2x-CH_2_-), 1.48–1.39 (m, 4H, 2x-CH_2_-), 1.37–1.26 (m, 16H, 8x-CH_2_-), 0.92–0.86 (m, 6H, 2x-CH_3_); ^13^C NMR (150 MHz, chloroform-*d*) δ 196.11 (C=O), 167.73, 164.23, 163.04, 159.76, 130.33, 127.82, 114.90, 103.15, 95.67, 94.71, 79.15, 68.70, 68.29, 43.35, 31.96, 31.93, 29.49, 29.39, 29.36, 29.33, 29.04, 26.18, 26.04, 22.81, 22.79, 14.25, 14.24; HRMS (*m*/*z*): [M + H]^+^ calculated for C_31_H_45_O_5_, 497.3262; found 497.3255.
7-*O*-Nonylnaringenin (**A7**), white powder, ^1^H NMR (600 MHz, chloroform-*d*) δ 12.01 (s, 1H, OH-5), 7.33 (d, *J* = 8.1 Hz, 2H, H-2′, H-6′), 6.88 (d, *J* = 8.1 Hz, 2H, H-3′, H-5′), 6.06 (d, *J* = 2.2 Hz, 1H, H-6), 6.03 (d, *J* = 2.2 Hz, 1H, H-8), 5.35 (dd, *J* = 13.1, 2.9 Hz, 1H, H-2), 5.03 (s, 1H, OH-4′), 3.96 (t, *J* = 6.6 Hz, 2H, -CH_2_-), 3.08 (dd, *J* = 17.2, 13.1 Hz, 1H, H-3a), 2.78 (dd, *J* = 17.2, 2.9 Hz, 1H, H-3b), 1.76 (p, *J* = 6.6 Hz, 2H, -CH_2_-), 1.45–1.38 (m, 2H, -CH_2_-), 1.35–1.25 (m, 10H, 5x-CH_2_-), 0.88 (t, *J* = 6.9 Hz, 3H, -CH_3_); ^13^C NMR (150 MHz, chloroform-*d*) δ 196.07 (C=O), 167.80, 164.23, 162.99, 156.20, 130.84, 128.10, 115.81, 103.14, 95.72, 94.76, 79.05, 68.74, 43.36, 32.00, 29.63, 29.43, 29.38, 29.03, 26.03, 22.81, 14.25; HRMS (*m*/*z*): [M + H]^+^ calculated for C_24_H_31_O_5_, 399.2166; found 399.2162.
7,4′-Di-*O*-nonylnaringenin (**A8**), pale yellow powder, ^1^H NMR (600MHz, chloroform-*d*) δ 12.02 (s, 1H, OH-5), 7.38–7.33 (m, 2H, AA’BB’, H-2′, H-6′), 6.97–6.91 (m, 2H, AA’BB’, H-3′, H-5′), 6.05 (d, *J* = 2.3 Hz, 1H, H-6), 6.02 (d, *J* = 2.3 Hz, 1H, H-8), 5.35 (dd, *J* = 13.1, 3.0 Hz, 1H, H-2), 3.99–3.94 (m, 4H, 2x-CH_2_-), 3.09 (dd, *J* = 17.1, 13.1 Hz, 1H, H-3a), 2.78 (dd, *J* = 17.1, 3.0 Hz, 1H, H-3b), 1.83–1.72 (m, 4H, 2x-CH_2_-), 1.48–1.38 (m, 4H, 2x-CH_2_-), 1.37–1.25 (m, 20H, 10x-CH_2_-), 0.91–0.86 (m, 6H, 2x-CH_3_); ^13^C NMR (150 MHz, chloroform-*d*) δ 196.11 (C=O), 167.74, 164.23, 163.05, 159.76, 130.33, 127.83, 114.91, 103.16, 95.68, 94.72, 79.16, 68.71, 68.30, 43.36, 32.03, 32.00, 29.69, 29.63, 29.54, 29.44, 29.41, 29.38, 29.36, 29.05, 26.18, 26.04, 22.82, 14.26; HRMS (*m*/*z*): [M + H]^+^ calculated for C_33_H_49_O_5_, 525.3575; found 525.3568.
7-*O*-Undecylnaringenin (**A9**), white powder, ^1^H NMR (600 MHz, chloroform-*d*) δ 12.01 (s, 1H, OH-5), 7.35–7.30 (m, 2H, AA’BB’, H-2′, H-6′), 6.91–6.86 (m, 2H, AA’BB’, H-3′, H-5′), 6.06 (d, *J* = 2.2 Hz, 1H, H-6), 6.03 (d, *J* = 2.2 Hz, 1H, H-8), 5.37 (s, 1H, OH-4′), 5.34 (dd, *J* = 13.0, 3.0 Hz, 1H, H-2), 3.95 (t, *J* = 6.6 Hz, 2H, -CH_2_-), 3.08 (dd, *J* = 17.1, 13.0 Hz, 1H, H-3a), 2.78 (dd, *J* = 17.1, 3.0 Hz, 1H, H-3b), 1.78–1.73 (m, 2H, -CH_2_-), 1.44–1.38 (m, 2H, -CH_2_-), 1.36–1.25 (m, 14H, 7x-CH_2_-), 0.88 (t, *J* = 7.0 Hz, 3H, -CH_3_); ^13^C NMR (150 MHz, chloroform-*d*) δ 196.17 (C=O), 167.85, 164.19, 163.02, 156.29, 130.69, 128.09, 115.81, 103.13, 95.72, 94.78, 79.05, 68.75, 43.30, 32.04, 29.74, 29.71, 29.66, 29.47, 29.42, 29.02, 26.02, 22.82, 14.26; HRMS (*m*/*z*): [M + H]^+^ calculated for C_26_H_35_O_5_, 427.2479; found 427.2472.
7,4′-Di-*O*-undecylnaringenin (**A10**), pale yellow powder, ^1^H NMR (600 MHz, chloroform-*d*) δ 12.02 (s, 1H, OH-5), 7.38–7.34 (m, 2H, AA’BB’, H-2′, H-6′), 6.96–6.92 (m, 2H, AA’BB’, H-3′, H-5′), 6.05 (d, *J* = 2.3 Hz, 1H, H-6), 6.02 (d, *J* = 2.3 Hz, 1H, H-8), 5.35 (dd, *J* = 13.0, 3.0 Hz, 1H, H-2), 3.99–3.94 (m, 4H, 2x-CH_2_-), 3.09 (dd, *J* = 17.1, 13.0 Hz, 1H, H-3a), 2.78 (dd, *J* = 17.1, 3.0 Hz, 1H, H-3b), 1.82–1.73 (m, 4H, 2x-CH_2_-), 1.48–1.39 (m, 4H, 2x-CH_2_-), 1.37–1.25 (m, 28H, 14x-CH_2_-), 0.91–0.86 (m, 6H, 2x-CH_3_); ^13^C NMR (150 MHz, chloroform-*d*) δ 196.10 (C=O), 167.73, 164.23, 163.04, 159.76, 130.33, 127.82, 114.90, 103.15, 95.67, 94.71, 79.16, 68.70, 68.29, 43.35, 32.06, 29.77, 29.75, 29.72, 29.67, 29.54, 29.49, 29.48, 29.43, 29.36, 29.04, 26.18, 26.03, 22.84, 14.27; HRMS (*m*/*z*): [M + H]^+^ calculated for C_37_H_57_O_5_, 581.4201; found 581.4187.

#### 3.1.4. Synthesis of Oximes **B3**–**B10**

In a round-bottom flask, the *O*-alkyl derivative of naringenin was dissolved in the smallest amount of anhydrous ethanol (5–10 mL), and the hydroxylamine hydrochloride and anhydrous sodium acetate were added in a molar ratio of 1:3:3, respectively. The reactions were conducted on a magnetic stirrer at 45 °C for 17–27 h. After the complete reaction of the *O*-alkyl derivative of naringenin, the reaction mixture was poured into ice water, and the precipitated crystals were filtered under reducing pressure on a Büchner funnel and dried. The crude product of the reaction was purified via liquid column chromatography using a mixture of chloroform:methanol (96:4 *v*:*v* for compounds **B3**, **B7**, **B9**; 97:2 *v*:*v* for compound **B5**; and 20:0.1 *v*:*v* for compounds **B4**, **B6**, **B8** and **B10**) as the eluent. The spectroscopic and physical data of obtained derivatives **B3**–**B10** are presented below; those for 7-*O*-hexylnaringenin oxime (**B1**) and 7,4′-di-*O*-hexylnaringenin oxime (**B2**) were presented in our previous work [32].

7-*O*-Heptylnaringenin oxime (**B3**), white powder, ^1^H NMR (600 MHz, acetone-*d*_6_) δ 11.01 (s, 1H, NOH), 10.40 (s, 1H, OH-5), 8.49 (s, 1H, OH-4′), 7.40–7.36 (m, 2H, AA’BB’, H-2′, H-6′), 6.92–6.86 (m, 2H, AA’BB’, H-3′, H-5′), 6.05 (d, *J* = 2.4 Hz, 1H, H-6), 6.03 (d, *J* = 2.4 Hz, 1H, H-8), 5.07 (dd, *J* = 12.0, 3.2 Hz, 1H, H-2), 3.96 (t, *J* = 6.6 Hz, 2H, -CH_2_-), 3.46 (dd, *J* = 17.1, 3.2 Hz, 1H, H-3a), 2.79 (dd, *J* = 17.1, 12.0 Hz, 1H, H-3b), 1.78–1.70 (m, 2H, -CH_2_-), 1.49–1.43 (m, 2H, -CH_2_-), 1.39–1.29 (m, 6H, 3x-CH_2_-), 0.89 (t, *J* = 6.9 Hz, 3H, -CH_3_); ^13^C NMR (150 MHz, acetone-*d*_6_) δ 162.95, 160.62, 159.41, 158.46, 154.85 (C=NOH), 131.75, 128.79, 116.12, 99.14, 96.58, 95.11, 77.37, 68.66, 32.53, 30.30, 29.74, 26.67, 23.26, 14.33; HRMS (*m*/*z*): [M + H]^+^ calculated for C_22_H_28_NO_5_, 386.1962; found 386.1958.
7,4′-Di-*O*-heptylnaringenin oxime (**B4**), white powder, ^1^H NMR (600 MHz, acetone-*d*_6_) δ 11.01 (s, 1H, NOH), 10.38 (s, 1H, OH-5), 7.47–7.44 (m, 2H, AA’BB’, H-2′, H-6′), 7.00–6.96 (m, 2H, AA’BB’, H-3′, H-5′), 6.05 (d, *J* = 2.4 Hz, 1H, H-6), 6.04 (d, *J* = 2.4 Hz, 1H, H-8), 5.11 (dd, *J* = 11.9, 3.2 Hz, 1H, H-2), 4.02 (t, *J* = 6.5 Hz, 2H, -CH_2_-), 3.97 (t, *J* = 6.5 Hz, 2H, -CH_2_-), 3.47 (dd, *J* = 17.1, 3.2 Hz, 1H, H-3a), 2.81 (dd, *J* = 17.1, 11.9 Hz, 1H, H-3b), 1.81–1.72 (m, 4H, 2x-CH_2_-), 1.52–1.42 (m, 4H, 2x-CH_2_-), 1.41–1.30 (m, 12H, 6x-CH_2_-), 0.92–0.86 (m, 6H, 2x-CH_3_); ^13^C NMR (150 MHz, acetone-*d*_6_) δ 162.08, 159.75, 159.35, 158.44, 153.88 (C=NOH), 131.84, 127.77, 114.38, 98.26, 95.73, 94.25, 76.34, 67.78, 67.71, 31.68, 31.66, 29.39, 29.14, 28.91, 28.86, 25.86, 25.79, 22.40, 22.38, 13.45; HRMS (*m*/*z*): [M + H]^+^ calculated for C_29_H_42_NO_5_, 484.3057; found 484.3046.
7-*O*-Octylnaringenin oxime (**B5**), white powder, ^1^H NMR (600 MHz, acetone-*d*_6_) δ 11.01 (s, 1H, NOH), 10.39 (s, 1H, OH-5), 8.49 (s, 1H, OH-4′), 7.41–7.36 (m, 2H, AA’BB’, H-2′, H-6′), 6.92–6.87 (m, 2H, AA’BB’, H-3′, H-5′), 6.05 (d, *J* = 2.4 Hz, 1H, H-6), 6.03 (d, *J* = 2.4 Hz, 1H, H-8), 5.07 (dd, *J* = 12.0, 3.2 Hz, 1H, H-2), 3.97 (t, *J* = 6.5 Hz, 2H, -CH_2_-), 3.46 (dd, *J* = 17.1, 3.2 Hz, 1H, H-3a), 2.79 (dd, *J* = 17.1, 12.0 Hz, 1H, H-3b), 1.77–1.71 (m, 2H, -CH_2_-), 1.48–1.42 (m, 2H, -CH_2_-), 1.39–1.28 (m, 8H, 4x-CH_2_-), 0.88 (t, *J* = 7.0 Hz, 3H, -CH_3_); ^13^C NMR (150 MHz, acetone-*d*_6_) δ 162.95, 160.62, 159.41, 158.46, 154.85 (C=NOH), 131.75, 128.79, 116.12, 99.14, 96.59, 95.11, 77.37, 68.66, 32.55, 30.30, 30.03, 26.71, 23.30, 14.35; HRMS (*m*/*z*): [M + H]^+^ calculated for C_23_H_30_NO_5_, 400.2118; found 400.2114.
7,4′-Di-*O*-octylnaringenin oxime (**B6**), white powder, ^1^H NMR (600 MHz, acetone-*d*_6_) δ 11.01 (s, 1H, N=OH), 10.42 (s, 1H, OH-5), 7.48–7.43 (m, 2H, AA’BB’, H-2′, H-6′), 7.00–6.95 (m, 2H, AA’BB’, H-3′, H-5′), 6.05 (d, *J* = 2.4 Hz, 1H, H-6), 6.04 (d, *J* = 2.4 Hz, 1H, H-8), 5.11 (dd, *J* = 11.9, 3.2 Hz, 1H, H-2), 4.02 (t, *J* = 6.5 Hz, 2H, -CH_2_-), 3.97 (t, *J* = 6.5 Hz, 2H, -CH_2_-), 3.47 (dd, *J* = 17.1, 3.2 Hz, 1H, H-3a), 2.80 (dd, *J* = 17.1, 11.9 Hz, 1H, H-3b), 1.82–1.70 (m, 4H, 2x-CH_2_-), 1.53–1.41 (m, 4H, 2x-CH_2_-), 1.40–1.28 (m, 16H, 8x-CH_2_-), 0.91–0.86 (m, 6H, 2x-CH_3_); ^13^C NMR (150 MHz, acetone-*d*_6_) δ 162.96, 160.63, 160.23, 159.32, 154.73 (C=NOH), 132.72, 128.66, 115.27, 99.15, 96.61, 95.14, 77.22, 68.67, 68.60, 32.57, 32.56, 30.28, 30.03, 30.02, 26.79, 26.71, 23.32, 23.31, 14.36; HRMS (*m*/*z*): [M + H]^+^ calculated for C_31_H_46_NO_5_, 512.3370; found 512.3359.
7-*O*-Nonylnaringenin oxime (**B7**), white powder, ^1^H NMR (600 MHz, acetone-*d*_6_) δ 11.01 (s, 1H, NOH), 10.37 (s, 1H, OH-5), 8.46 (s, 1H, OH-4′), 7.41–7.36 (m, 2H, AA’BB’, H-2′, H-6′), 6.92–6.87 (m, 2H, AA’BB’, H-3′, H-5′), 6.05 (d, *J* = 2.4 Hz, 1H, H-6), 6.03 (d, *J* = 2.4 Hz, 1H, H-8), 5.07 (dd, *J* = 12.0, 3.1 Hz, 1H, H-2), 3.97 (t, *J* = 6.6 Hz, 2H, -CH_2_-), 3.46 (dd, *J* = 17.1, 3.1 Hz, 1H, H-3a), 2.79 (dd, *J* = 17.1, 12.0 Hz, 1H, H-3b), 1.77–1.71 (m, 2H, -CH_2_-), 1.48–1.42 (m, 2H, -CH_2_-), 1.40–1.27 (m, 10H, 5x-CH_2_-), 0.88 (t, *J* = 7.0 Hz, 3H, -CH_3_); ^13^C NMR (150 MHz, acetone-*d*_6_) δ 162.96, 160.63, 159.42, 158.46, 154.88 (C=NOH), 131.77, 128.79, 116.12, 99.13, 96.60, 95.12, 77.38, 68.66, 32.60, 30.31, 30.28, 30.07, 30.00, 26.70, 23.32, 14.35; HRMS (*m*/*z*): [M + H]^+^ calculated for C_24_H_32_NO_5_, 414.2275; found 414.2268.
7,4′-Di-*O*-nonylnaringenin oxime (**B8**), white powder, ^1^H NMR (600 MHz, acetone-*d*_6_) δ 11.01 (s, 1H, NOH), 10.42 (s, 1H, OH-5), 7.47–7.44 (m, 2H, AA’BB’, H-2′, H-6′), 6.99–6.96 (m, 2H, AA’BB’, H-3′, H-5′), 6.05 (d, *J* = 2.4 Hz, 1H, H-6), 6.04 (d, *J* = 2.4 Hz, 1H, H-8), 5.11 (dd, *J* = 11.9, 3.2 Hz, 1H, H-2), 4.02 (t, *J* = 6.6 Hz, 2H, -CH_2_-), 3.97 (t, *J* = 6.6 Hz, 2H, -CH_2_-), 3.47 (dd, *J* = 17.1, 3.2 Hz, 1H, H-3a), 2.80 (dd, *J* = 17.1, 11.9 Hz, 1H, H-3b), 1.81–1.72 (m, 4H, 2x-CH_2_-), 1.51–1.43 (m, 4H, 2x-CH_2_-), 1.40–1.27 (m, 20H, 10x-CH_2_-), 0.91–0.85 (m, 6H, 2x-CH_3_); ^13^C NMR (150 MHz, acetone-*d*_6_) δ 162.96, 160.63, 160.23, 159.32, 154.73 (C=NOH), 132.72, 128.65, 115.27, 99.15, 96.62, 95.14, 77.22, 68.67, 68.60, 32.62, 32.61, 30.31, 30.28, 30.13, 30.07, 30.01, 30.00, 26.78, 26.71, 23.33, 14.36; HRMS (*m*/*z*): [M + H]^+^ calculated for C_33_H_50_NO_5_, 540.3684; found 540.3667.
7-*O*-Undecylnaringenin oxime (**B9**), white powder, ^1^H NMR (600 MHz, acetone-*d*_6_) δ 11.01 (s, 1H, NOH), 10.36 (s, 1H, OH-5), 8.46 (s, 1H, OH-4′), 7.40–7.36 (m, 2H, AA’BB’, H-2′, H-6′), 6.92–6.87 (m, 2H, AA’BB’, H-3′, H-5′), 6.05 (d, *J* = 2.4 Hz, 1H, H-6), 6.03 (d, *J* = 2.4 Hz, 1H, H-8), 5.07 (dd, *J* = 12.0, 3.2 Hz, 1H, H-2), 3.96 (t, *J* = 6.6 Hz, 2H, -CH_2_-), 3.46 (dd, *J* = 17.1, 3.2 Hz, 1H, H-3a), 2.79 (dd, *J* = 17.1, 12.0 Hz, 1H, H-3b), 1.78–1.70 (m, 2H, -CH_2_-), 1.48–1.41 (m, 2H, -CH_2_-), 1.41–1.26 (m, 14H, 7x-CH_2_-), 0.87 (t, *J* = 7.0 Hz, 3H, -CH_3_); ^13^C NMR (150 MHz, acetone-*d*_6_) δ 162.94, 160.62, 159.40, 158.44, 154.86 (C=NOH), 131.76, 128.77, 116.12, 99.13, 96.59, 95.11, 77.36, 68.65, 32.63, 30.33, 30.31, 30.06, 26.70, 23.32, 14.36; HRMS (*m*/*z*): [M + H]^+^ calculated for C_26_H_36_NO_5_, 442.2588; found 442.2572.
7,4′-Di-*O*-undecylnaringenin oxime (**B10**), white powder, ^1^H NMR (600 MHz, acetone-*d*_6_) δ 11.00 (s, 1H, NOH), 10.38 (s, 1H, OH-5), 7.49–7.43 (m, 2H, AA’BB’, H-2′, H-6′), 7.00–6.96 (m, 2H, AA’BB’, H-3′, H-5′), 6.05 (d, *J* = 2.4 Hz, 1H, H-6), 6.04 (d, *J* = 2.4 Hz, 1H, H-8), 5.11 (dd, *J* = 11.9, 3.2 Hz, 1H, H-2), 4.02 (t, *J* = 6.5 Hz, 2H, -CH_2_-), 3.97 (t, *J* = 6.5 Hz, 2H, -CH_2_-), 3.47 (dd, *J* = 17.1, 3.2 Hz, 1H, H-3a), 2.81 (dd, *J* = 17.1, 11.9 Hz, 1H, H-3b), 1.81–1.72 (m, 4H, 2x-CH_2_-), 1.51–1.43 (m, 4H, 2x-CH_2_-), 1.40–1.27 (m, 28H, 14x-CH_2_-), 0.90–0.85 (m, 6H, 2x-CH_3_); ^13^C NMR (150 MHz, acetone-*d*_6_) δ 162.97, 160.64, 160.24, 159.33, 154.76 (C=NOH), 132.73, 128.65, 115.28, 99.15, 96.63, 95.15, 77.23, 68.67, 68.60, 32.64, 30.35, 30.33, 30.29, 30.08, 30.02, 26.79, 26.71, 23.34, 14.37; HRMS (*m*/*z*): [M + H]^+^ calculated for C_37_H_58_NO_5_, 596.4310; found 596.4286.

### 3.2. Antimicrobial Activity

#### 3.2.1. Bacterial Strains and Culture Conditions

Non-pathogenic reference strains representing the intestinal microbiome were used for the studies: *Escherichia coli* K12, *Staphylococcus aureus* ATCC 25923 and *Enterococcus faecalis* ATCC 29212. All strains were obtained from the collection of the Department of Microbiology at the Medical University of Wrocław.

The strains were received from the museum (−80 °C) and grown in tryptic soy broth (TSB, OXOID, Basingstoke, UK) under continuous shaking conditions (MaxQTM6000 incubator shaker, Thermo Scientific, Waltham, MA, USA) at 125 rpm at 37 °C. Then, they were moved to the growth media, namely tryptic soy agar (TSA, OXOID, Basingstoke, UK) and MacConkey agar (MC, Millipore, Darmstadt, Germany) for *S. aureus* or *E. faecalis* and *E. coli*, respectively.

A fresh 18–24 h culture was used for each study. For this purpose, a culture with a density of 1.5 × 10^8^ CFU/mL in Mueller–Hinton broth (MHB, OXOID, Basingstoke, UK) was prepared and used for further microdilution studies.

#### 3.2.2. Minimal Inhibitory Concentration (MIC) and Minimal Bactericidal Concentration (MBC) Assays

The antimicrobial activity of the *O*-alkyl derivatives of naringenin and their oximes was tested in vitro using the microdilution method, showing the minimum inhibitory concentration (MIC), in accordance with the recommendations of the European Antimicrobial Susceptibility Testing Committee EUCAST [44]. MICs were determined in 96-well microdilution plates. The tests were carried out in MHB medium. The *O*-alkyl derivatives of naringenin and their oximes were tested in the concentration range of 512 to 1 µg/mL in a geometric process in MHB medium. The final concentration of the test strains in each test well was 5 × 10^5^ CFU/mL. MICs were read after 18 h (for *E. coli* and *S. aureus*) and 24 h (for *E. faecalis*) incubation at 37 °C.

A strain growth control (tested strain in medium), background control for tested compounds (naringenin derivatives in MHB), medium control (only pure MHB) and compound solvent control (DMSO at test compound concentration, strain and MHB) were set for each experiment. Each test was set up with three independent experiments, each as three separate replicates.

The optimal density, OD_600_, was measured in a plate reader (ASYS UVM340, BIOCHROM Ltd., Cambridge, UK). The background control of the tested compounds and the clean medium were included in the absorbance results. The MIC_90_ was defined as the concentration of synthesized compounds at which growth inhibition of the tested strains was observed at the level of 90% or more, compared to the control strain growth.

The minimum bactericidal concentration (MBC) was tested for all naringenin derivatives. First, 10 µL of medium was taken from the plate, on which the MIC was determined for the MIC value, the value below and the two values above. This volume was transferred to TSA or MC agar. After 18–24 h (depending on strain) incubation at 37 °C, colonies were counted. The concentration of test compounds at which no bacterial growth was observed was defined as the MBC value. Each experiment was repeated 3 times.

### 3.3. Anticancer Activity

#### 3.3.1. Cell Culture Condition

The HT-29 cells (ATCC, Manassas, VA, USA) were cultured within α-MEM (IIET PAS, Wrocław, Poland) supplemented with 10% fetal bovine serum (Sigma, St. Louis, MO, USA), antimycotic (100 μg/mL penicillin, 100 μg/mL streptomycin and 0.25 μg/mL amphotericin) (Sigma) and 2 mM glutamine (Sigma). The 3T3-L1 cells (ATCC, Manassas, VA, USA) were cultivated using DMEM high-glucose medium (IIET PAS, Wrocław, Poland) supplemented as above. Both cell lines were cultured at 37 °C in an atmosphere of 5% CO_2_.

#### 3.3.2. SRB Assay

The sulforhodamine B (SRB) assay was performed according to a previously published protocol [21]. Briefly, 1 × 10^4^ HT-29 or 5 × 10^3^ 3T3-L1 cells were seeded into 96-well plates. After attaching to the plate surface, the cells were incubated with 100, 75, 50, 25, 10 or 1 µg/mL of the investigated compounds for 24 h. After this time, proteins were precipitated with trichloroacetic acid and stained with SRB. The absorbance was measured at 560 nm using a GloMax Discover Microplate Reader (Promega, Madison, WI, USA).

#### 3.3.3. MTT Assay

First, 1 × 10^4^ HT-29 cells or 5 × 10^3^ 3T3-L1 cells were seeded into a 96-well plate and incubated for one night. Then, cells were treated with different concentrations of the investigated compounds within 24 h. Afterwards, the medium was replaced with a fresh medium containing 0.5 mg/mL of 3-(4,5-dimethylthiazol-2-yl)-2,5-diphenyl-2H-tetrazolium bromide (MTT, Sigma) and incubated for 2 h at 37 °C. After removing the medium, the formazan salts were dissolved in dimethyl sulfoxide (DMSO, Sigma), and the absorbance of the solution was measured at 560 nm using a plate reader.

#### 3.3.4. Apoptosis Assay

The process of apoptosis was determined using the RealTime-Glo Annexin V Apoptosis and Necrosis Assay (Promega) according to the manufacturer’s protocol. Briefly, 1 × 10^4^ HT-29 cells were seeded into a single well of a 96-well plate. After overnight incubation, the cells were treated with 10, 25, 50 and 100 µg/mL of the investigated compounds and detection reagent for 24 h. As a negative control, untreated cells were used. The luminescence, fluorescence and OD_525_ signals were detected at each hour of the first 12 h of incubation. The end measurement was performed at the 24th hour of the experiment. All samples were prepared in triplicate, and then their means were calculated.

#### 3.3.5. Caspase 3/7 Activity Assay

The activity of two effector caspases 3 and 7 was analyzed using the Caspase-Glo 3/7 Assay (Promega) according to the recommendations of the manufacturer. The cells were seeded at a density of 1 × 10^4^ per well for the test on a 96-well plate. Then, cells were incubated with **A2**, **B2**, **A9**, **B9** and **NG** at a concentration of 50 µg/mL for 48 h. Half an hour before each measurement of caspase activity, an equal volume of Caspase-Glo 3/7 substrate solution was added to cells. The luminescence signals were detected using a GloMax Discover Microplate Reader at the 6th, 12th, 24th and 48th hours of the experiment. The reactions for each sample were repeated in triplicate and presented as the mean value.

## 4. Conclusions

In this paper, we described the synthesis of new ether derivatives of naringenin (**A3**, **A4**, **A6**, **A8**–**A10**) and their oximes (**B3**–**B10**). We also evaluated the influence of 20 *O*-alkyl derivatives and oximes against representatives of the human gut microbiota, *S. aureus*, *E. faecalis* and *E. coli*, and the cytotoxic effects against the HT-29 and 3T3-L1 cell lines.

Among the synthesized derivatives, none of them inhibited the growth of *E. coli*. In the case of *E. faecalis* and *S. aureus*, only oximes of di-*O*-alkyl derivatives were safe, with MIC > 512 µg/mL. Furthermore, 7-*O*-undecylnaringenin (**A9**) and its oxime (**B9**) exhibited no impact on the multiplication of both Gram-positive strains. We have also demonstrated that the greatest antiproliferative activity was exhibited by 7-*O*-alkyl derivatives **A1**, **A3**, **A5**, **A7** and **A9**. The introduction of an oxime moiety resulted in stronger activity (IC_50_ for **B1**, **B3**, **B5**, **B7** and **B9** in the range of 9.96–11.42 µg/mL). Among the di-*O*-alkyl derivatives and their oximes, only the incorporation of the NOH group into 7,4′-di-*O*-hexylnaringenin resulted in a two-times stronger cytotoxic effect (IC_50_ for **A2** > 100 µg/mL; IC_50_ for **B2** was 49.76 ± 1.63 µg/mL). To determine the mechanism of action, we selected four compounds (**A2**, **B2**, **A9** and **B9**) that were safe for representatives of the human gut microbiota and characterized them in terms of their antiproliferative activity against the HT-29 cell line. Among them, the most effective was 7-*O*-undecylnaringenin oxime (**B9**), which increased the level of phosphatidylserine and caused a loss of membrane integrity. In addition, it induced the rapid activation of caspase 3/7 at a concentration of 50 µg/mL. These results show that this is the most promising anticancer agent, safe for representatives of the human intestinal microflora.

## Figures and Tables

**Table 1:** Yields (%) and melting points (m.p., [°C]) of synthesized naringenin derivatives.

Compound	Yield	m.p.	Compound	Yield	m.p.
**A3**	71.5%	114–115 °C	**B3**	90.3%	186–187 °C
**A4**	10.0%	74–75 °C	**B4**	78.4%	82–83 °C
**A5**	66.4%	111–113 °C	**B5**	94.3%	183–185 °C
**A6**	10.8%	60–61 °C	**B6**	72.2%	85–86 °C
**A7**	64.7%	115–116 °C	**B7**	93.2%	176–178 °C
**A8**	11.2%	70–71 °C	**B8**	88.0%	87–88 °C
**A9**	63.2%	103–104 °C	**B9**	91.6%	171–173 °C
**A10**	9.9%	63–64 °C	**B10**	77.5%	95–96 °C

**Table 2:** Minimum inhibitory concentrations (MIC) and minimal bactericidal concentrations (MBC) of *O*-alkyl derivatives of naringenin (**A1**–**A10**) and their oximes (**B1**–**B10**).

Compound	MIC (MBC) [µg/mL]		
	*S. aureus* ATCC 25923	*E. faecalis* ATCC 29212	*E. coli* K12
**A1**	16 (>512)	16 (>512)	>512 (>512)
**A2**	>512 (>512)	64 (>512)	>512 (>512)
**A3**	16 (>512)	32 (>512)	>512 (>512)
**A4**	>512 (>512)	32 (>512)	>512 (>512)
**A5**	8 (>512)	16 (>512)	>512 (>512)
**A6**	>512 (>512)	32 (>512)	>512 (>512)
**A7**	16 (>512)	16 (>512)	>512 (>512)
**A8**	512 (>512)	512 (>512)	>512 (>512)
**A9**	>512 (>512)	512 (>512)	>512 (>512)
**A10**	256 (>512)	32 (>512)	>512 (>512)
**NG**	512 (>512)	>512 (>512)	512 (>512)
**B1**	8 (32)	16 (256)	>512 (>512)
**B2**	>512 (>512)	>512 (>512)	>512 (>512)
**B3**	64 (128)	16 (512)	>512 (>512)
**B4**	>512 (>512)	>512 (>512)	>512 (>512)
**B5**	256 (>512)	16 (256)	>512 (>512)
**B6**	>512 (>512)	>512 (>512)	>512 (>512)
**B7**	128 (>512)	8 (>512)	>512 (>512)
**B8**	>512 (>512)	>512 (>512)	>512 (>512)
**B9**	>512 (>512)	256 (>512)	>512 (>512)
**B10**	>512 (>512)	>512 (>512)	>512 (>512)
**NGOX**	>512 (>512)	512 (>512)	512 (>512)

**Table 3:** Cytotoxicity of naringenin derivatives in HT-29 and 3T3-L1 cells, presented as the mean IC_50_. IC_50_ means were calculated from three independent repeats of SRB tests after 24 h exposure to naringenin derivatives.

Compound	Cytotoxicity (IC_50_ [µg/mL] ± SD)	
	HT-29	3T3-L1
**A1**	14.31 ± 0.84	10.83 ± 1.10
**A2**	>100	>100
**A3**	14.19 ± 0.56	7.61 ± 1.20
**A4**	>100	>100
**A5**	14.63 ± 0.13	8.86 ± 0.67
**A6**	>100	>100
**A7**	15.02 ± 0.41	8.65 ± 0.71
**A8**	>100	>100
**A9**	17.85 ± 0.65	10.17 ± 0.20
**A10**	>100	>100
**NG**	>100	>100
**B1**	11.32 ± 1.63	8.30 ± 0.72
**B2**	49.76 ± 1.63	10.08 ± 6.49
**B3**	11.30 ± 0.81	7.77 ± 1.11
**B4**	>100	>100
**B5**	9.96 ± 1.13	7.1 ± 1.57
**B6**	>100	>100
**B7**	11.23 ± 1.27	6.08 ± 0.64
**B8**	>100	>100
**B9**	11.42 ± 1.17	6.74 ± 0.84
**B10**	>100	>100
**NGOX**	>100	29.18 ± 2.00

## Data Availability

Samples of the compounds **NG**, **NGOX**, **A1**–**A10** and **B1**–**B10** are available from the authors.

## Supplementary Materials

The following supporting information can be downloaded at: https://www.mdpi.com/article/10.3390/ijms24129856/s1.
