# Supplementary material for: Antiproliferative Activity and Impact on Human Gut Microbiota of New O-Alkyl Derivatives of Naringenin and Their Oximes
Source: Int J Mol Sci. 2023 Jun 7;24(12):9856. doi: 10.3390/ijms24129856 (PMC10298275; doi:10.3390/ijms24129856)
Supplement: Supplementary file 1 [file ijms-24-09856-s001.zip › ijms-2427741-supplementary.pdf]

Antiproliferative Activity and Impact on Human Gut Microbiota of New *O*-Alkyl Derivatives of Naringenin and Their Oximes

Joanna Kozłowska [1,*], Anna Duda-Madej [2] and Dagmara Baczyńska [3]

Figure S1. ^{1}H NMR (600 MHz, chloroform-*d*) spectrum of 7-*O*-heptylnaringenin (**A3**)

Figure S2. ^{13}C NMR (150 MHz, chloroform-*d*) spectrum of 7-*O*-heptylnaringenin (**A3**)

Figure S3. COSY NMR (150 MHz, chloroform-*d*) spectrum of 7-*O*-heptylnaringenin (**A3**)

Figure S4. HSQC NMR (150 MHz, chloroform-*d*) spectrum of 7-*O*-heptylnaringenin (**A3**)

Figure S5. ^{1}H NMR (600 MHz, chloroform-*d*) spectrum of 7,4′-di-*O*-heptylnaringenin (**A4**)

Figure S6. ^{13}C NMR (150 MHz, chloroform-*d*) spectrum of 7,4′-di-*O*-heptylnaringenin (**A4**)

Figure S7. COSY NMR (150 MHz, chloroform-*d*) spectrum of 7,4′-di-*O*-heptylnaringenin (**A4**)

Figure S8. HSQC NMR (150 MHz, chloroform-*d*) spectrum of 7,4′-di-*O*-heptylnaringenin (**A4**)

Figure S9. ^{1}H NMR (600 MHz, chloroform-*d*) spectrum of 7-*O*-octylnaringenin (**A5**)

Figure S10. ^{13}C NMR (150 MHz, chloroform-*d*) spectrum of 7-*O*-octylnaringenin (**A5**)

Figure S11. COSY NMR (150 MHz, chloroform-*d*) spectrum of 7-*O*-octylnaringenin (**A5**)

Figure S12. HSQC NMR (150 MHz, chloroform-*d*) spectrum of 7-*O*-octylnaringenin (**A5**)

Figure S13. ^{1}H NMR (600 MHz, chloroform-*d*) spectrum of 7,4′-di-*O*-octylnaringenin (**A6**)

Figure S14. ^{13}C NMR (150 MHz, chloroform-*d*) spectrum of 7,4′-di-*O*-octylnaringenin (**A6**)

Figure S15. COSY NMR (150 MHz, chloroform-*d*) spectrum of 7,4′-di-*O*-octylnaringenin (**A6**)

Figure S16. HSQC NMR (150 MHz, chloroform-*d*) spectrum of 7,4′-di-*O*-octylnaringenin (**A6**)

Figure S17. ^{1}H NMR (600 MHz, chloroform-*d*) spectrum of 7-*O*-nonylnaringenin (**A7**)

Figure S18. ^{13}C NMR (150 MHz, chloroform-*d*) spectrum of 7-*O*-nonylnaringenin (**A7**)

Figure S19. COSY NMR (150 MHz, chloroform-*d*) spectrum of 7-*O*-nonylnaringenin (**A7**)

Figure S20. HSQC NMR (150 MHz, chloroform-*d*) spectrum of 7-*O*-nonylnaringenin (**A7**)

Figure S21. ^{1}H NMR (600 MHz, chloroform-*d*) spectrum of 7,4′-di-*O*-nonylnaringenin (**A8**)

Figure S22. ^{13}C NMR (150 MHz, chloroform-*d*) spectrum of 7,4′-di-*O*-nonylnaringenin (**A8**)

Figure S23. COSY NMR (150 MHz, chloroform-*d*) spectrum of 7,4′-di-*O*-nonylnaringenin (**A8**)

Figure S24. HSQC NMR (150 MHz, chloroform-*d*) spectrum of 7,4′-di-*O*-nonylnaringenin (**A8**)

Figure S25. ^{1}H NMR (600 MHz, chloroform-*d*) spectrum of 7-*O*-undecylnaringenin (**A9**)

Figure S26. ^{13}C NMR (150 MHz, chloroform-*d*) spectrum of 7-*O*-undecylnaringenin (**A9**)

Figure S27. COSY NMR (150 MHz, chloroform-*d*) spectrum of 7-*O*-undecylnaringenin (**A9**)

Figure S28. HSQC NMR (150 MHz, chloroform-*d*) spectrum of 7-*O*-undecylnaringenin (**A9**)

Figure S29. ^{1}H NMR (600 MHz, chloroform-*d*) spectrum of 7,4′-di-*O*-undecylnaringenin (**A10**)

Figure S30. ^{13}C NMR (150 MHz, chloroform-*d*) spectrum of 7,4′-di-*O*-undecylnaringenin (**A10**)

Figure S31. COSY NMR (150 MHz, chloroform-*d*) spectrum of 7,4′-di-*O*-undecylnaringenin (**A10**)

Figure S32. HSQC NMR (150 MHz, chloroform-*d*) spectrum of 7,4′-di-*O*-undecylnaringenin (**A10**)

Figure S33. ^{1}H NMR (600 MHz, acetone-d_6) spectrum of 7-*O*-heptylnaringenin oxime (**B3**)

Figure S34. ^{13}C NMR (150 MHz, acetone-d_6) spectrum of 7-*O*-heptylnaringenin oxime (**B3**)

Figure S35. COSY NMR (150 MHz, acetone-d_6) spectrum of 7-*O*-heptylnaringenin oxime (**B3**)

Figure S36. HSQC NMR (150 MHz, acetone-d_6) spectrum of 7-*O*-heptylnaringenin oxime (**B3**)

Figure S37. ^{1}H NMR (600 MHz, acetone-d_6) spectrum of 7,4′-di-*O*-heptylnaringenin oxime (**B4**)

Figure S38. ^{13}C NMR (150 MHz, acetone-d_6) spectrum of 7,4′-di-*O*-heptylnaringenin oxime (**B4**)

Figure S39. COSY NMR (150 MHz, acetone-d_6) spectrum of 7,4′-di-*O*-heptylnaringenin oxime (**B4**)

Figure S40. HSQC NMR (150 MHz, acetone-d_6) spectrum of 7,4′-di-*O*-heptylnaringenin oxime (**B4**)

Figure S41. ^{1}H NMR (600 MHz, acetone-d_6) spectrum of 7-*O*-octylnaringenin oxime (**B5**)

Figure S42. ^{13}C NMR (150 MHz, acetone-d_6) spectrum of 7-*O*-octylnaringenin oxime (**B5**)

Figure S43. COSY NMR (150 MHz, acetone-d_6) spectrum of 7-*O*-octylnaringenin oxime (**B5**)

Figure S44. HSQC NMR (150 MHz, acetone-d_6) spectrum of 7-*O*-octylnaringenin oxime (**B5**)

Figure S45. ^{1}H NMR (600 MHz, acetone-d_6) spectrum of 7,4′-di-*O*-octylnaringenin oxime (**B6**)

Figure S46. ^{13}C NMR (150 MHz, acetone-d_6) spectrum of 7,4′-di-*O*-octylnaringenin oxime (**B6**)

Figure S47. COSY NMR (150 MHz, acetone-d_6) spectrum of 7,4′-di-*O*-octylnaringenin oxime (**B6**)

Figure S48. HSQC NMR (150 MHz, acetone-d_6) spectrum of 7,4′-di-*O*-octylnaringenin oxime (**B6**)

Figure S49. ^{1}H NMR (600 MHz, acetone-d_6) spectrum of 7-*O*-nonylnaringenin oxime (**B7**)

Figure S50. ^{13}C NMR (150 MHz, acetone-d_6) spectrum of 7-*O*-nonylnaringenin oxime (**B7**)

Figure S51. COSY NMR (150 MHz, acetone-d_6) spectrum of 7-*O*-nonylnaringenin oxime (**B7**)

Figure S52. HSQC NMR (150 MHz, acetone-d_6) spectrum of 7-*O*-nonylnaringenin oxime (**B7**)

Figure S53. ^{1}H NMR (600 MHz, acetone-d_6) spectrum of 7,4′-di-*O*-nonylnaringenin oxime (**B8**)

Figure S54. ^{13}C NMR (150 MHz, acetone-d_6) spectrum of 7,4′-di-*O*-nonylnaringenin oxime (**B8**)

Figure S55. COSY NMR (150 MHz, acetone-d_6) spectrum of 7,4′-di-*O*-nonylnaringenin oxime (**B8**)

Figure S56. HSQC NMR (150 MHz, acetone-d_6) spectrum of 7,4′-di-*O*-nonylnaringenin oxime (**B8**)

Figure S57. ^{1}H NMR (600 MHz, acetone-d_6) spectrum of 7-*O*-undecylnaringenin oxime (**B9**)

Figure S58. ^{13}C NMR (150 MHz, acetone-*d*$_6$) spectrum of 7-*O*-undecylnaringenin oxime (**B9**)

Figure S59. COSY NMR (150 MHz, acetone-*d*$_6$) spectrum of 7-*O*-undecylnaringenin oxime (**B9**)

Figure S60. HSQC NMR (150 MHz, acetone-*d*$_6$) spectrum of 7-*O*-undecylnaringenin oxime (**B9**)

Figure S61. ^{1}H NMR (600 MHz, acetone-*d*$_6$) spectrum of 7,4′-di-*O*-undecylnaringenin oxime (**B10**)

Figure S62. ^{13}C NMR (150 MHz, acetone-*d*$_6$) spectrum of 7,4′-di-*O*-undecylnaringenin oxime (**B10**)

Figure S63. COSY NMR (150 MHz, acetone-*d*$_6$) spectrum of 7,4′-di-*O*-undecylnaringenin oxime (**B10**)

Figure S64. HSQC NMR (150 MHz, acetone-*d*$_6$) spectrum of 7,4′-di-*O*-undecylnaringenin oxime (**B10**)

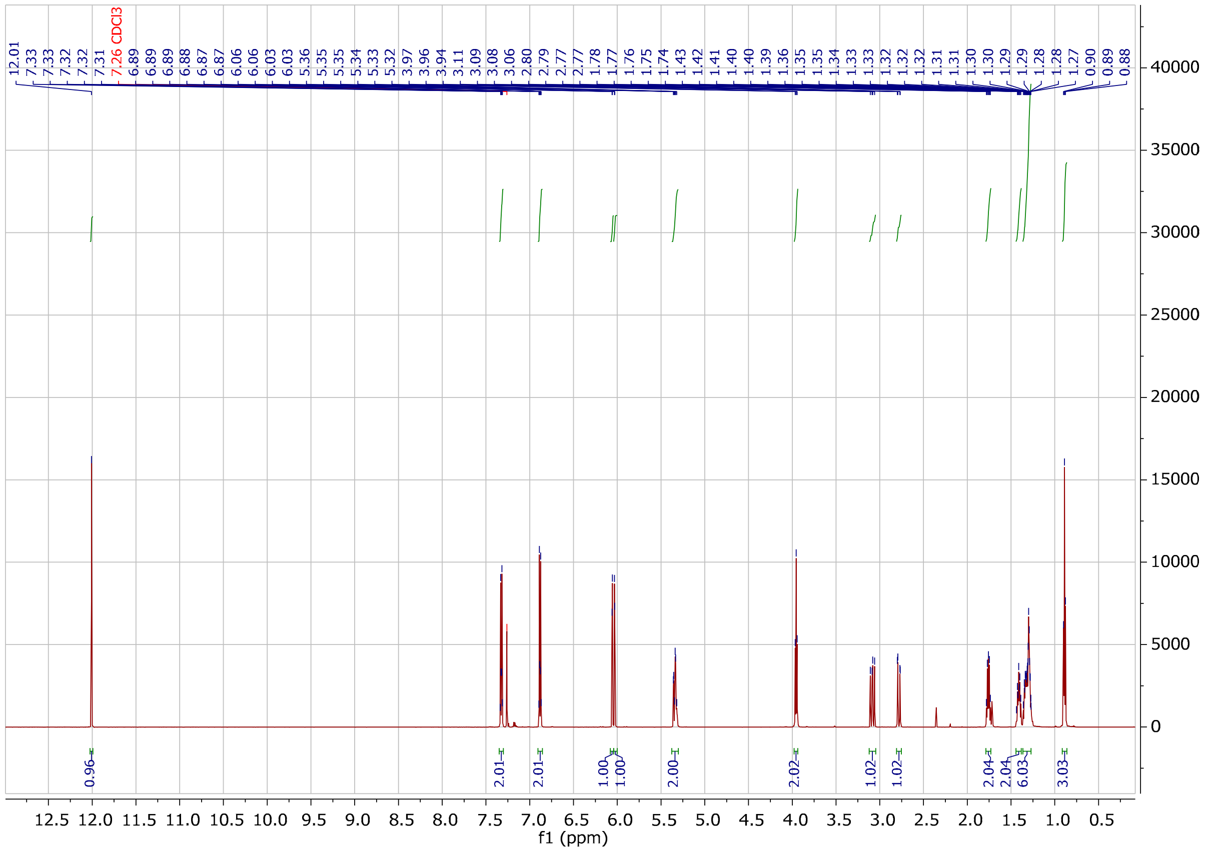

Figure S1. ^{1}H NMR (600 MHz, chloroform-*d*) spectrum of 7-*O*-heptylnaringenin (**A3**)

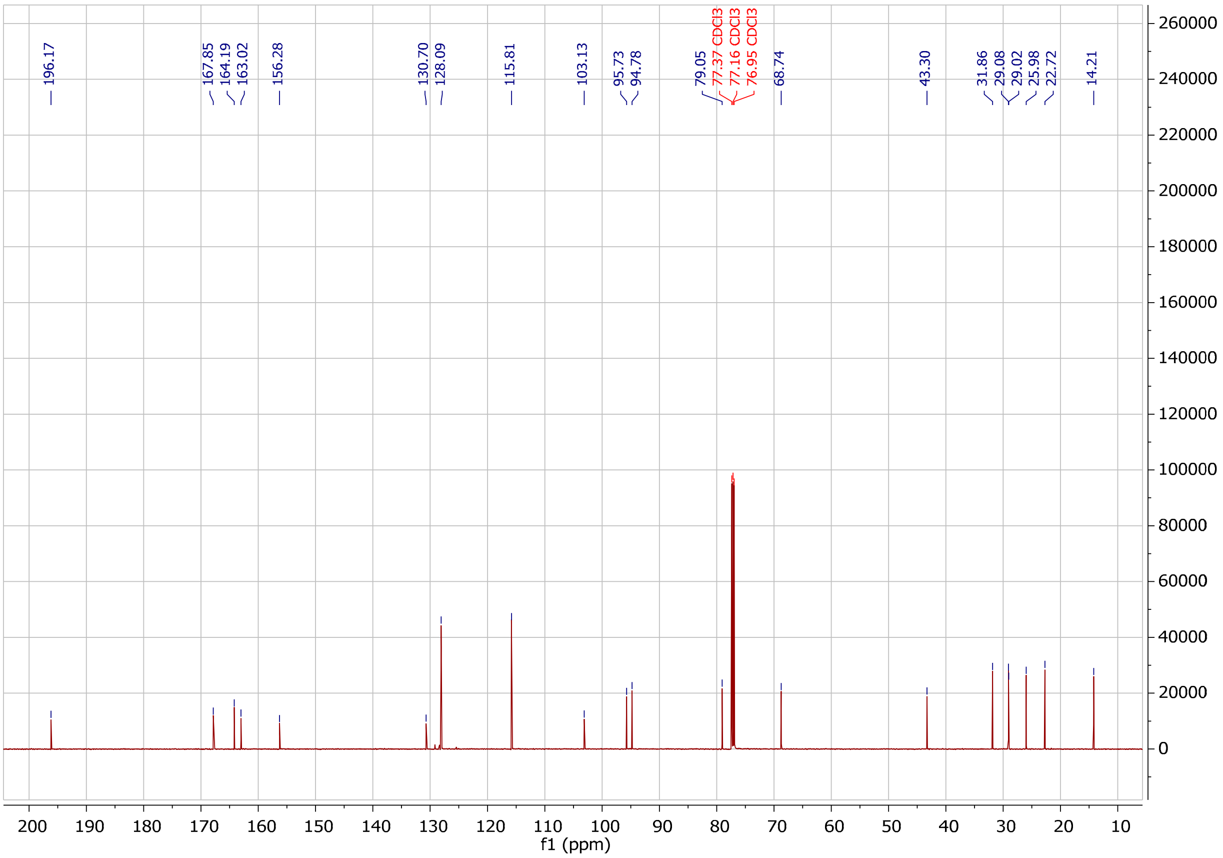

Figure S2. ^{13}C NMR (150 MHz, chloroform-*d*) spectrum of 7-*O*-heptylnaringenin (**A3**)

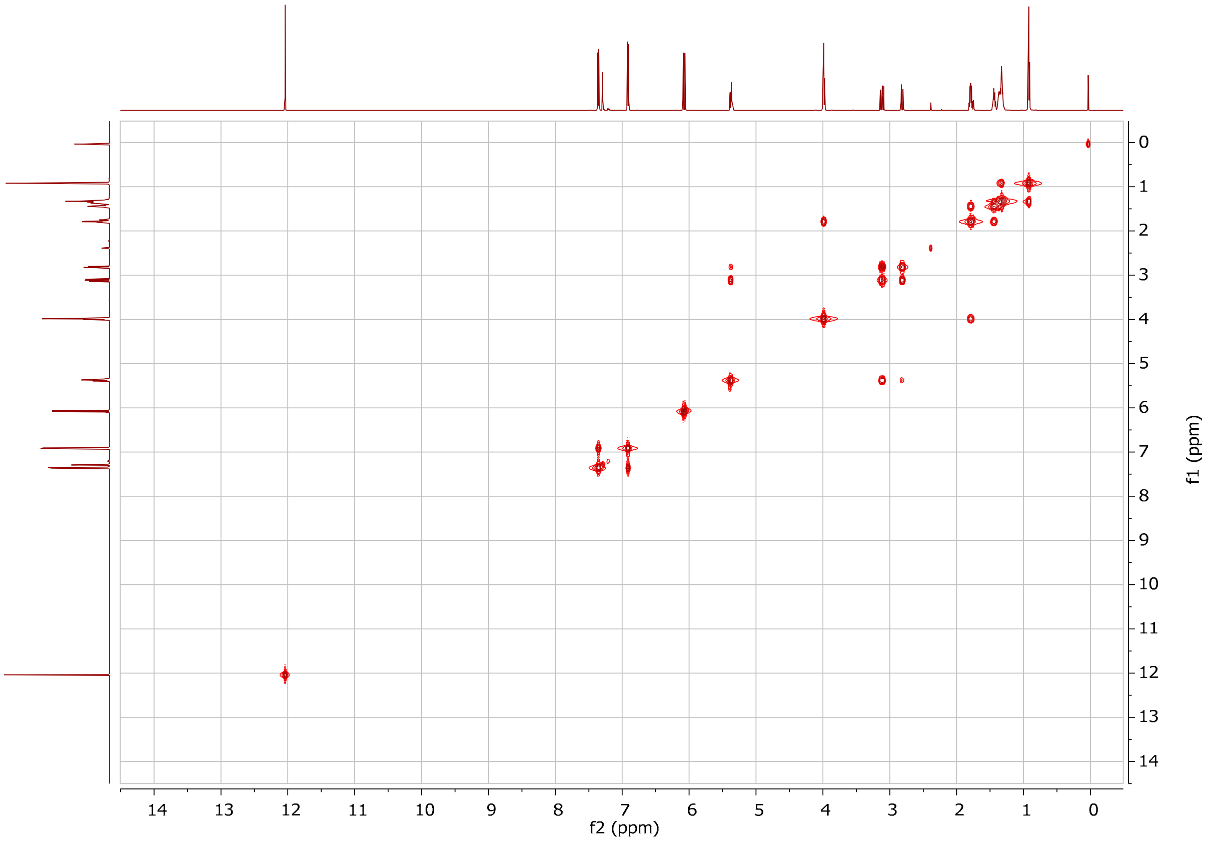

Figure S3. COSY NMR (150 MHz, chloroform-*d*) spectrum of 7-*O*-heptylnaringenin (**A3**)

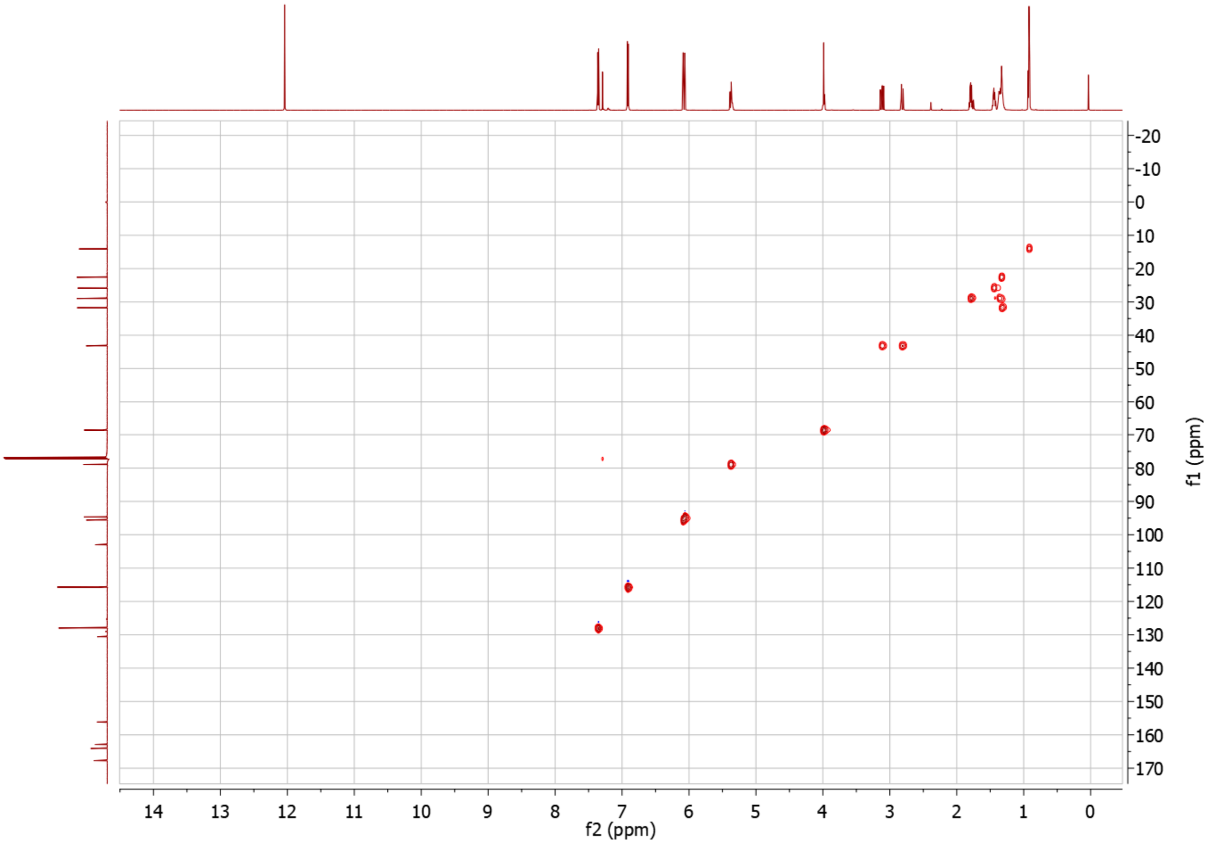

Figure S4. HSQC NMR (150 MHz, chloroform-*d*) spectrum of 7-*O*-heptylnaringenin (**A3**)

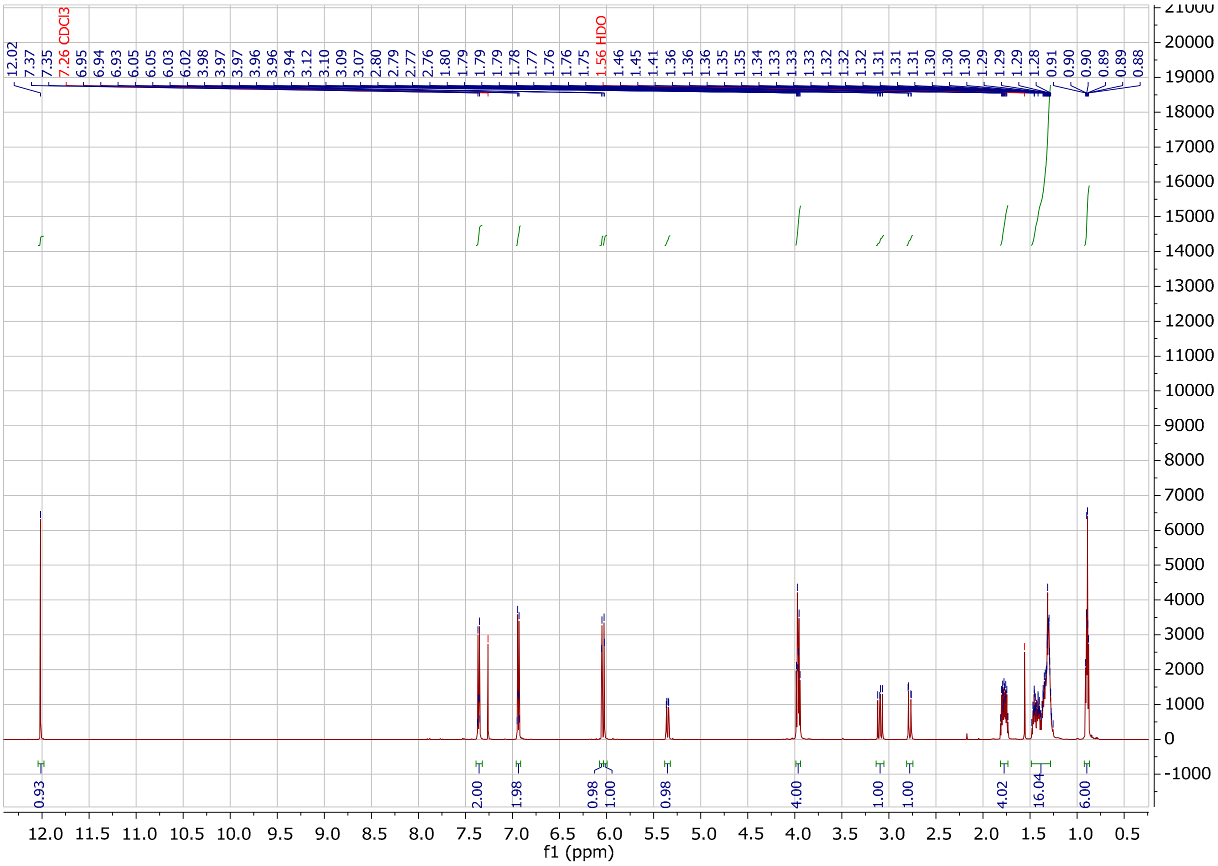

Figure S5. ¹H NMR (600 MHz, chloroform-*d*) spectrum of 7,4′-di-*O*-heptylnaringenin (**A4**)

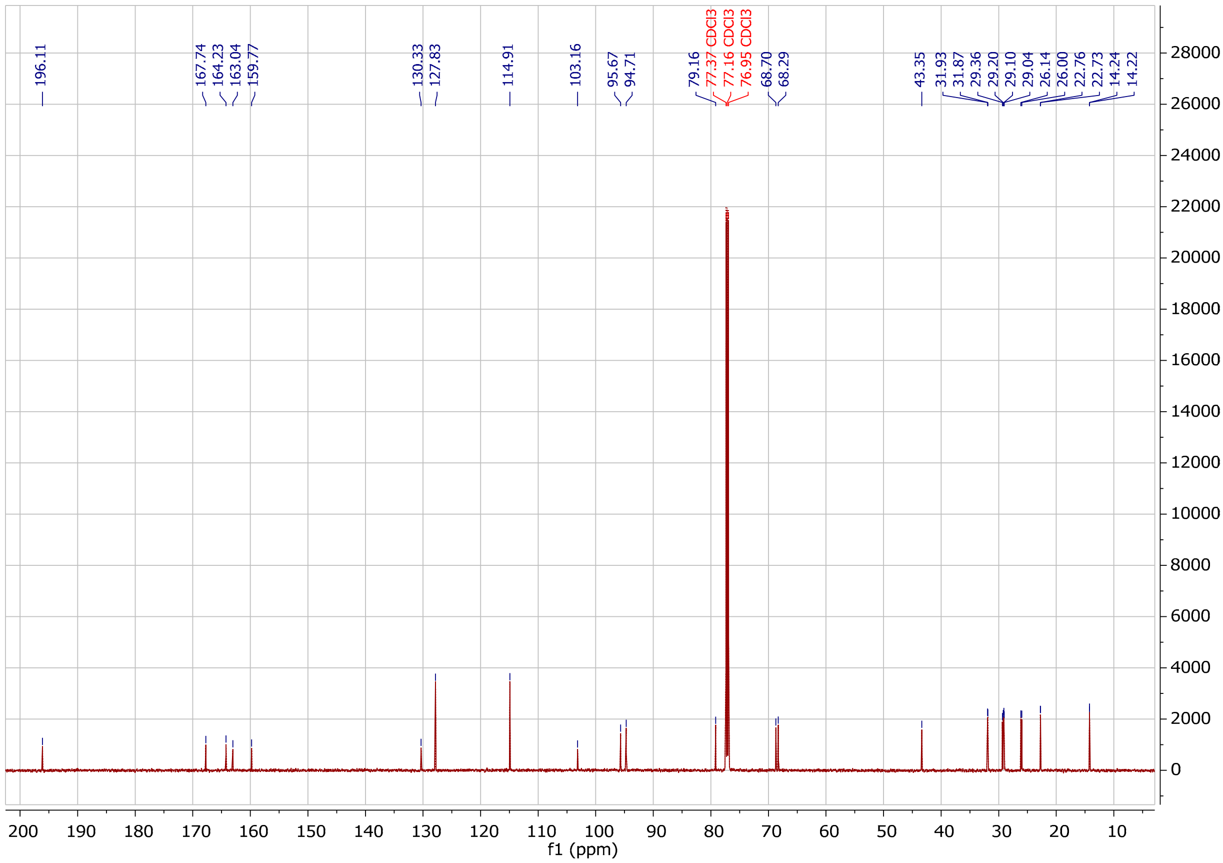

Figure S6. ^{13}C NMR (150 MHz, chloroform-*d*) spectrum of 7,4′-di-*O*-heptylnaringenin (**A4**)

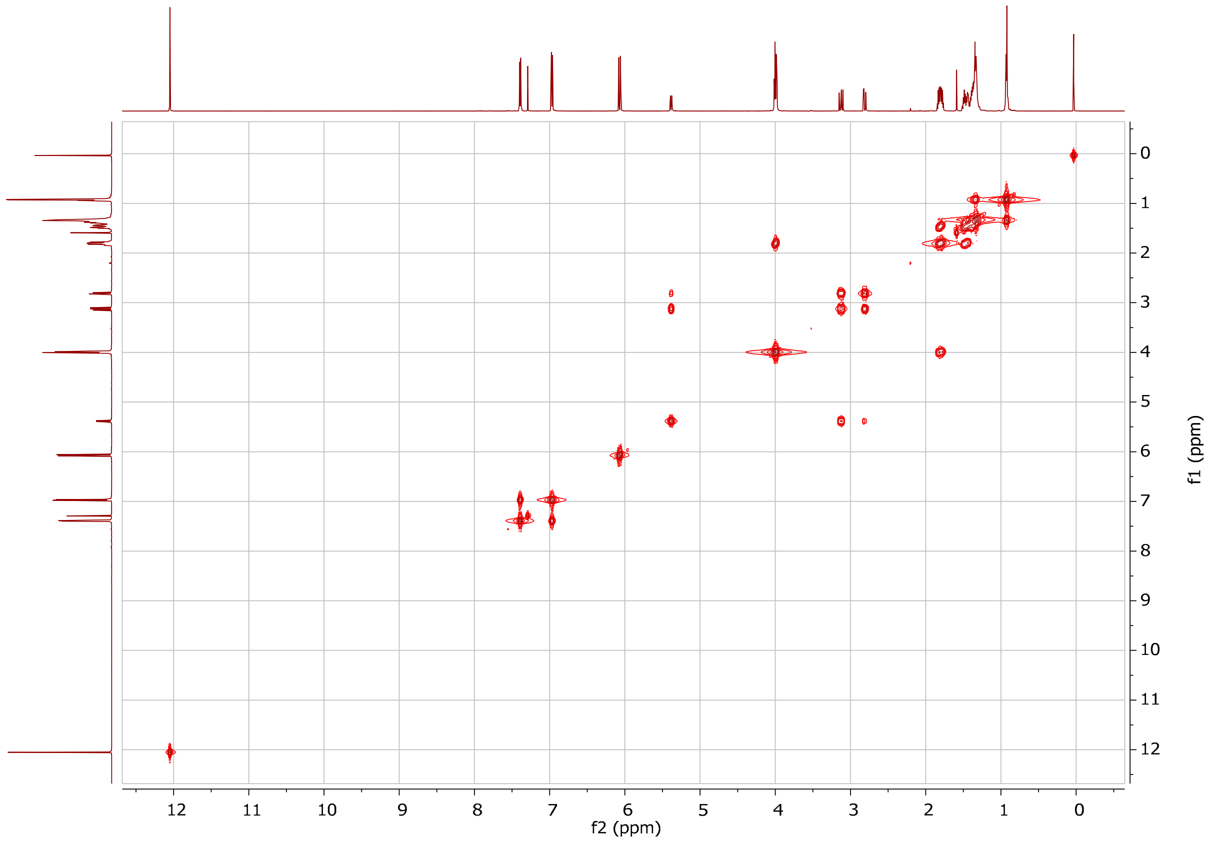

Figure S7. COSY NMR (150 MHz, chloroform-*d*) spectrum of 7,4′-di-*O*-heptylnaringenin (**A4**)

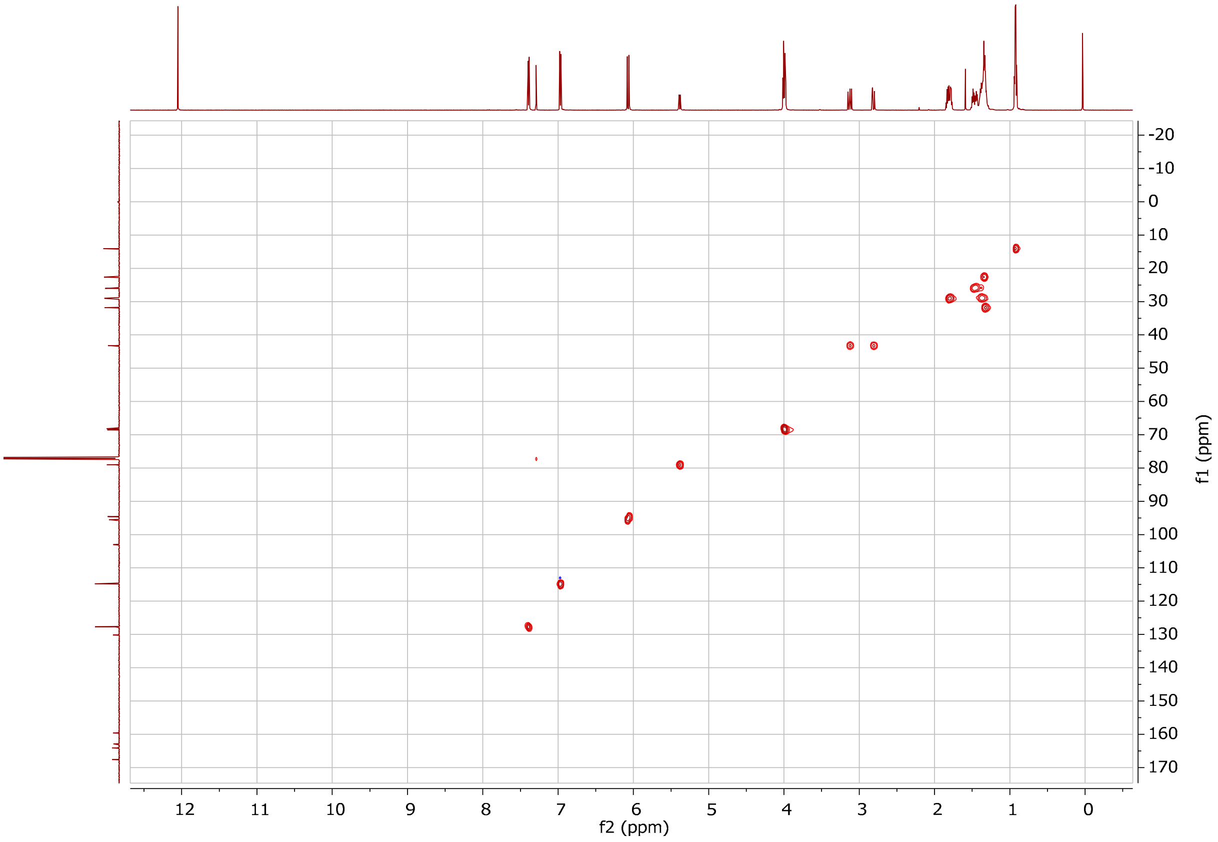

Figure S8. HSQC NMR (150 MHz, chloroform-*d*) spectrum of 7,4′-di-*O*-heptylnaringenin (**A4**)

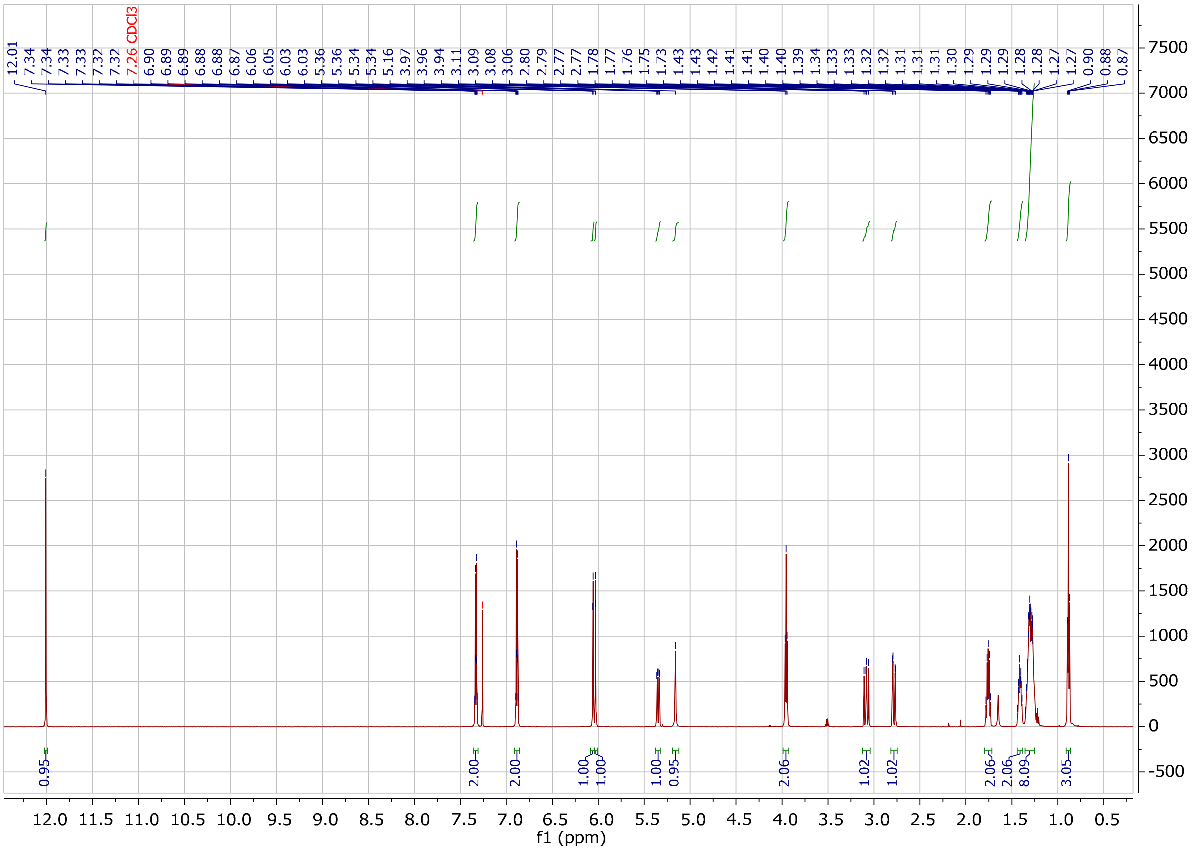

Figure S9. ^{1}H NMR (600 MHz, chloroform-*d*) spectrum of 7-*O*-octylnaringenin (**A5**)

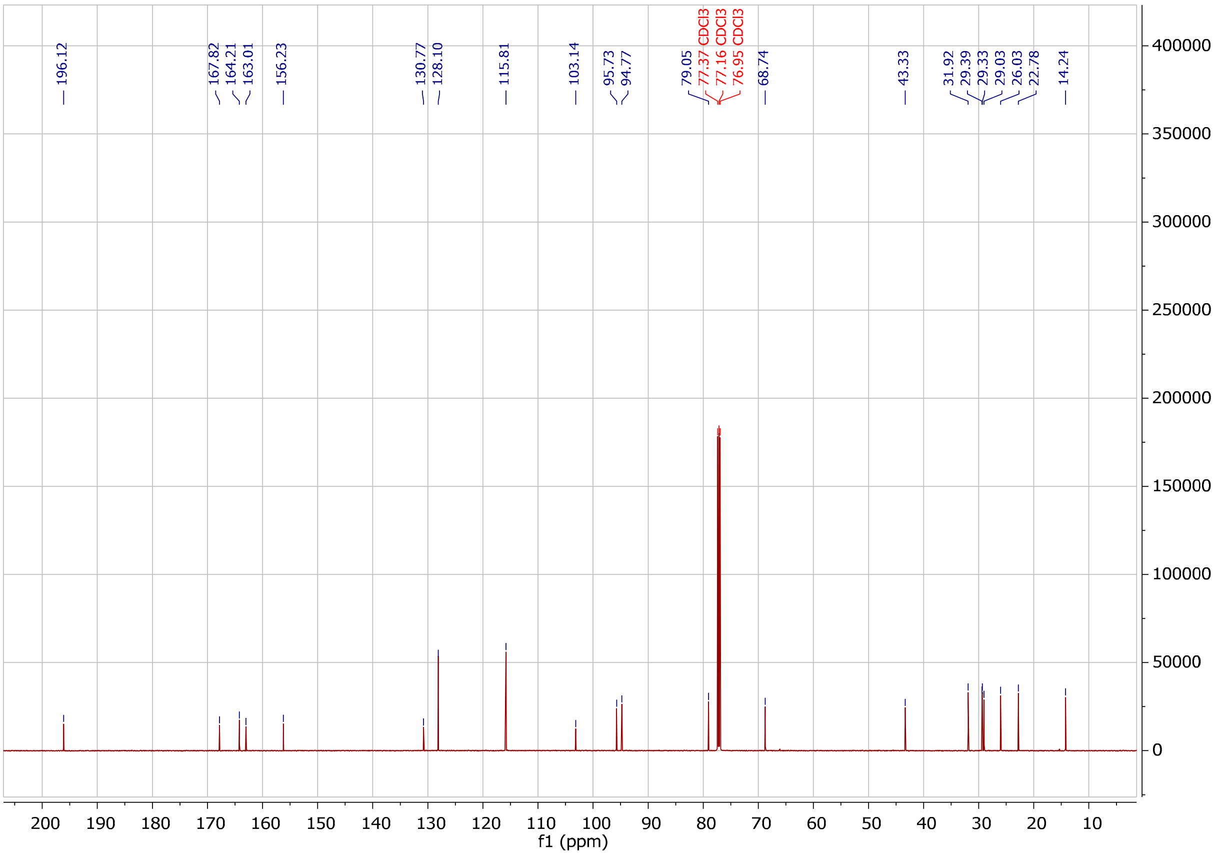

Figure S10. ^{13}C NMR (150 MHz, chloroform-*d*) spectrum of 7-*O*-octylnaringenin (**A5**)

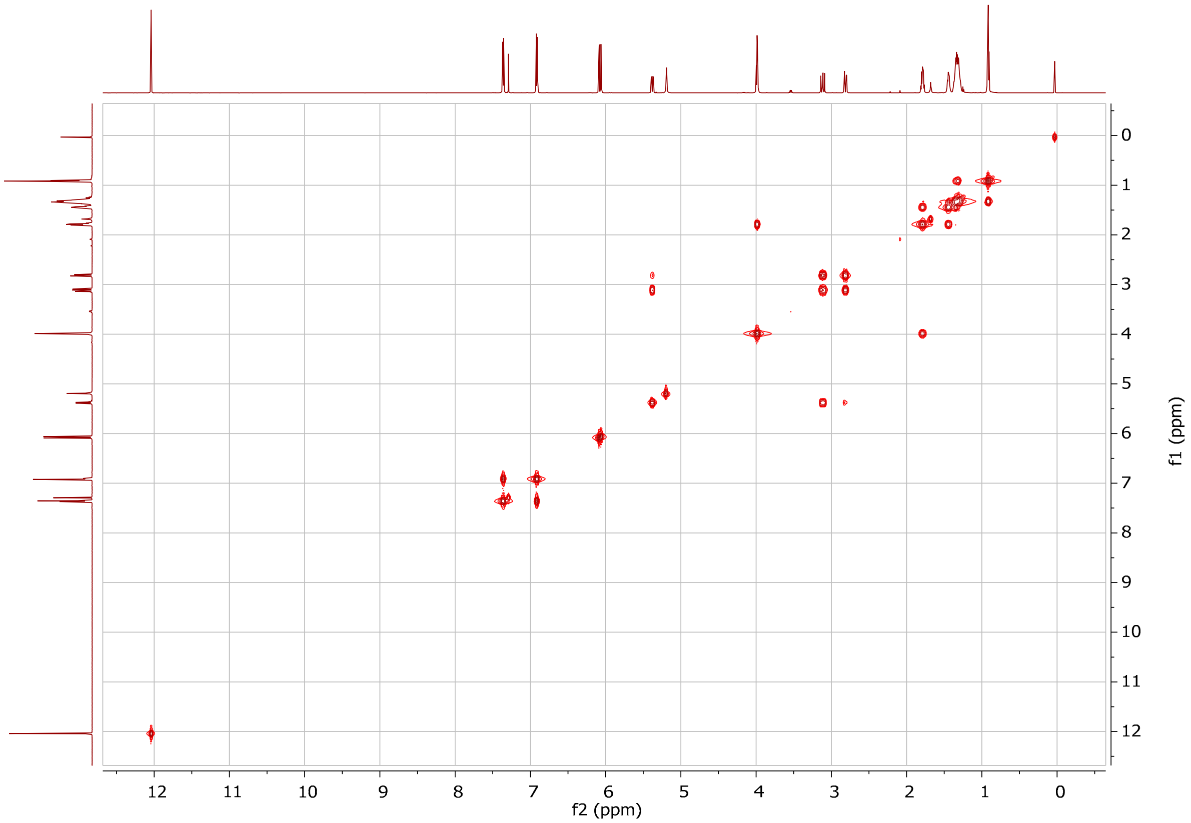

Figure S11. COSY NMR (150 MHz, chloroform-*d*) spectrum of 7-*O*-octylnaringenin (**A5**)

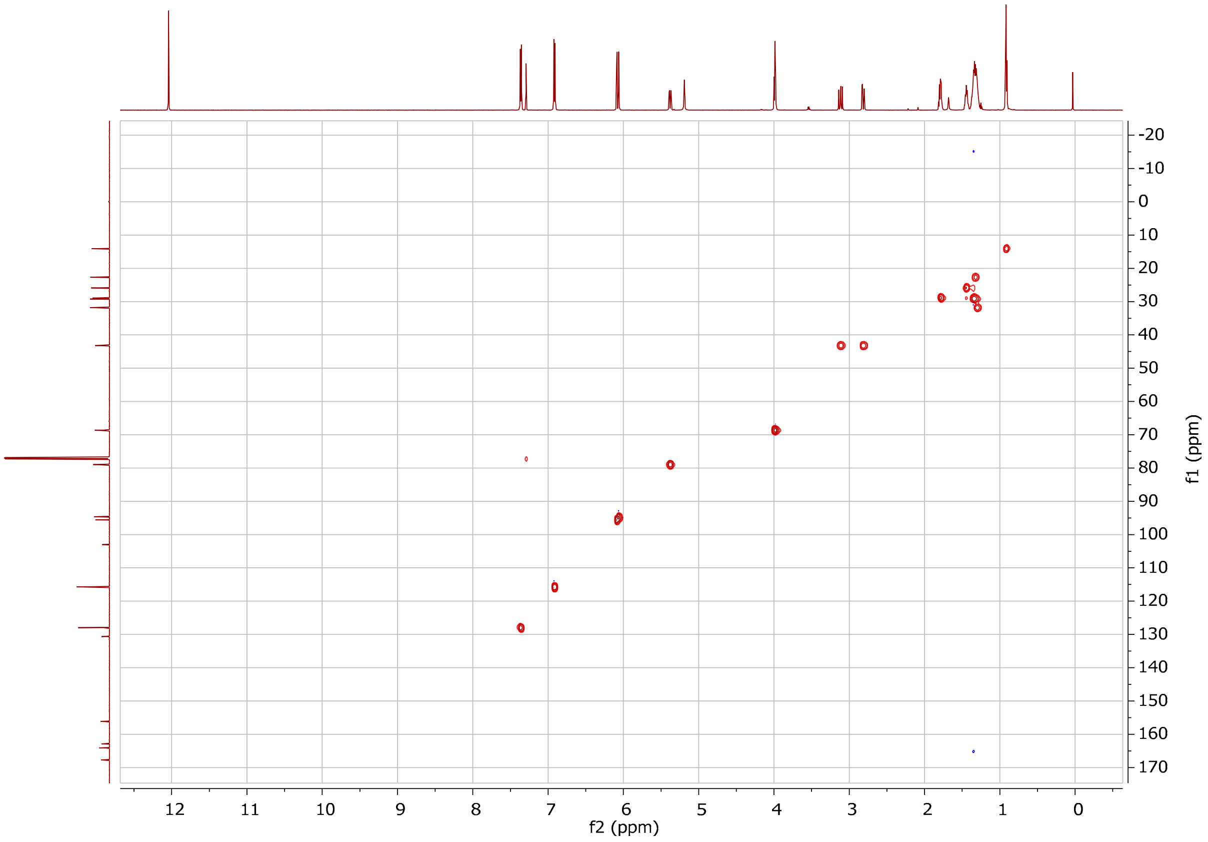

Figure S12. HSQC NMR (150 MHz, chloroform-*d*) spectrum of 7-*O*-octylnaringenin (**A5**)

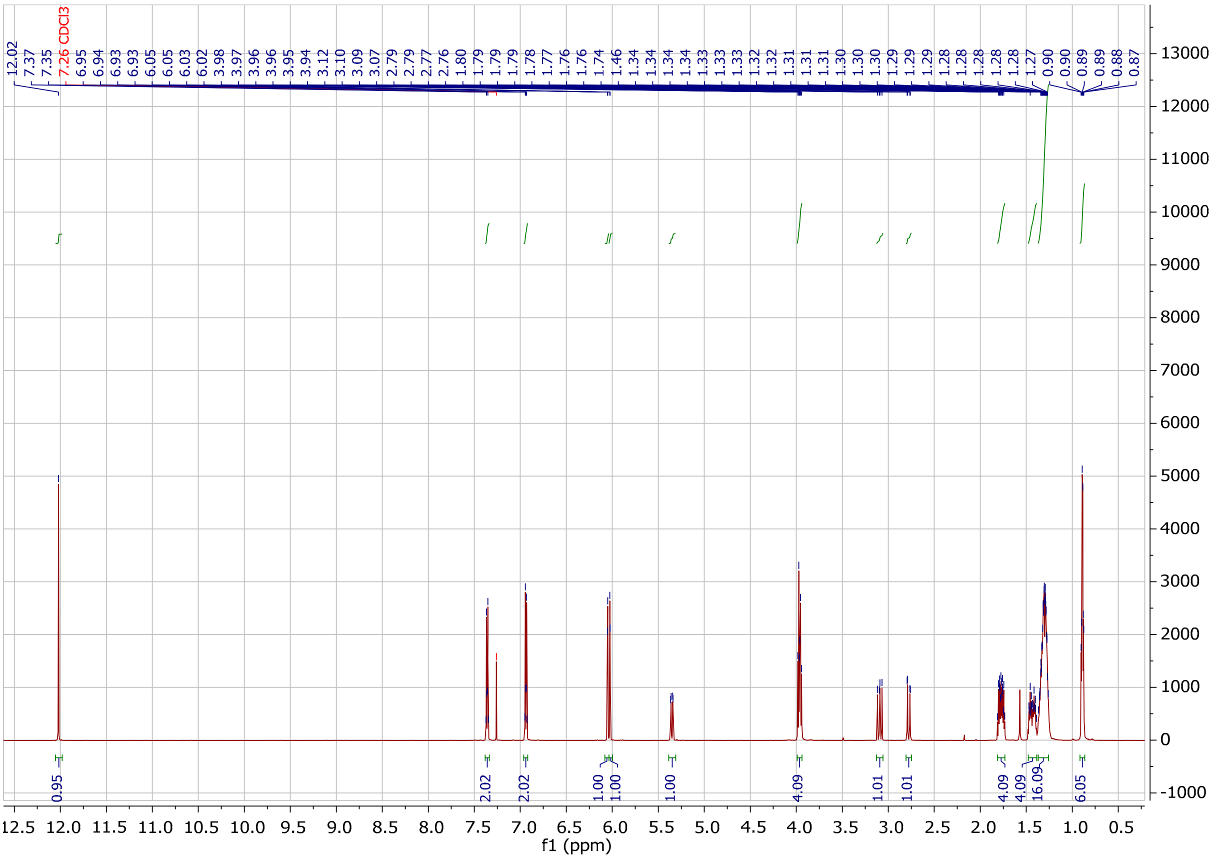

Figure S13. ¹H NMR (600 MHz, chloroform-*d*) spectrum of 7,4′-di-*O*-octylnaringenin (**A6**)

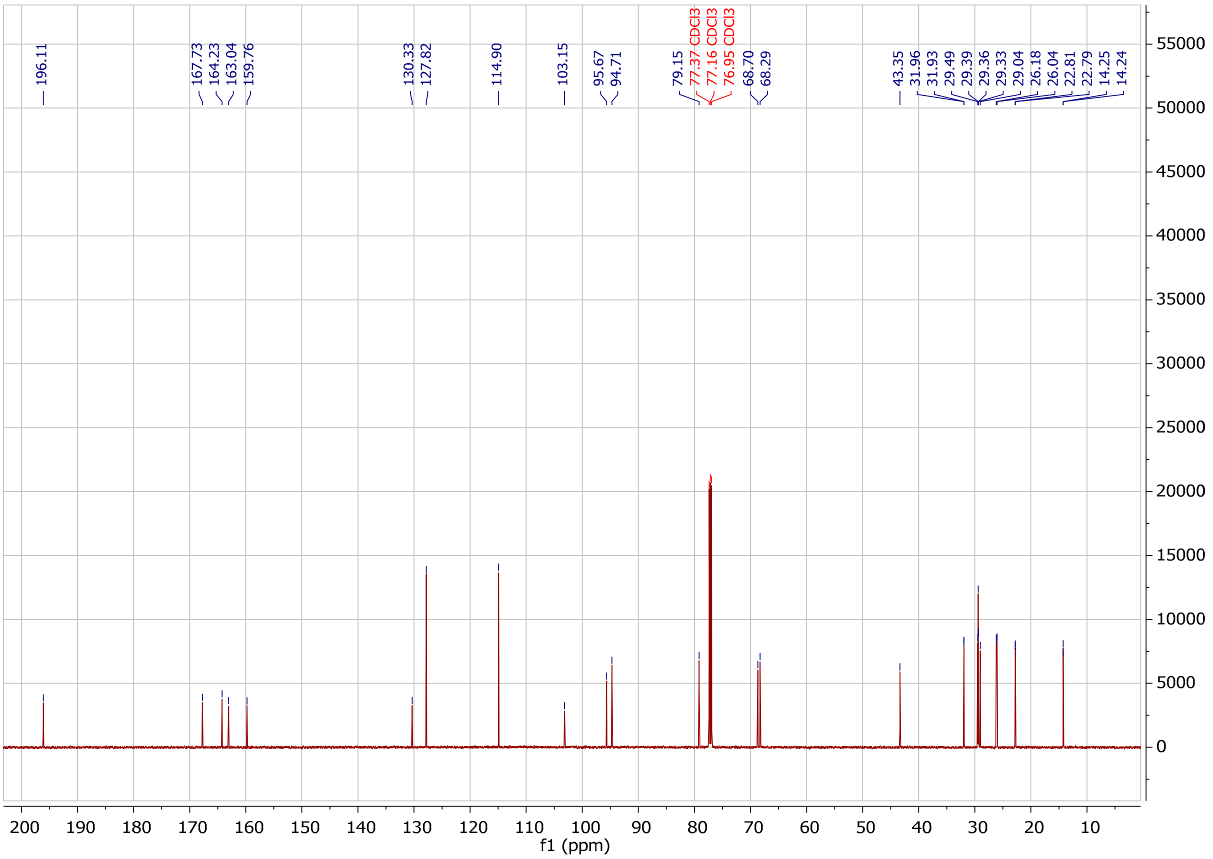

Figure S14. ^{13}C NMR (150 MHz, chloroform-*d*) spectrum of 7,4′-di-*O*-octylnaringenin (**A6**)

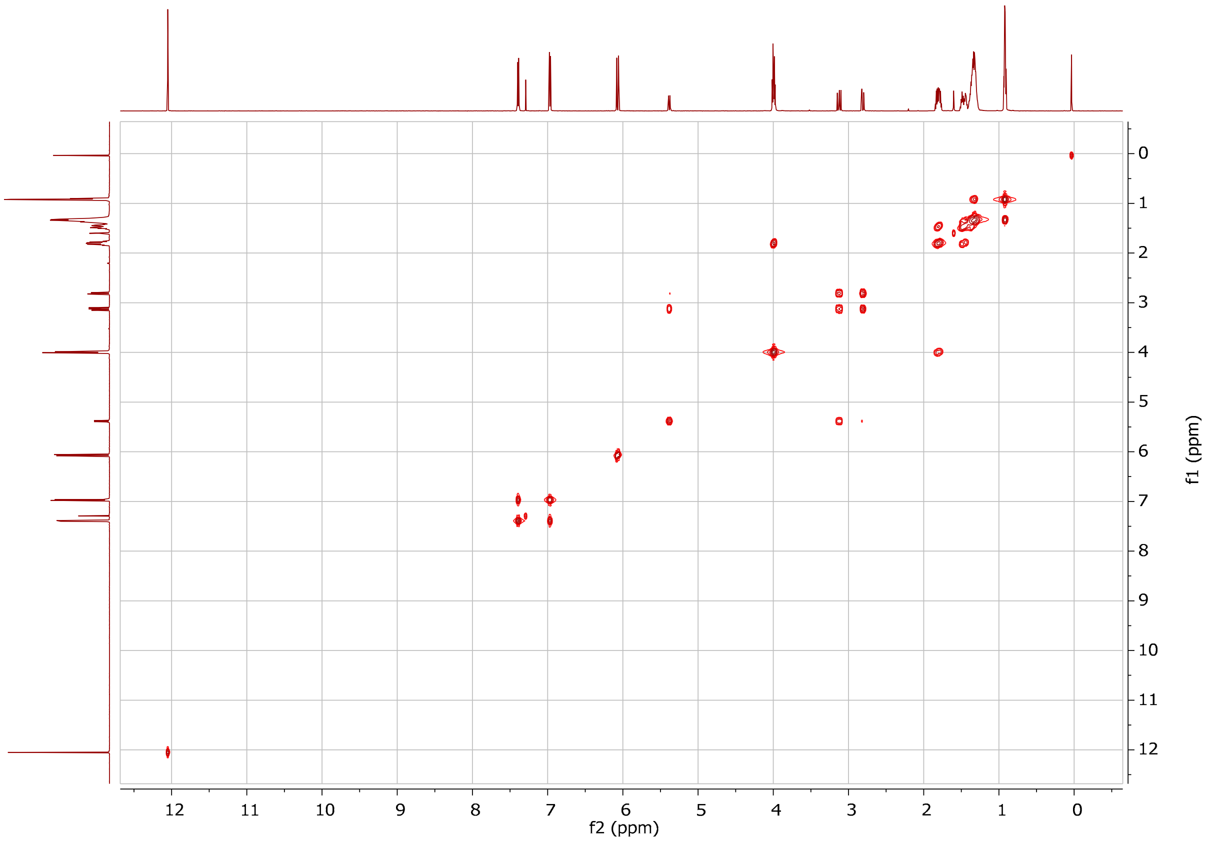

Figure S15. COSY NMR (150 MHz, chloroform-*d*) spectrum of 7,4′-di-*O*-octylnaringenin (**A6**)

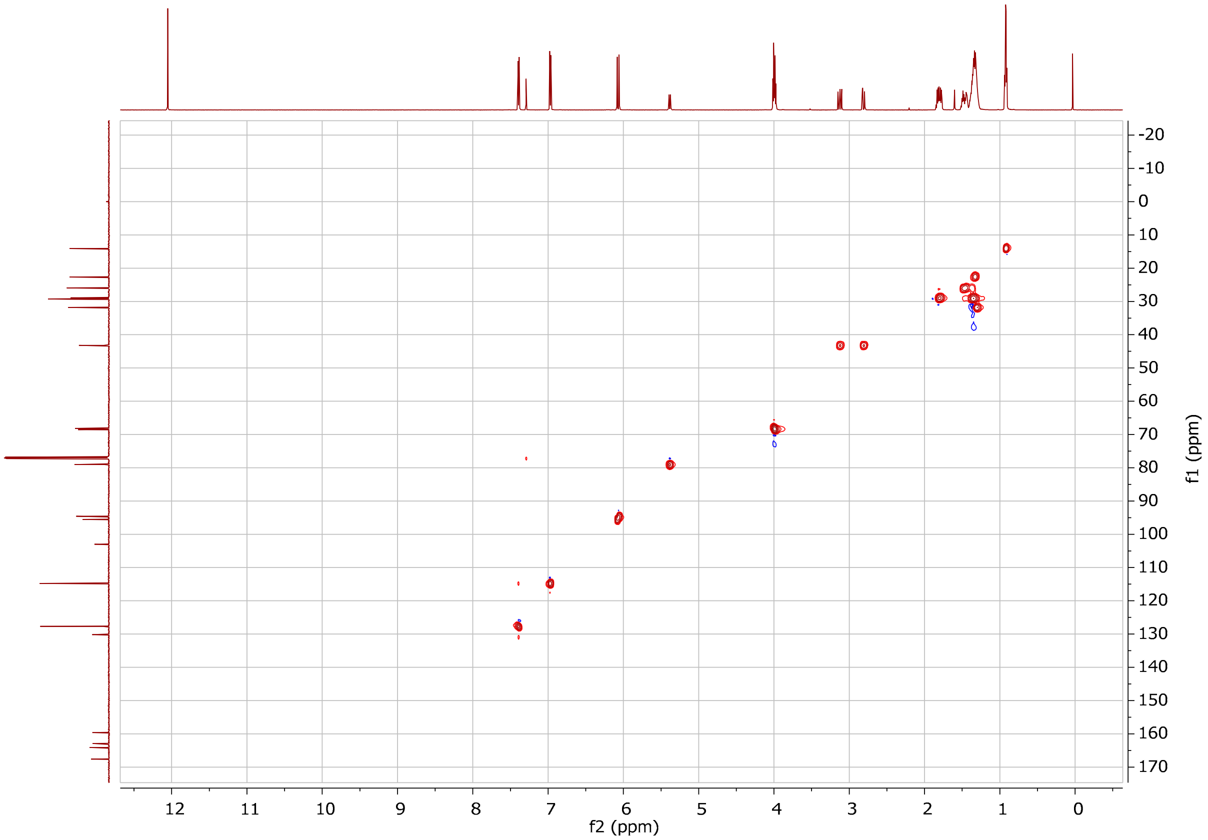

Figure S16. HSQC NMR (150 MHz, chloroform-*d*) spectrum of 7,4′-di-*O*-octylnaringenin (**A6**)

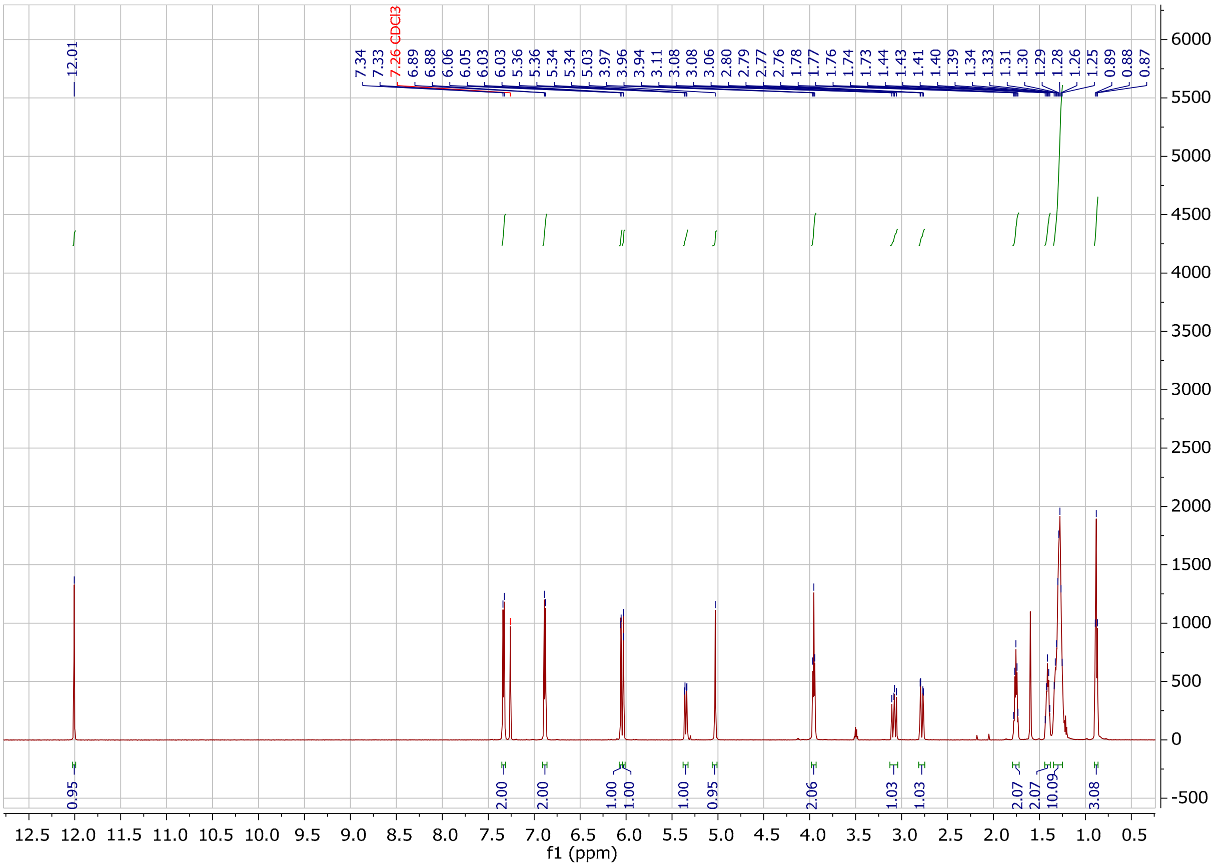

Figure S17. ^{1}H NMR (600 MHz, chloroform-*d*) spectrum of 7-*O*-nonylnaringenin (**A7**)

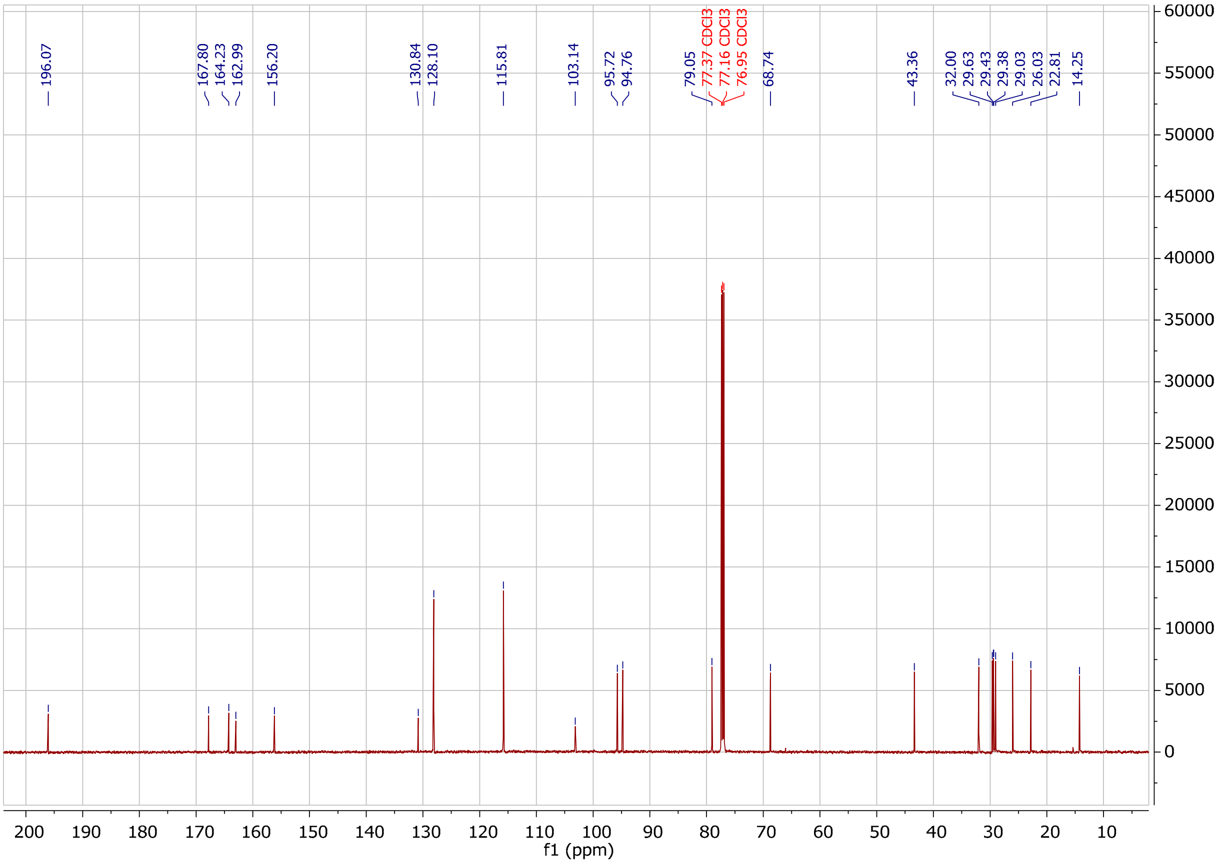

Figure S18. ^{13}C NMR (150 MHz, chloroform-*d*) spectrum of 7-*O*-nonylnaringenin (**A7**)

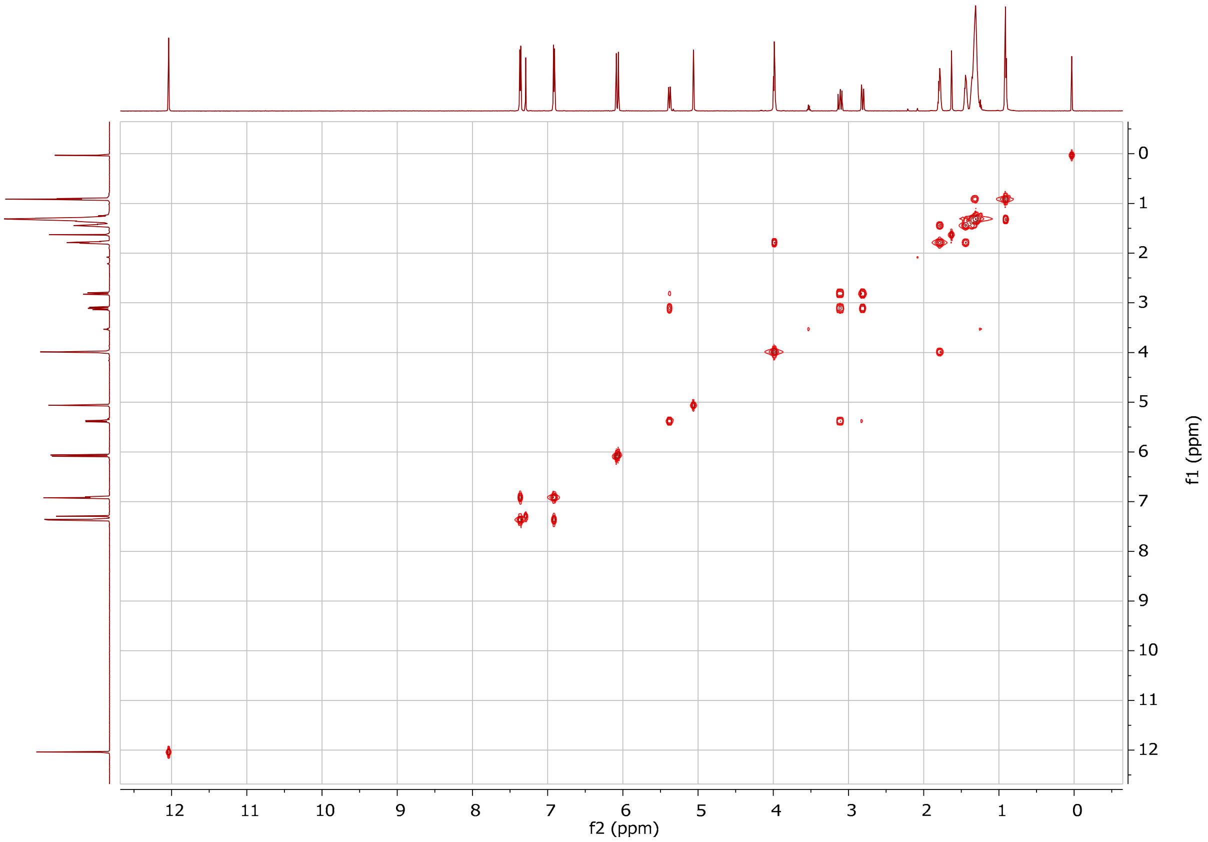

Figure S19. COSY NMR (150 MHz, chloroform-*d*) spectrum of 7-*O*-nonylnaringenin (**A7**)

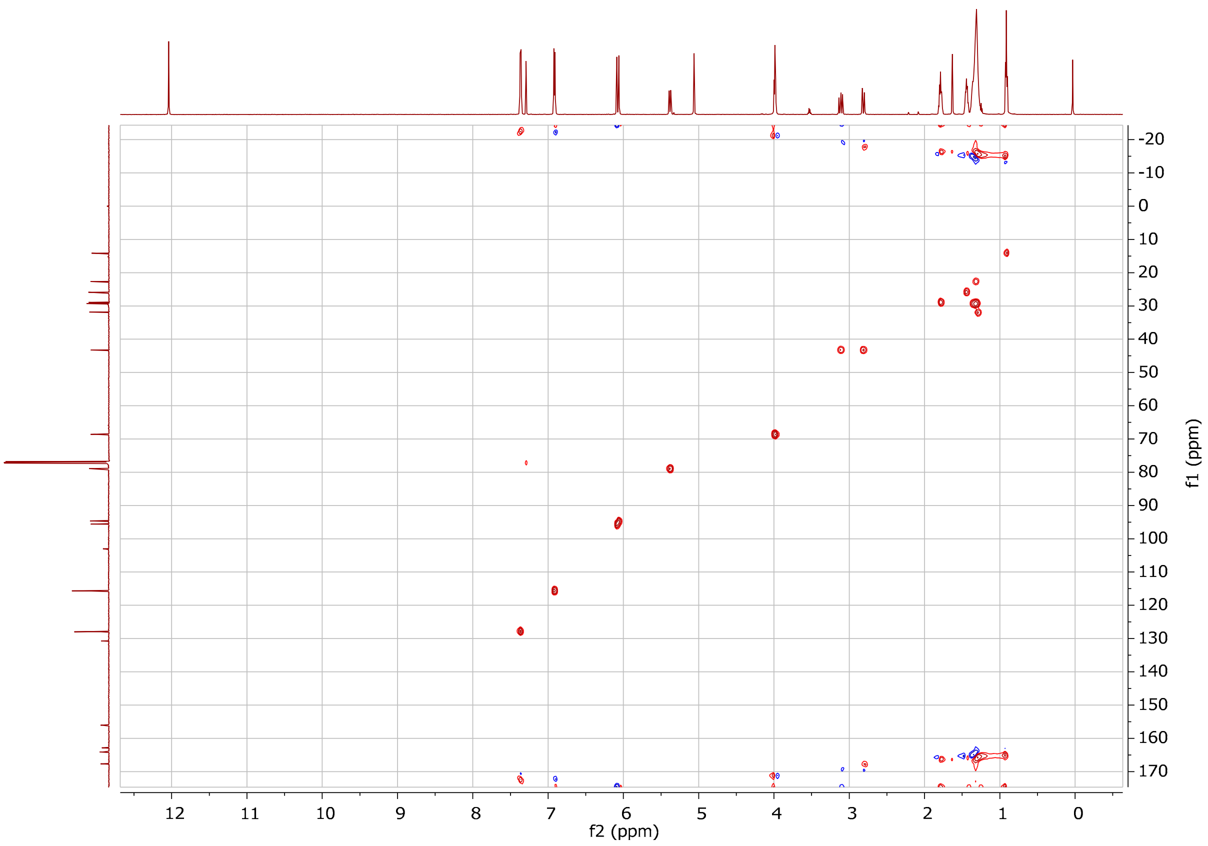

Figure S20. HSQC NMR (150 MHz, chloroform-*d*) spectrum of 7-*O*-nonylnaringenin (**A7**)

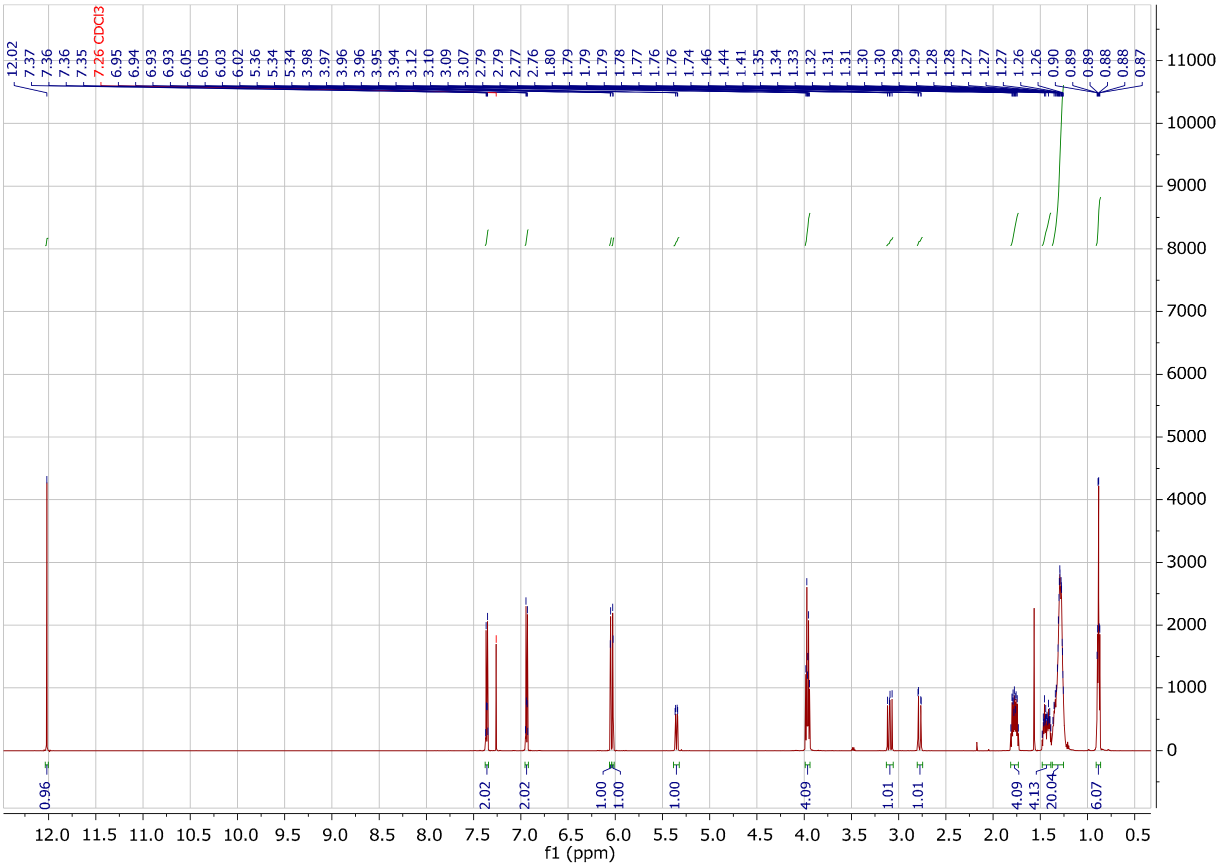

Figure S21. ^{1}H NMR (600 MHz, chloroform-*d*) spectrum of 7,4′-di-*O*-nonylnaringenin (**A8**)

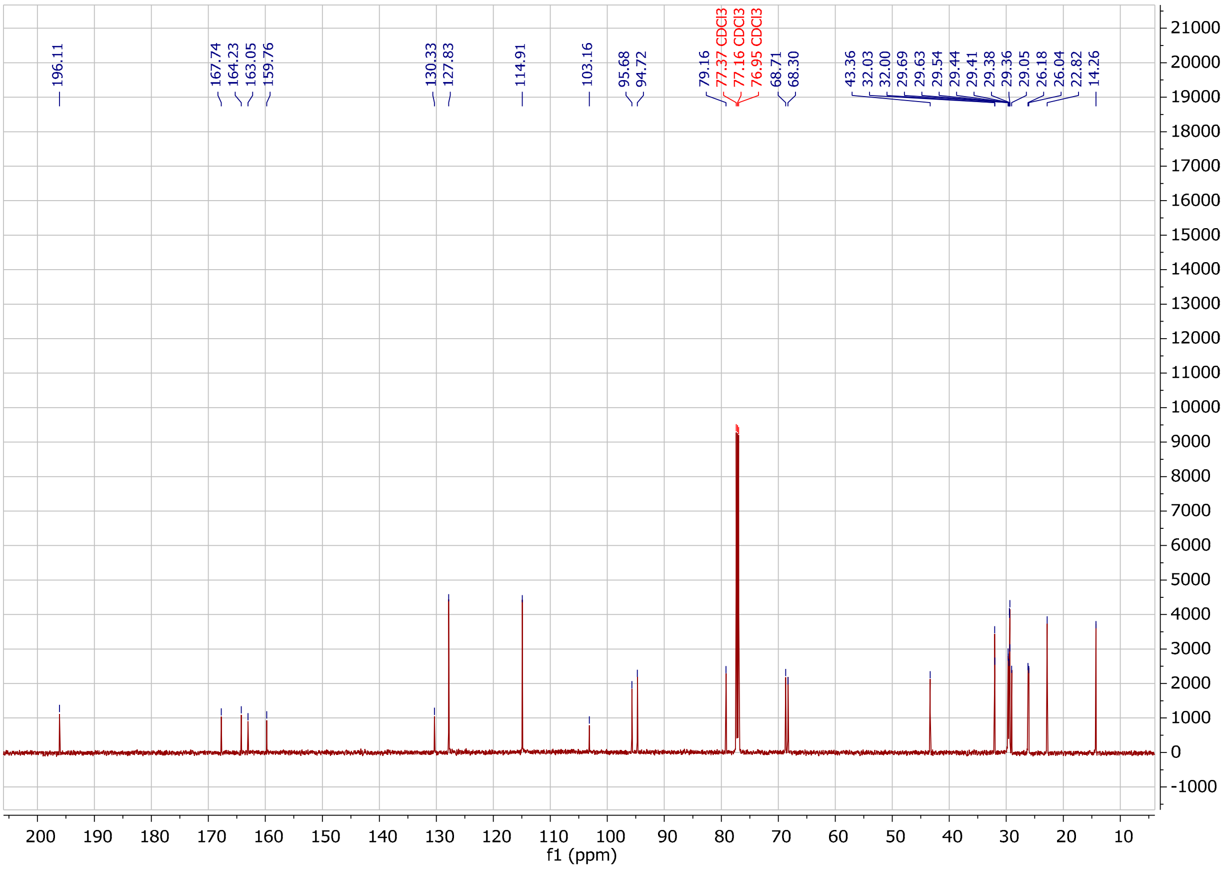

Figure S22. ^{13}C NMR (150 MHz, chloroform-*d*) spectrum of 7,4′-di-*O*-nonylnaringenin (**A8**)

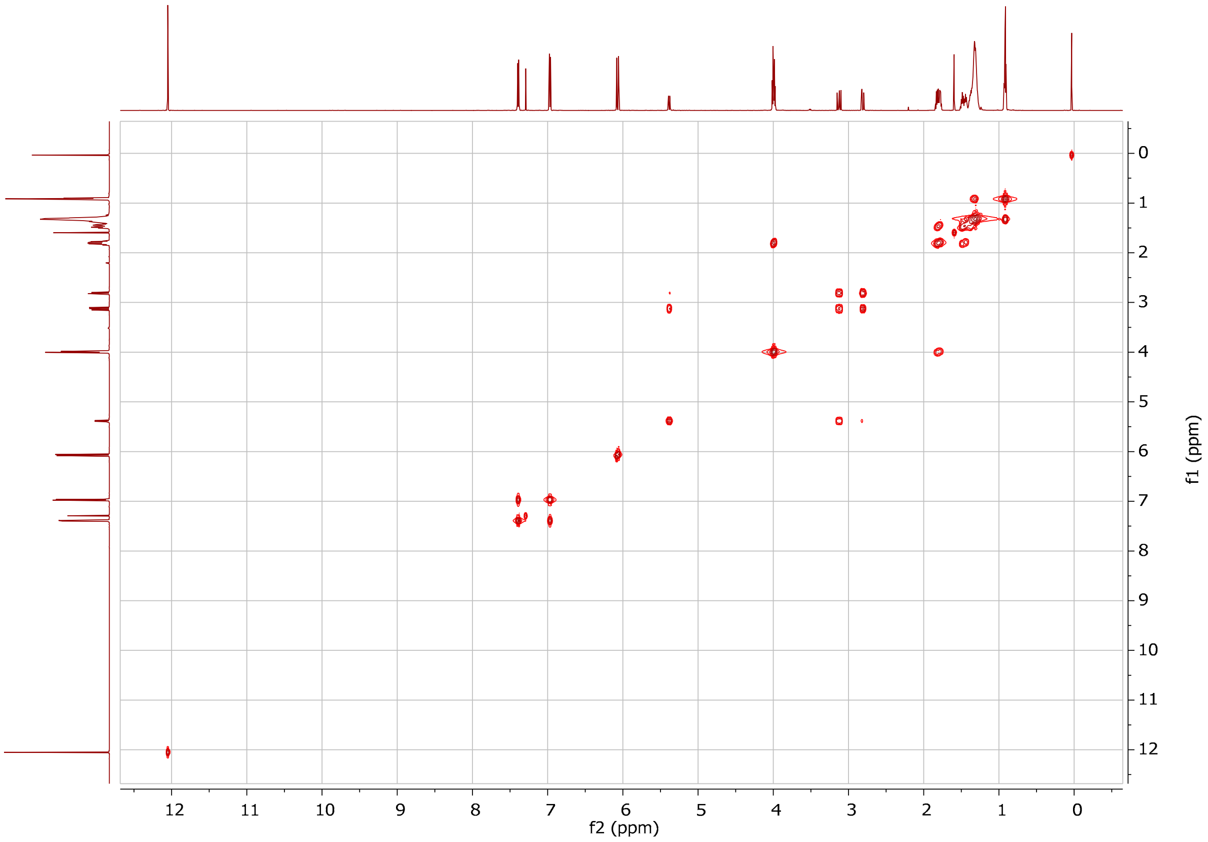

Figure S23. COSY NMR (150 MHz, chloroform-*d*) spectrum of 7,4′-di-*O*-nonylnaringenin (**A8**)

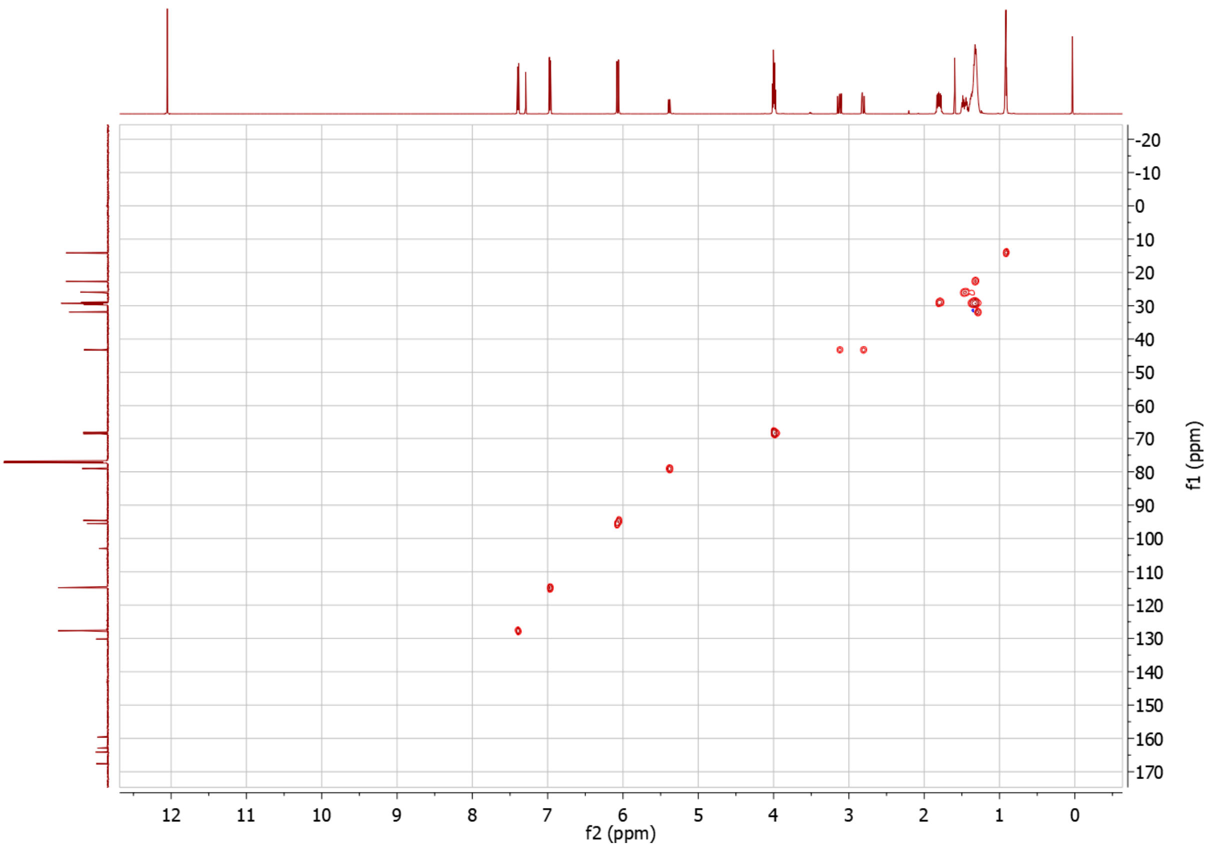

Figure S24. HSQC NMR (150 MHz, chloroform-*d*) spectrum of 7,4′-di-*O*-nonylnaringenin (**A8**)

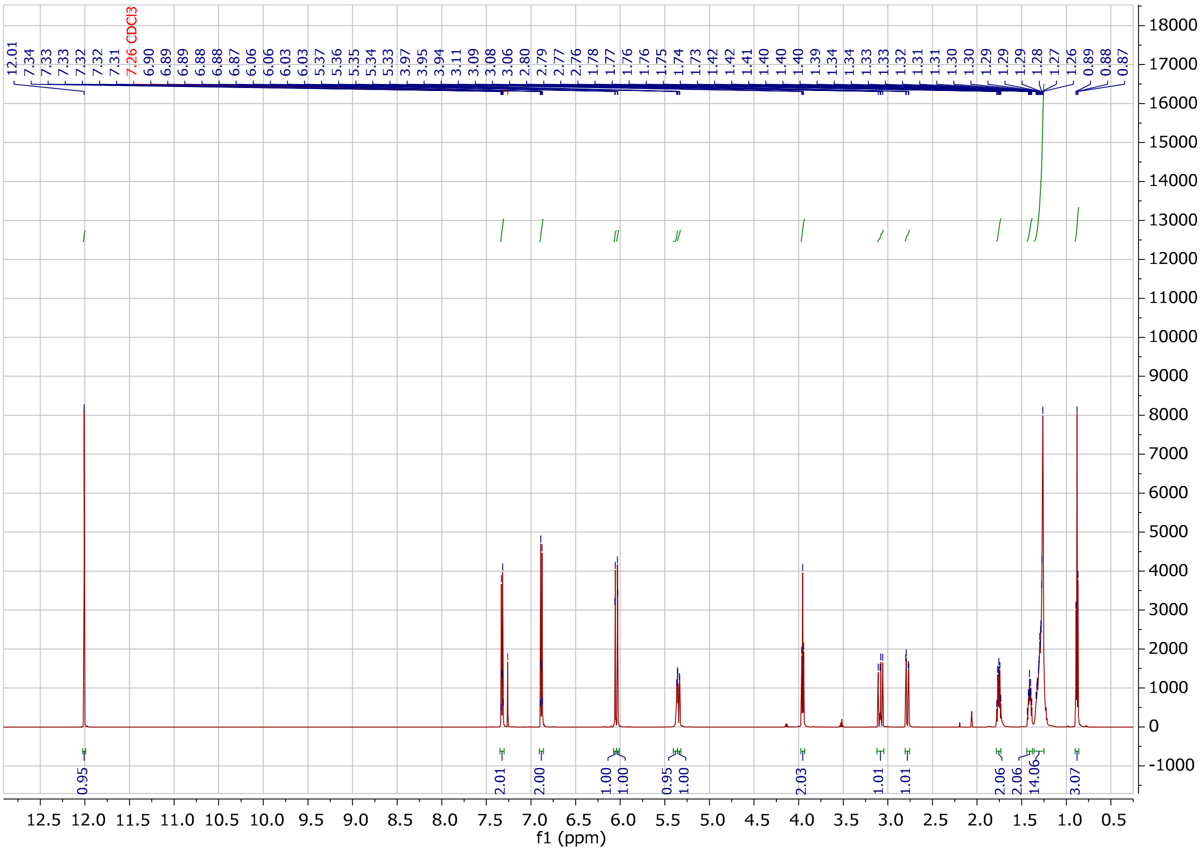

Figure S25. ^{1}H NMR (600 MHz, chloroform-*d*) spectrum of 7-*O*-undecylnaringenin (**A9**)

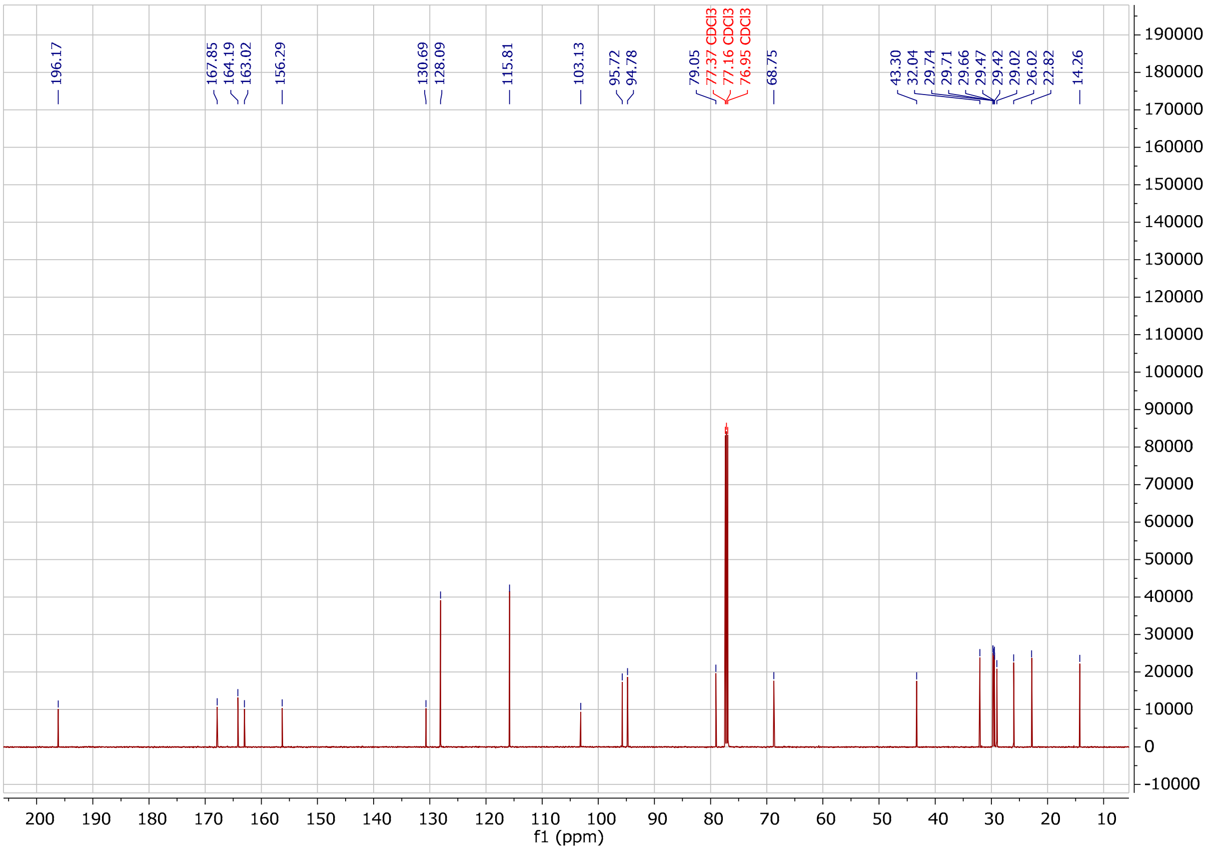

Figure S26. ^{13}C NMR (150 MHz, chloroform-*d*) spectrum of 7-*O*-undecylnaringenin (**A9**)

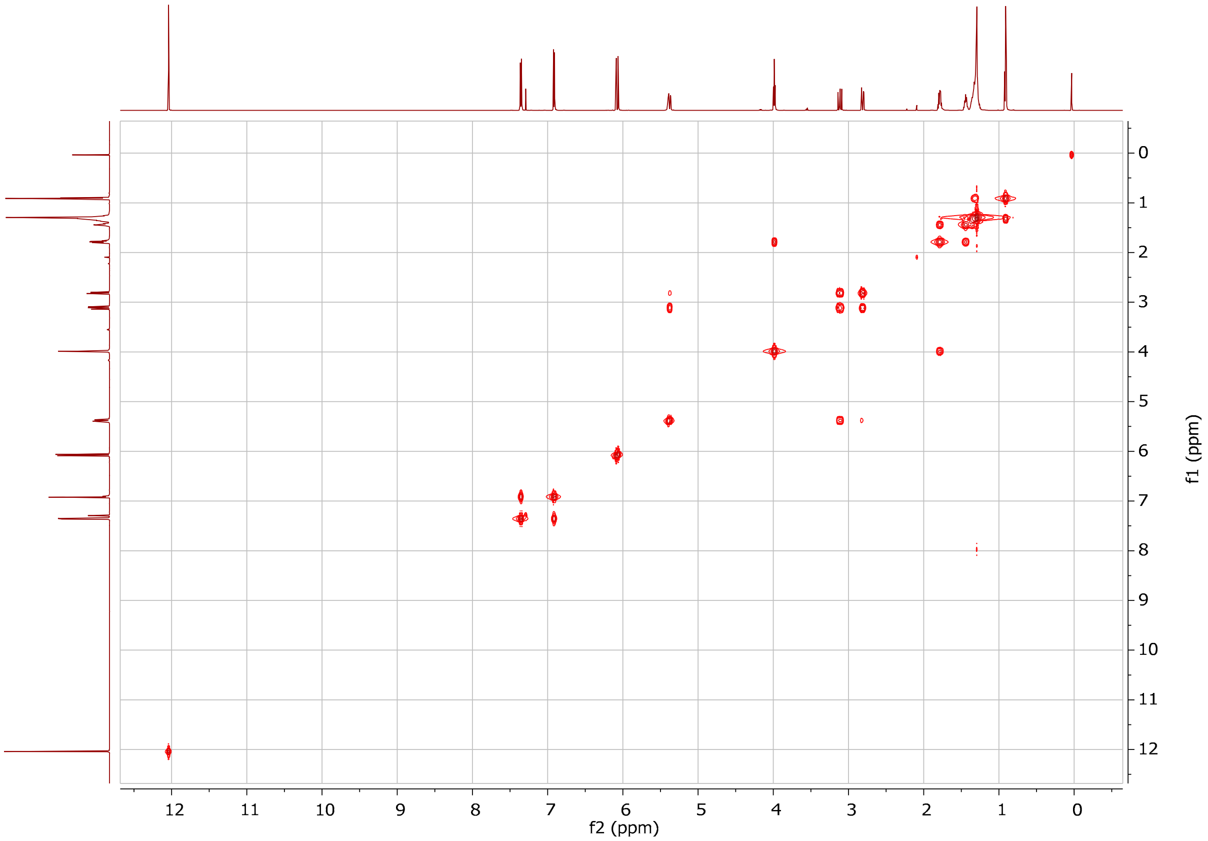

Figure S27. COSY NMR (150 MHz, chloroform-*d*) spectrum of 7-*O*-undecylnaringenin (**A9**)

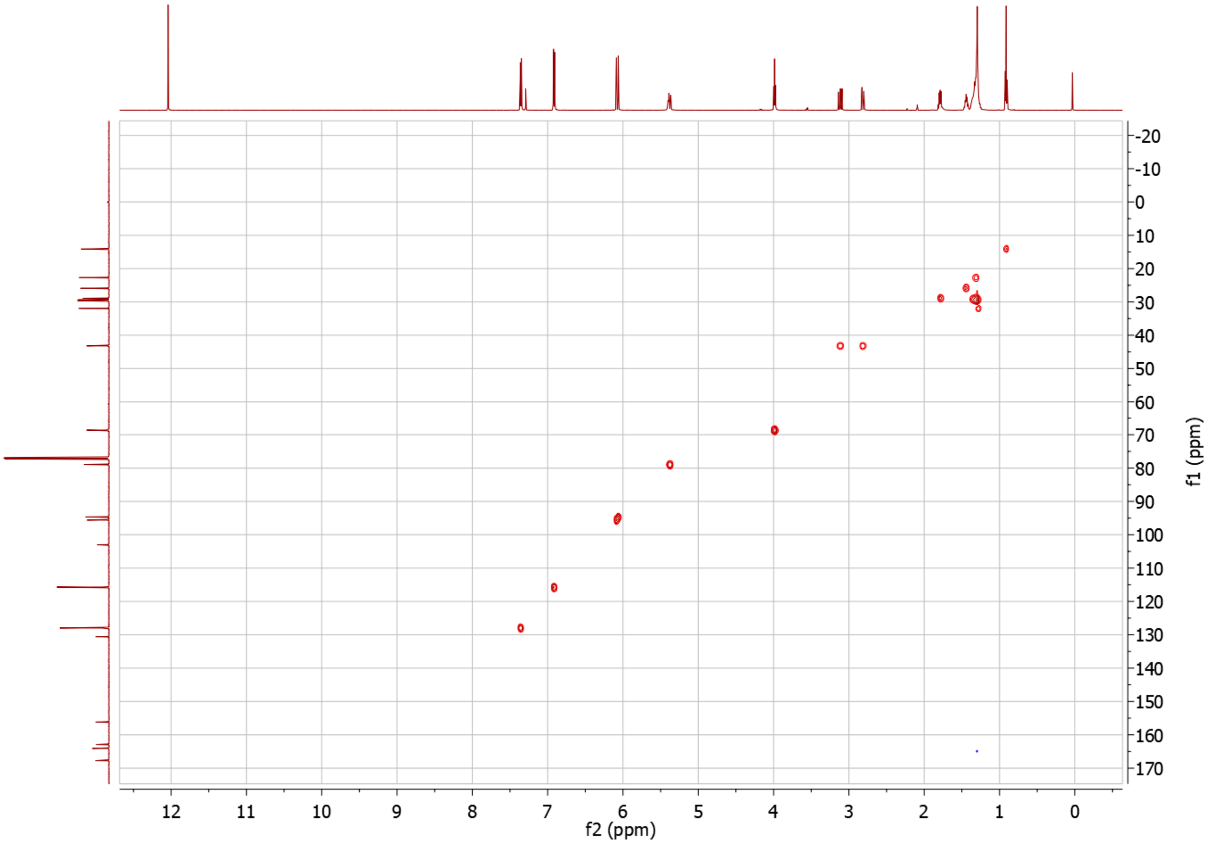

Figure S28. HSQC NMR (150 MHz, chloroform-*d*) spectrum of 7-*O*-undecylnaringenin (**A9**)

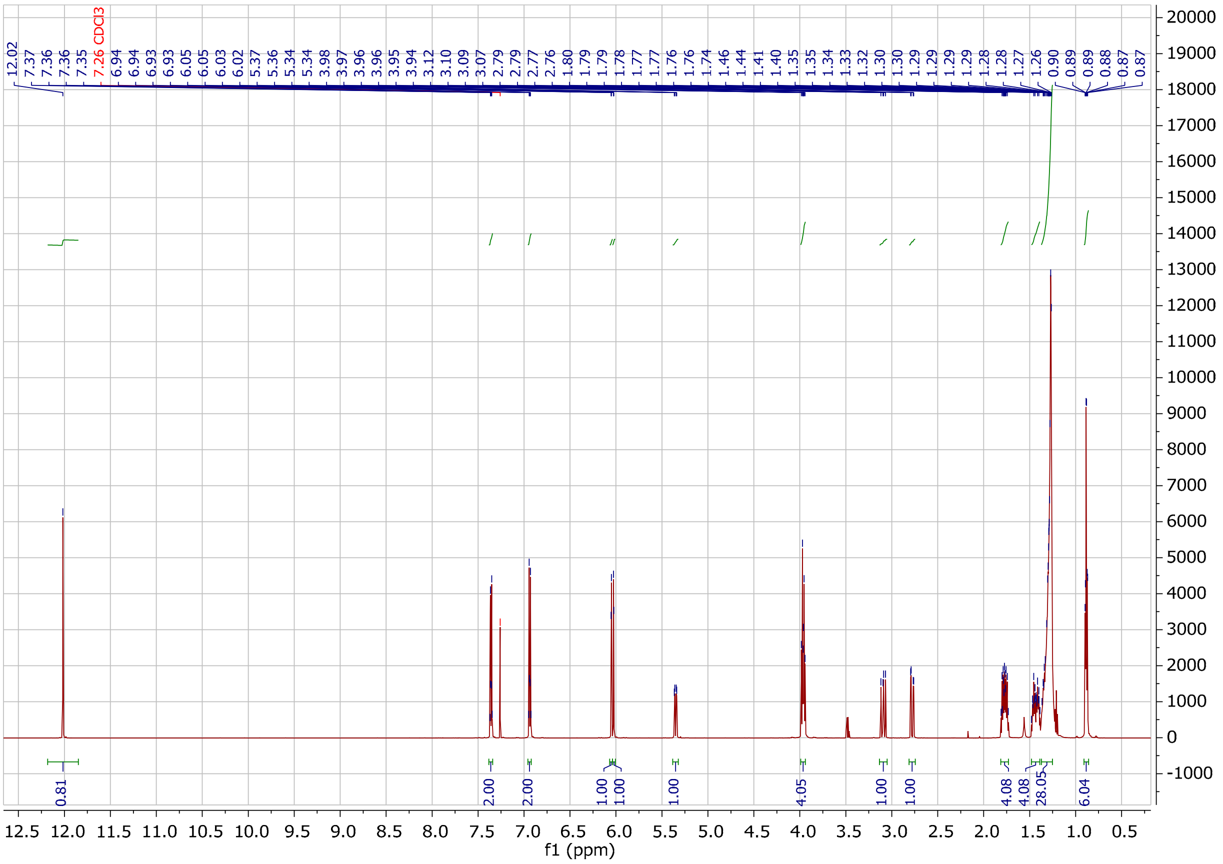

Figure S29. ^{1}H NMR (600 MHz, chloroform-*d*) spectrum of 7,4′-di-*O*-undecylnaringenin (**A10**)

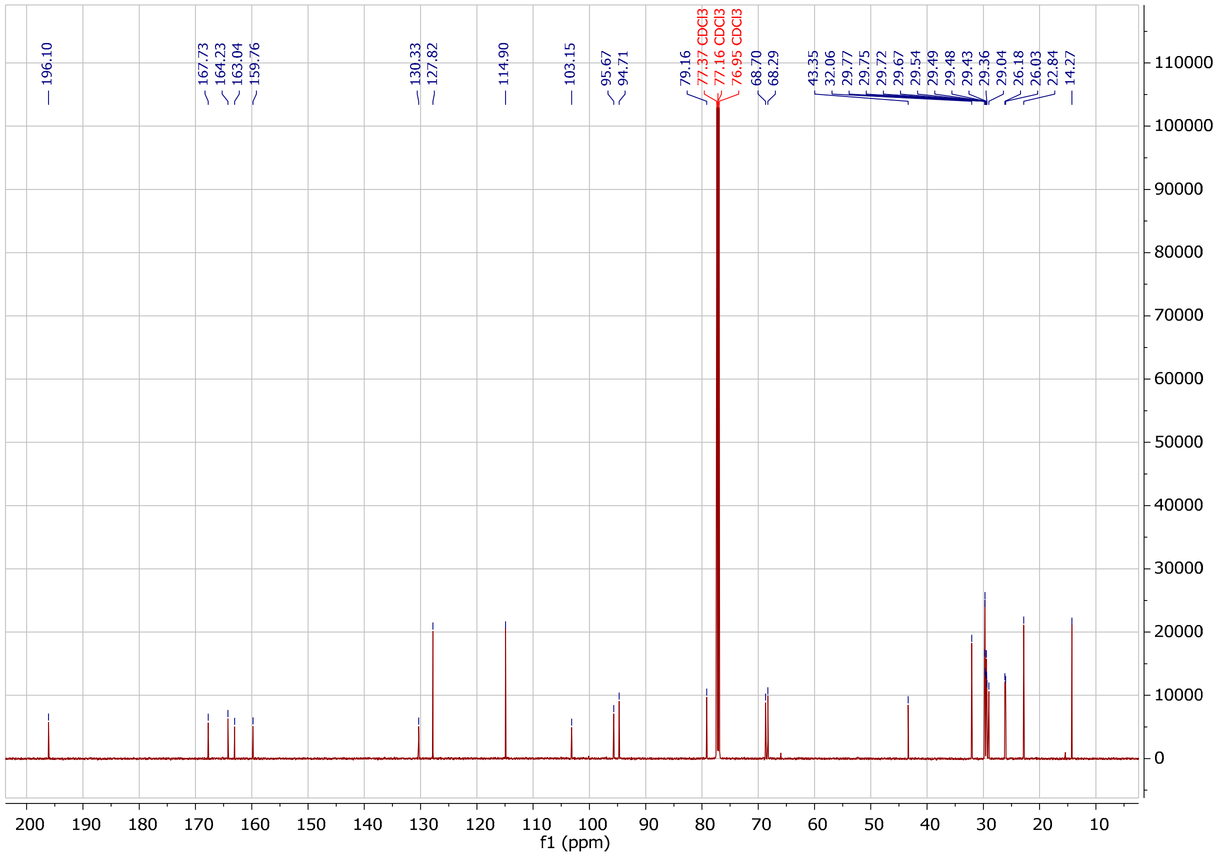

Figure S30. ^{13}C NMR (150 MHz, chloroform-*d*) spectrum of 7,4′-di-*O*-undecylnaringenin (**A10**)

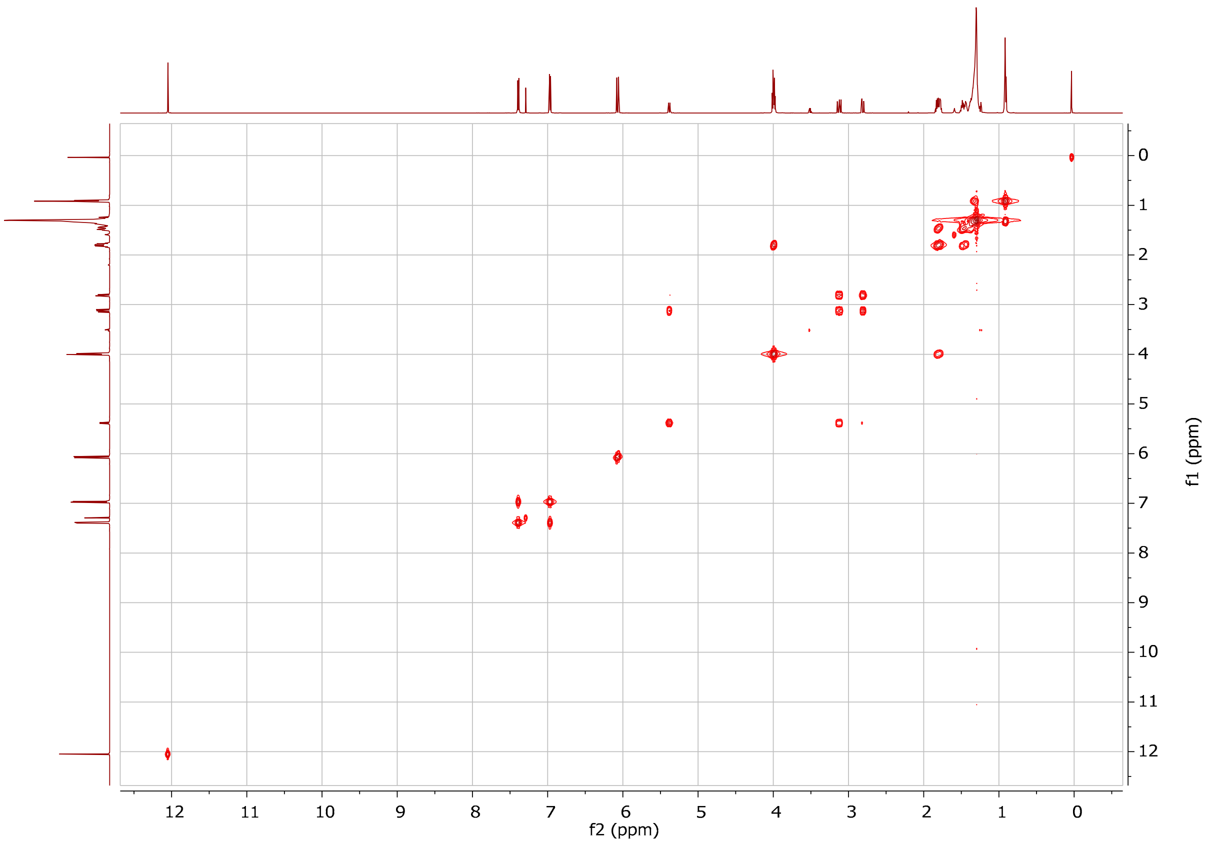

Figure S31. COSY NMR (150 MHz, chloroform-*d*) spectrum of 7,4′-di-*O*-undecylnaringenin (**A10**)

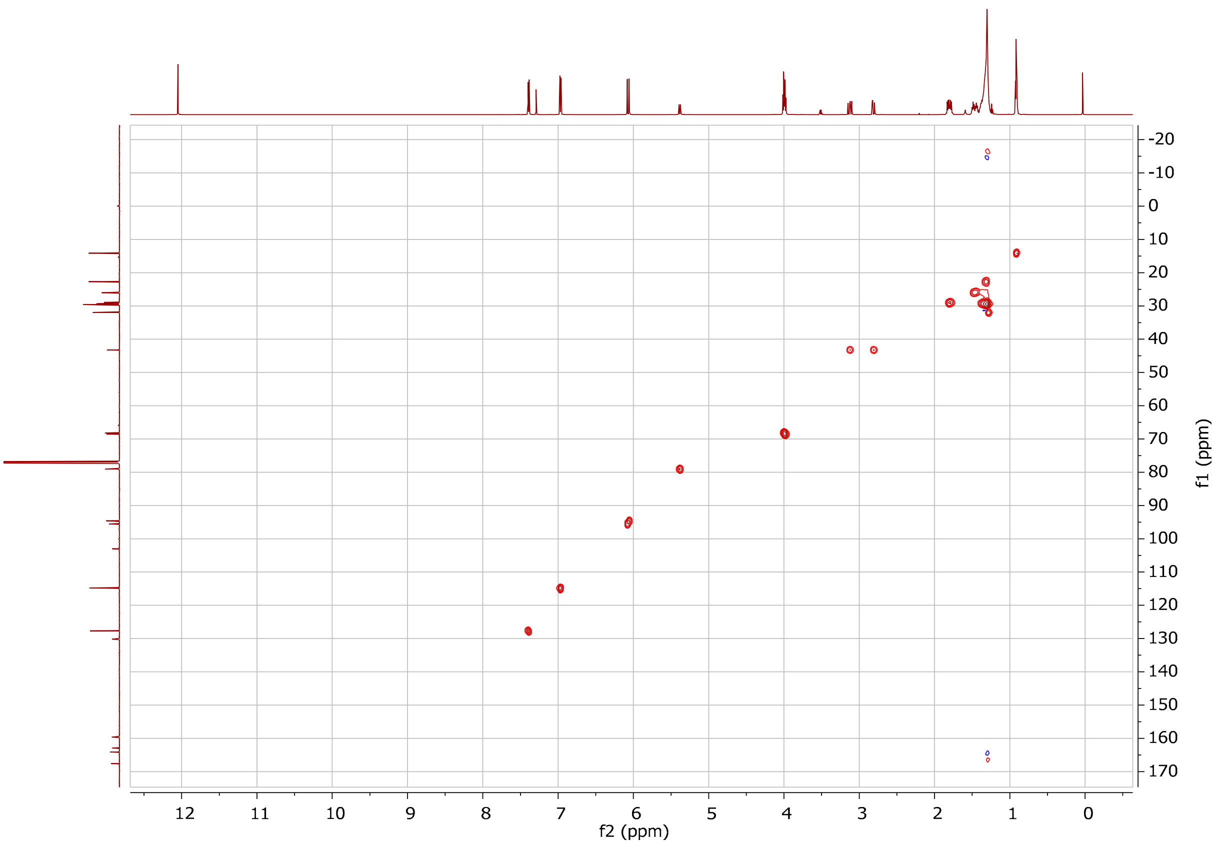

Figure S32. HSQC NMR (150 MHz, chloroform-*d*) spectrum of 7,4′-di-*O*-undecylnaringenin (**A10**)

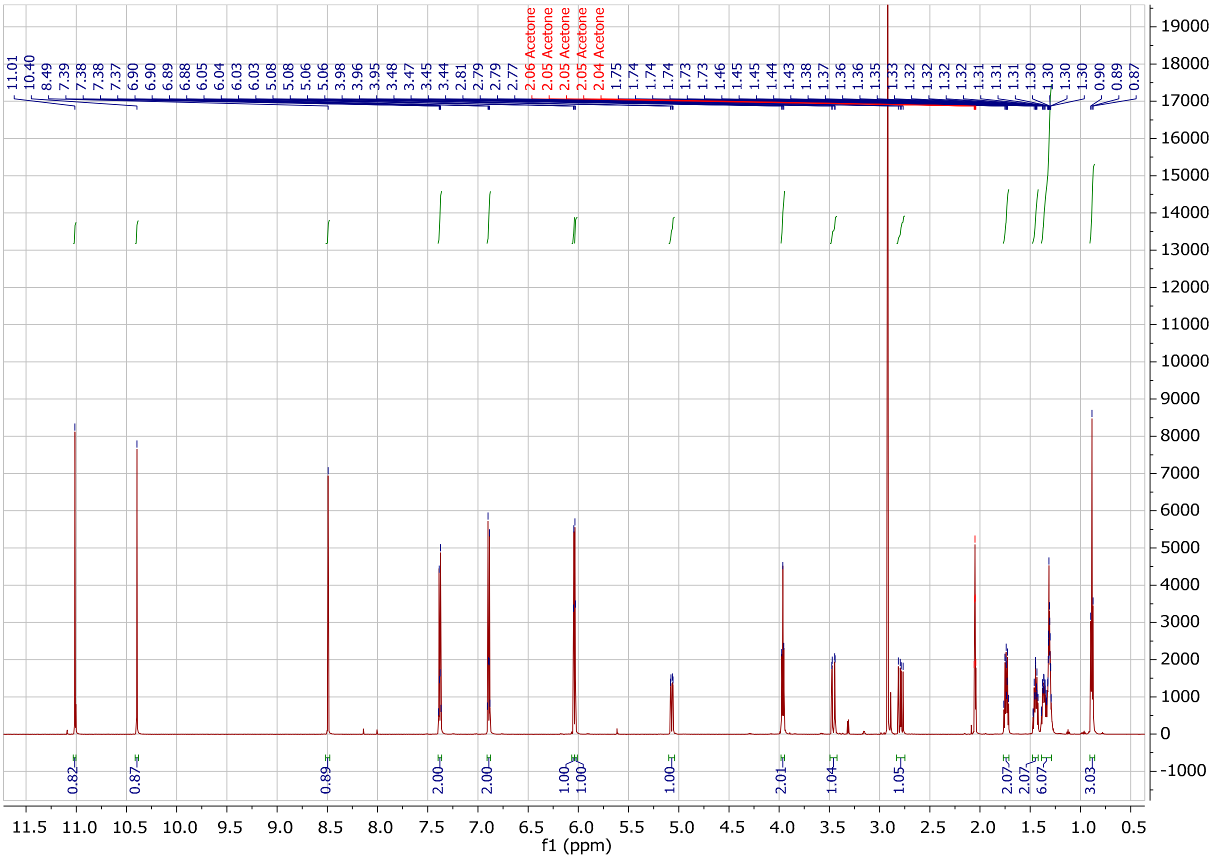

Figure S33. ^{1}H NMR (600 MHz, acetone-d_6) spectrum of 7-*O*-heptylnaringenin oxime (**B3**)

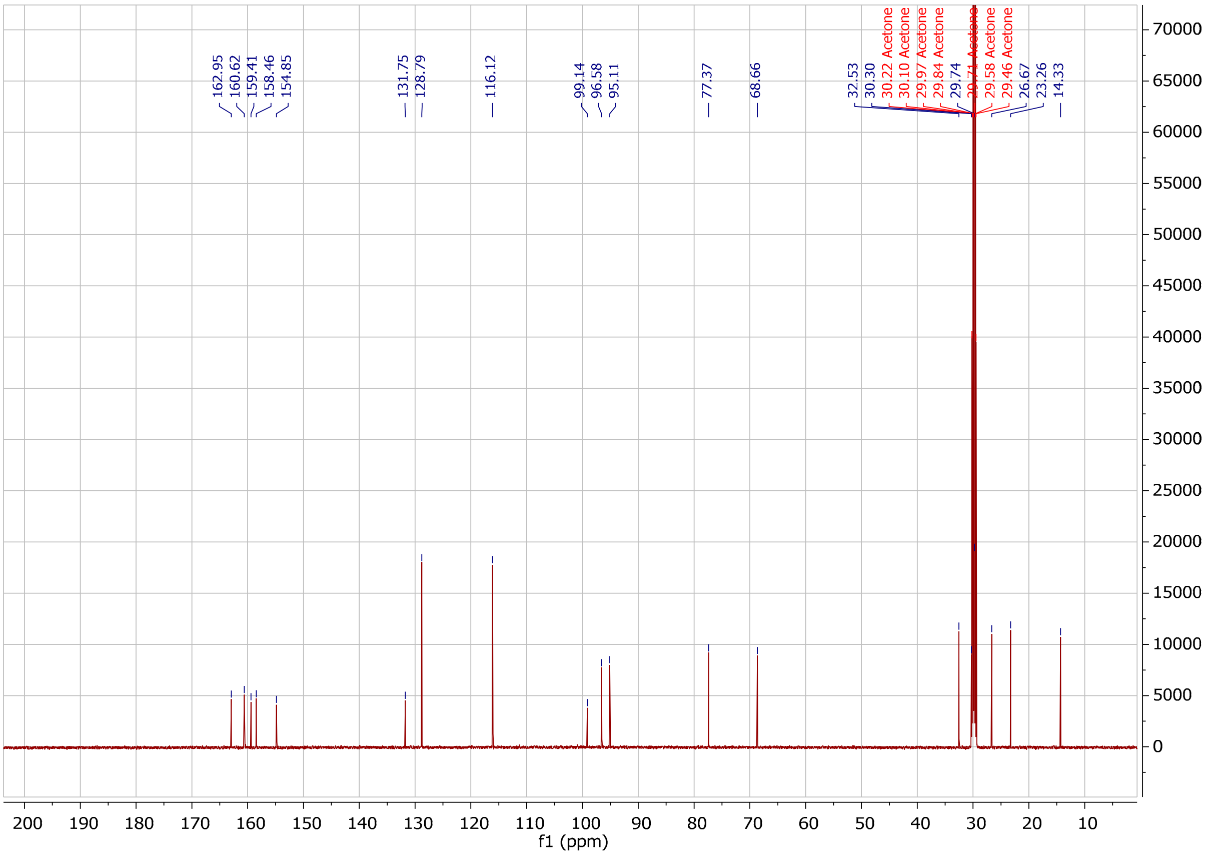

Figure S34. ^{13}C NMR (150 MHz, acetone-d_6) spectrum of 7-*O*-heptylnaringenin oxime (**B3**)

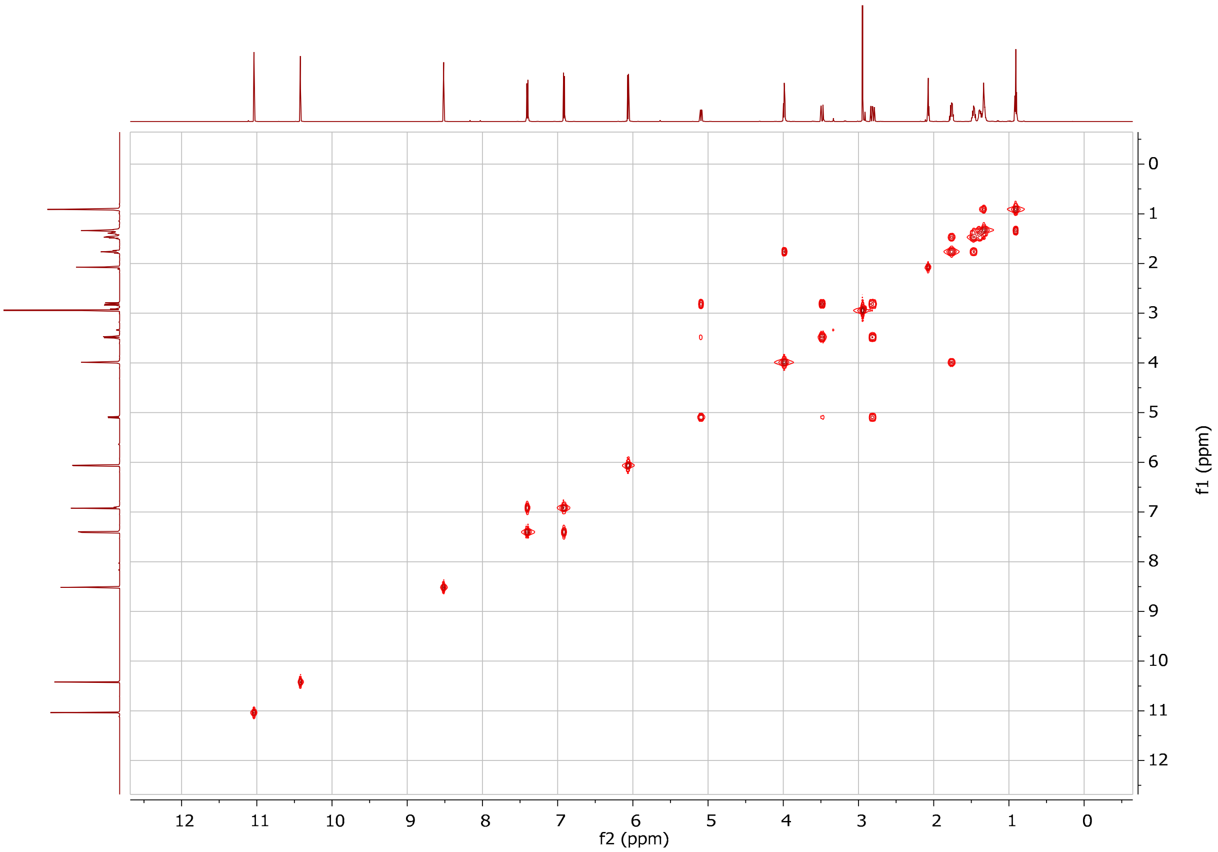

Figure S35. COSY NMR (150 MHz, acetone-d_6) spectrum of 7-*O*-heptylnaringenin oxime (**B3**)

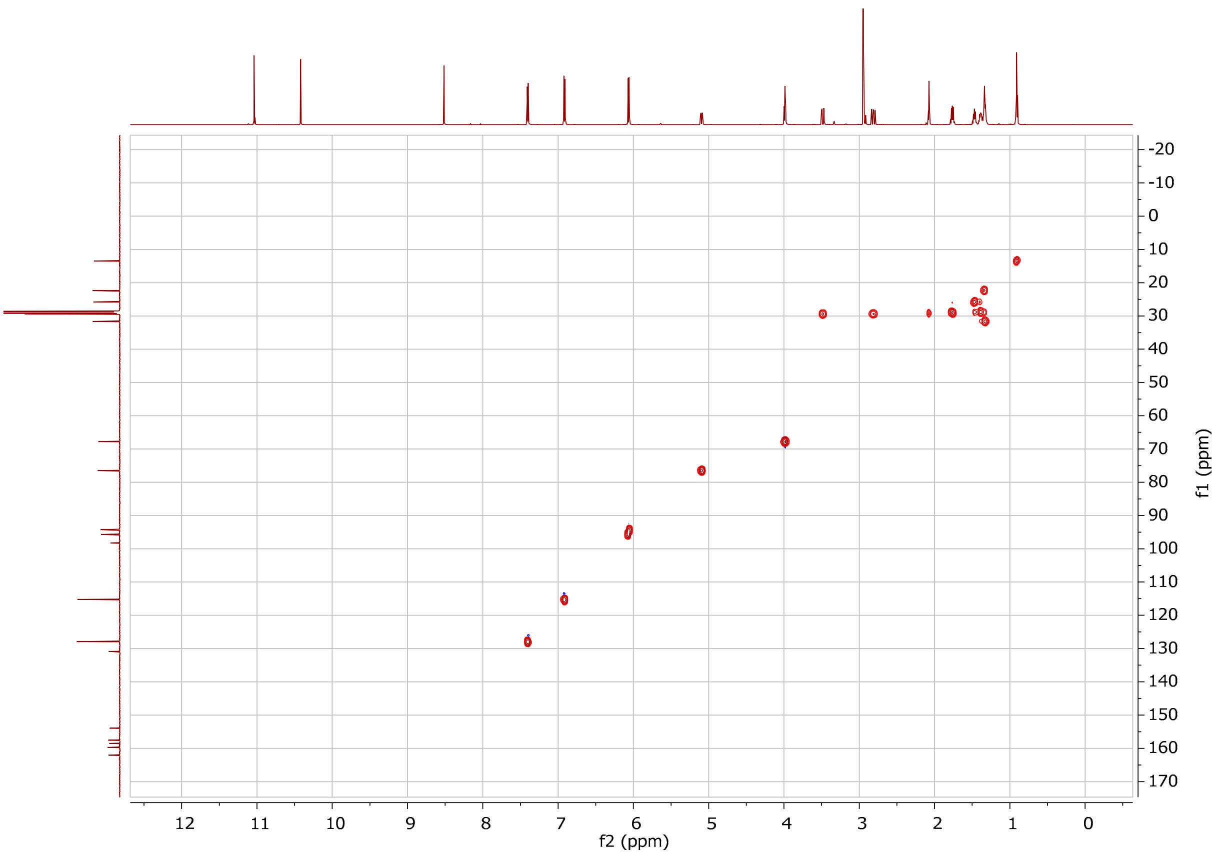

Figure S36. HSQC NMR (150 MHz, acetone-d_6) spectrum of 7-*O*-heptylnaringenin oxime (**B3**)

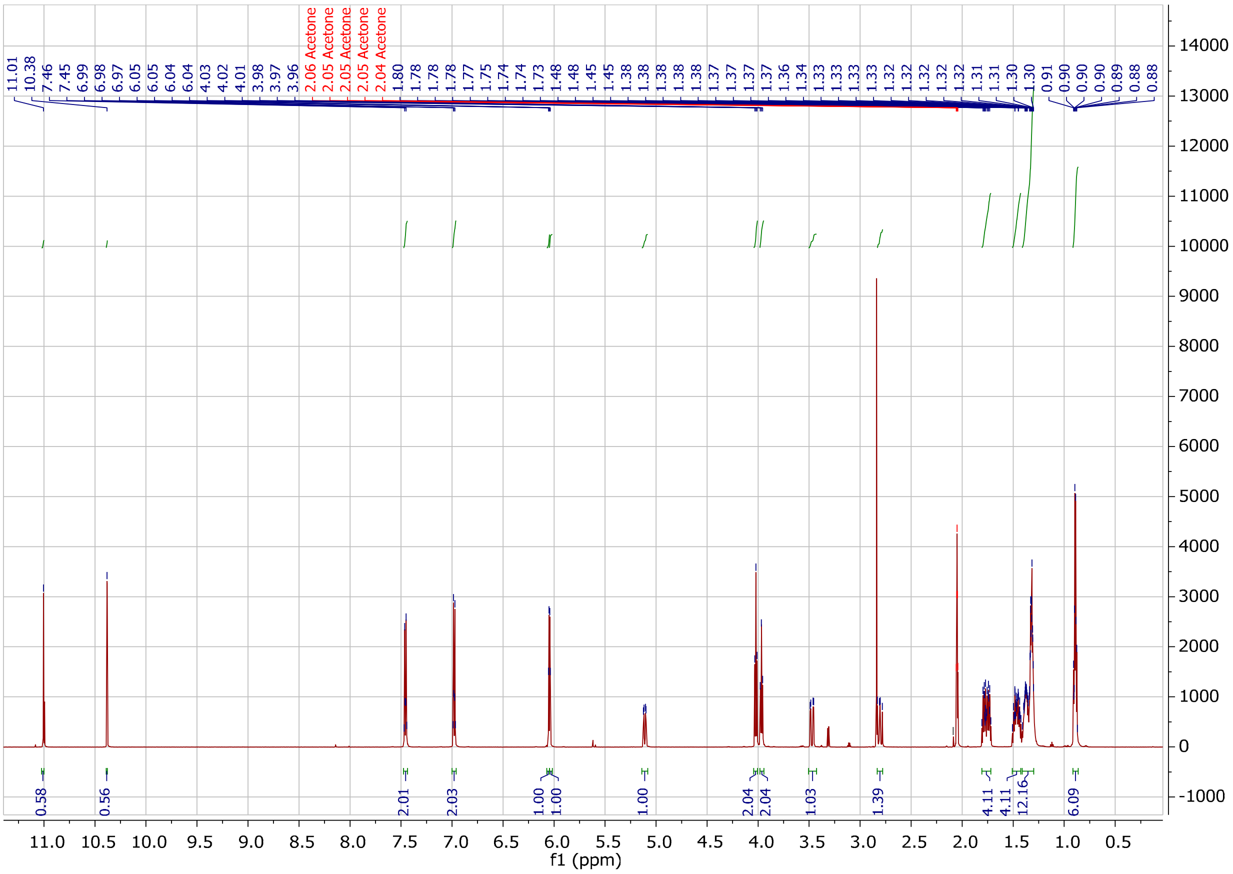

Figure S37. ^{1}H NMR (600 MHz, acetone-d_6) spectrum of 7,4′-di-*O*-heptylnaringenin oxime (**B4**)

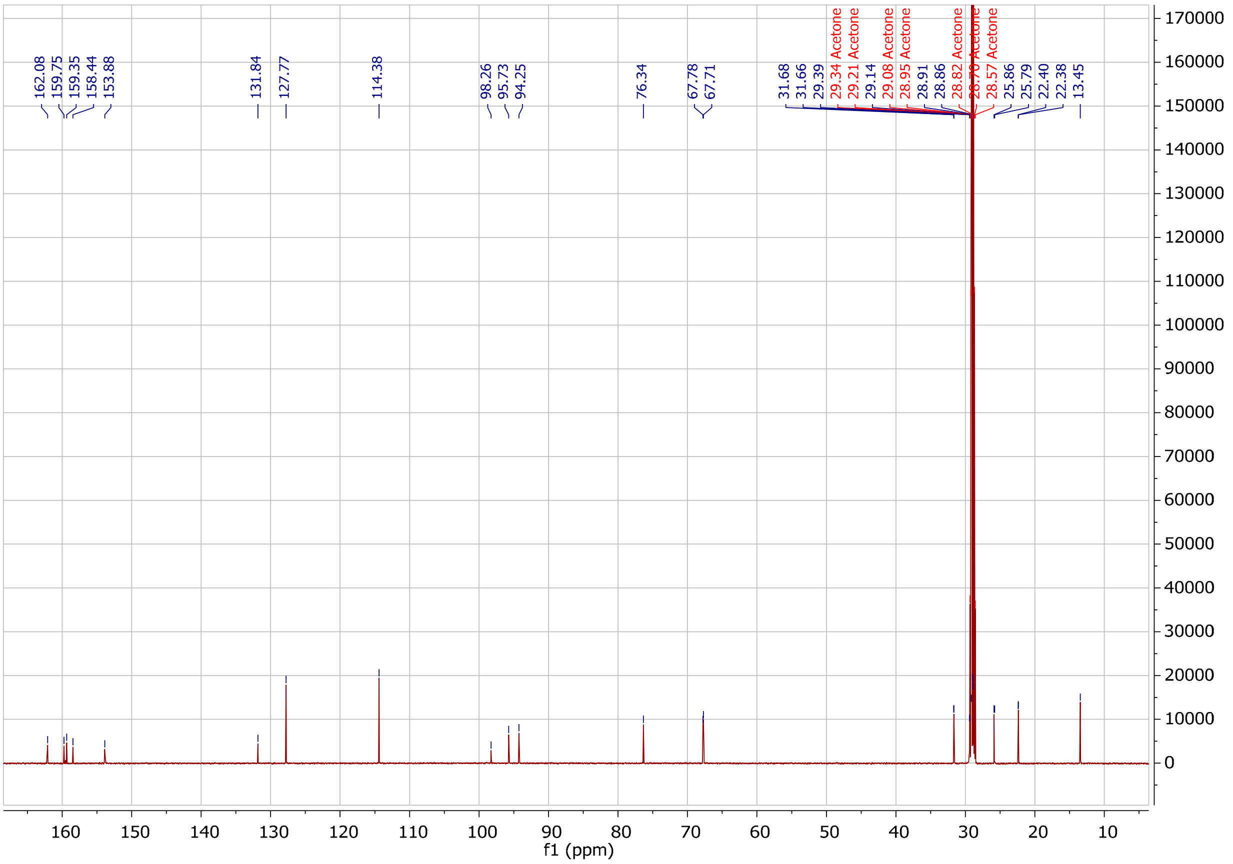

Figure S38. ^{13}C NMR (150 MHz, acetone-d_6) spectrum of 7,4′-di-*O*-heptylnaringenin oxime (**B4**)

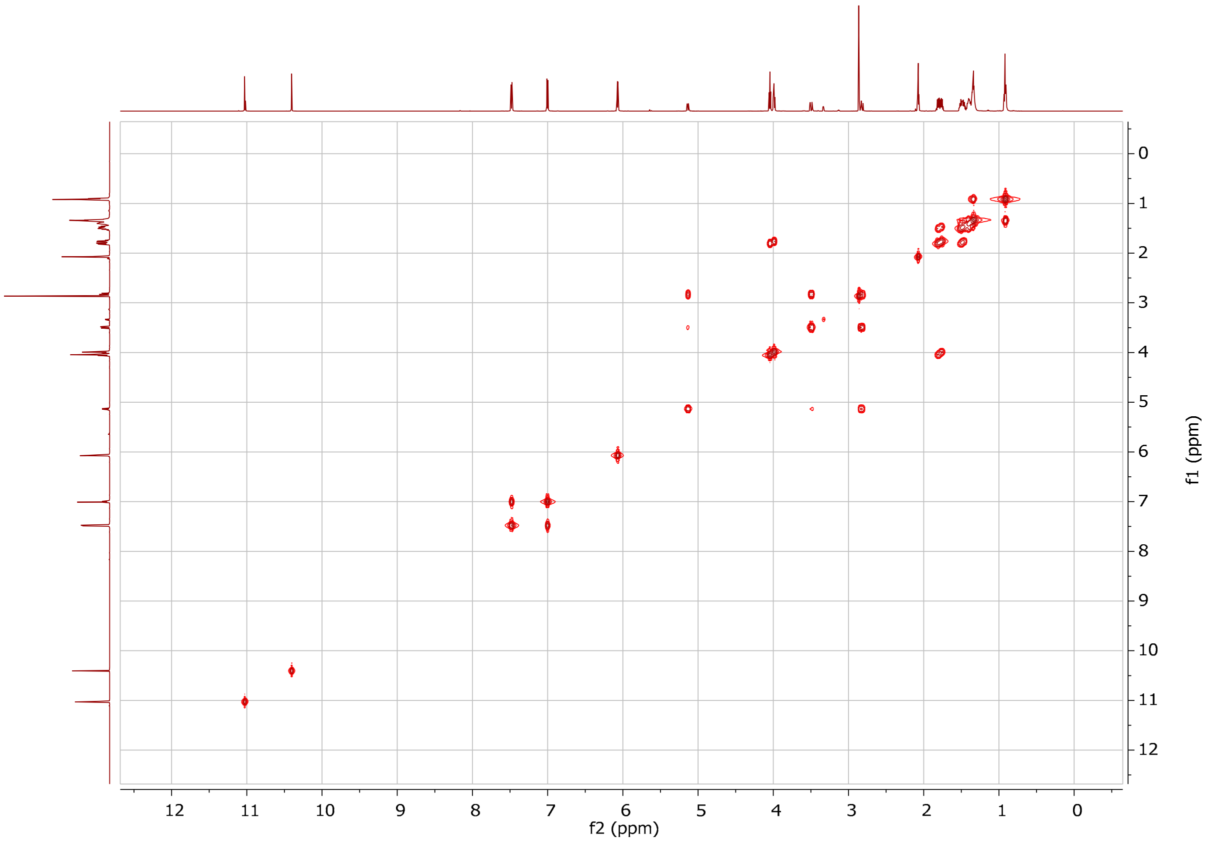

Figure S39. COSY NMR (150 MHz, acetone-d_6) spectrum of 7,4′-di-*O*-heptylnaringenin oxime (**B4**)

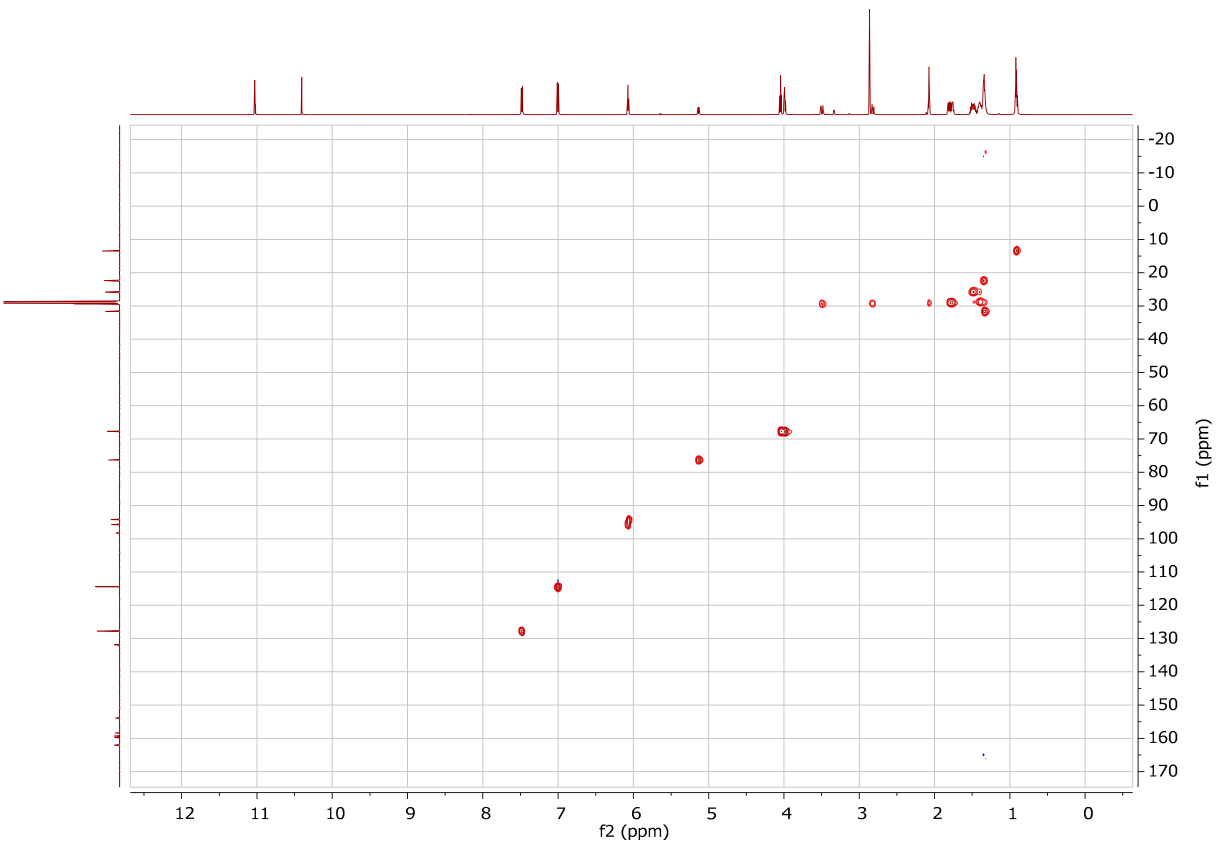

Figure S40. HSQC NMR (150 MHz, acetone-d_6) spectrum of 7,4′-di-*O*-heptylnaringenin oxime (**B4**)

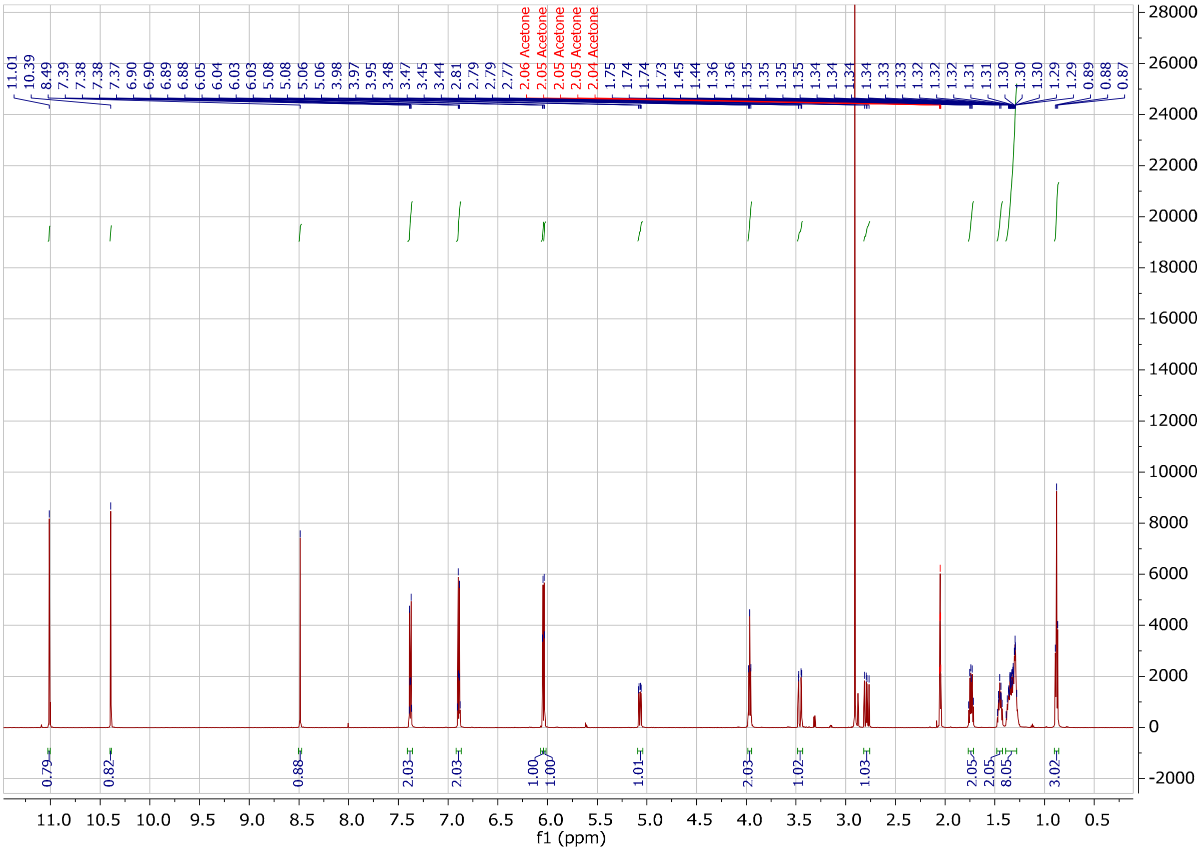

Figure S41. ^{1}H NMR (600 MHz, acetone-d_6) spectrum of 7-*O*-octylnaringenin oxime (**B5**)

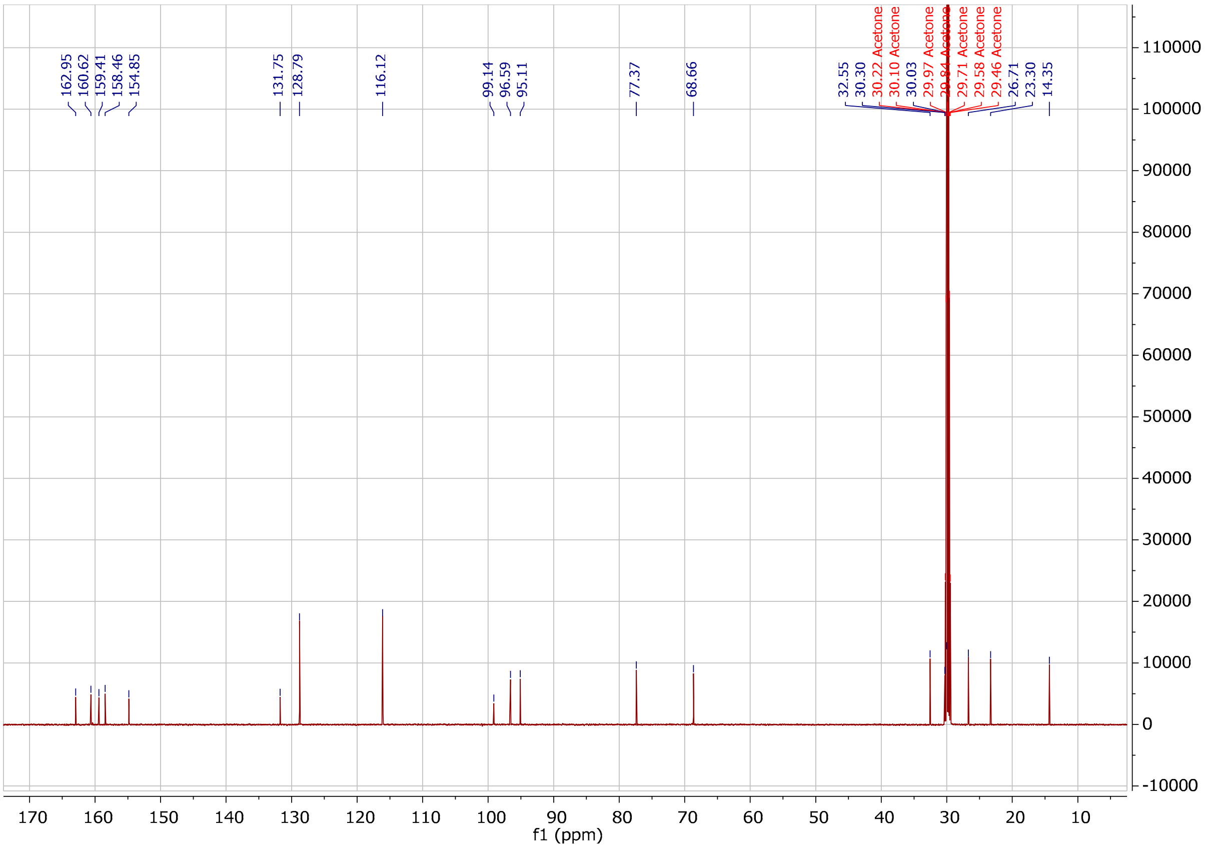

Figure S42. ^{13}C NMR (150 MHz, acetone-d_6) spectrum of 7-*O*-octylnaringenin oxime (**B5**)

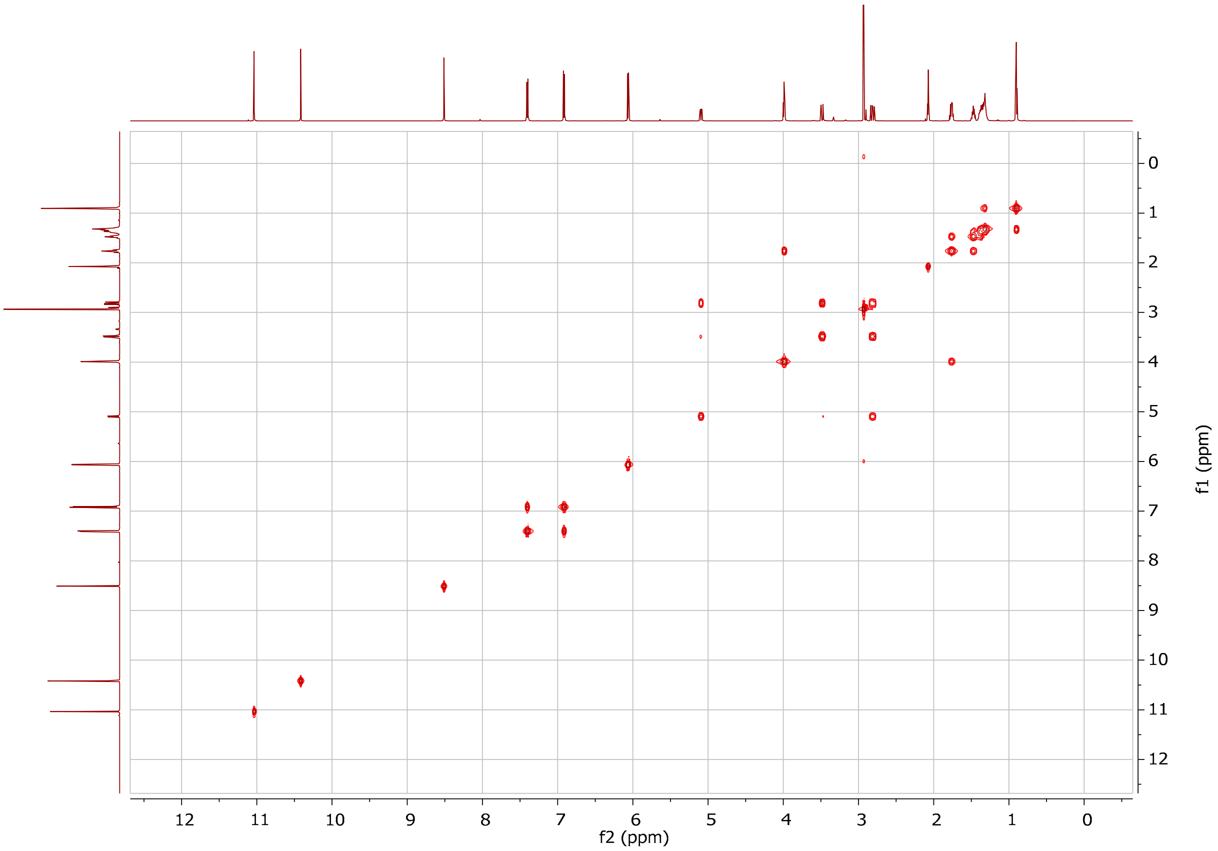

Figure S43. COSY NMR (150 MHz, acetone-d_6) spectrum of 7-*O*-octylnaringenin oxime (**B5**)

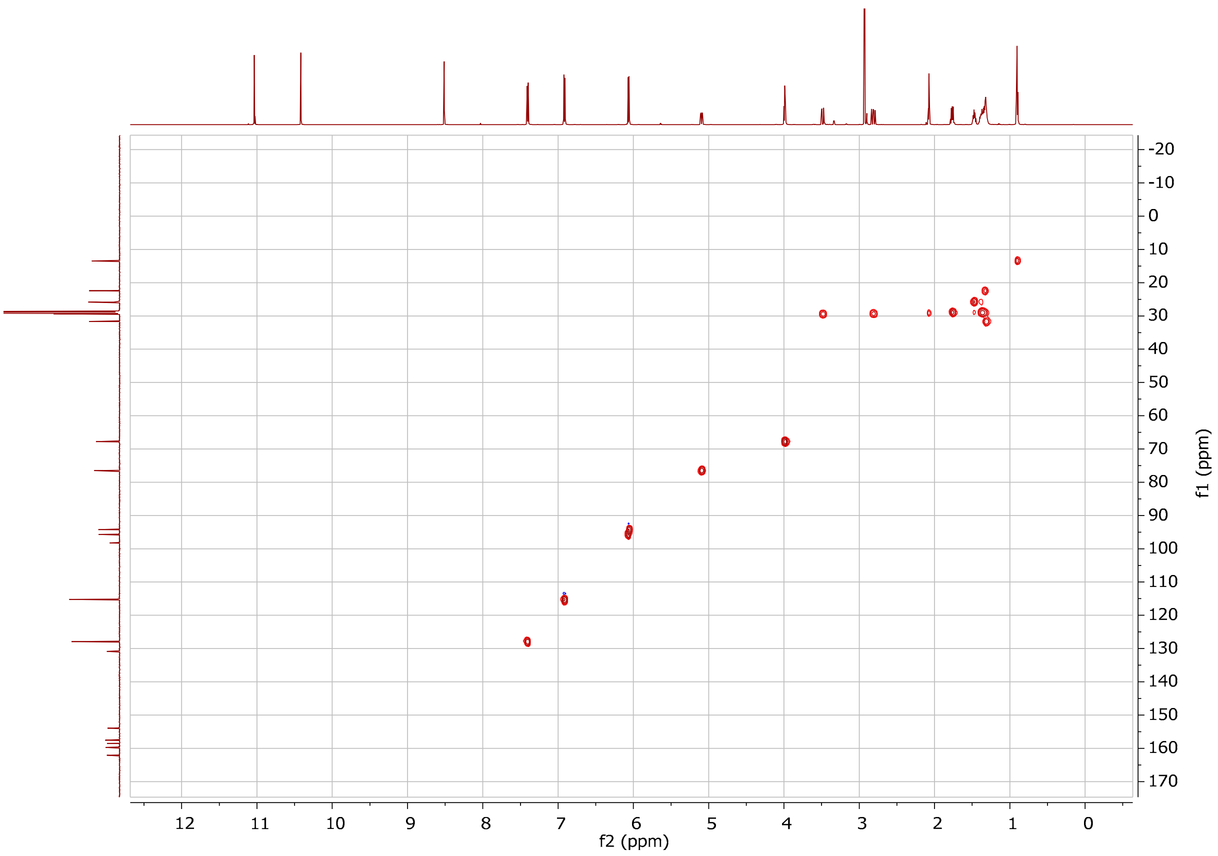

Figure S44. HSQC NMR (150 MHz, acetone-d_6) spectrum of 7-*O*-octylnaringenin oxime (**B5**)

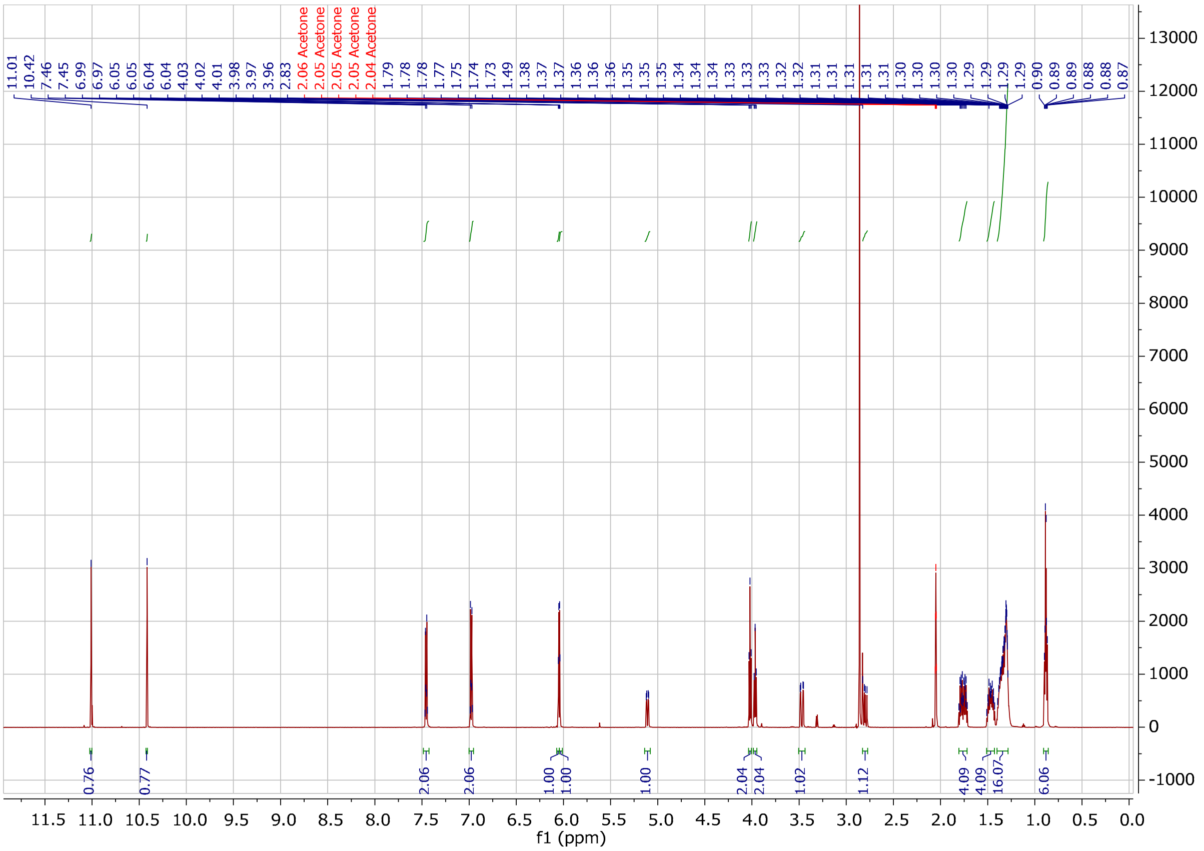

Figure S45. ^{1}H NMR (600 MHz, acetone-d_6) spectrum of 7,4′-di-*O*-octylnaringenin oxime (**B6**)

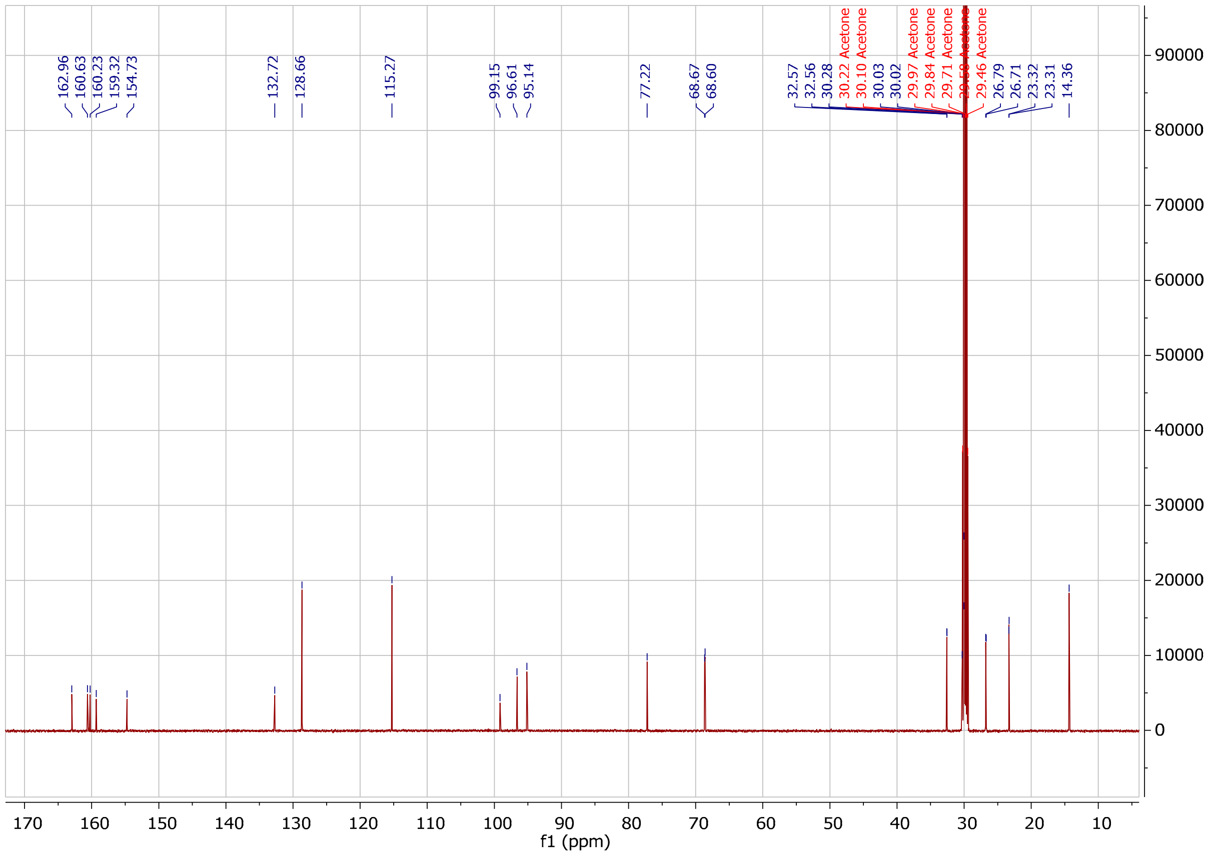

Figure S46. ^{13}C NMR (150 MHz, acetone-d_6) spectrum of 7,4′-di-*O*-octylnaringenin oxime (**B6**)

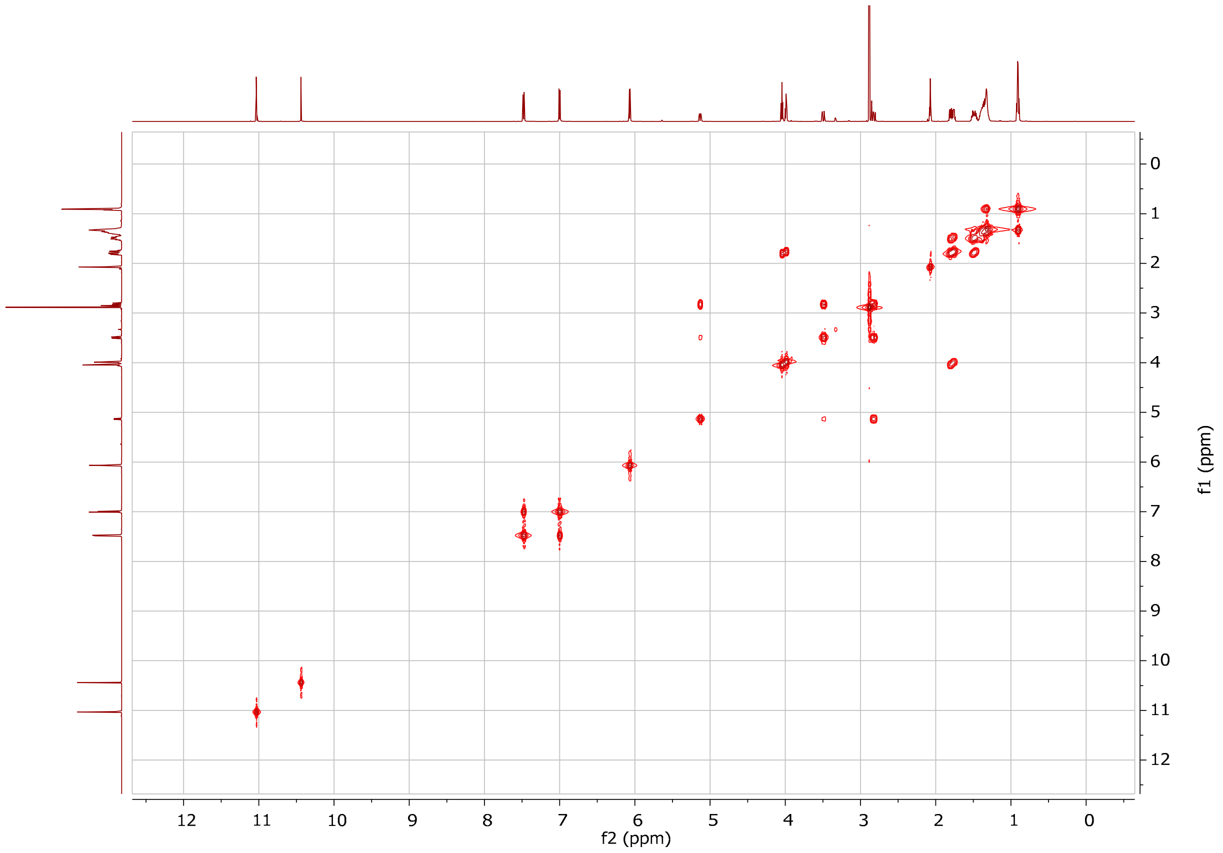

Figure S47. COSY NMR (150 MHz, acetone-d_6) spectrum of 7,4′-di-*O*-octylnaringenin oxime (**B6**)

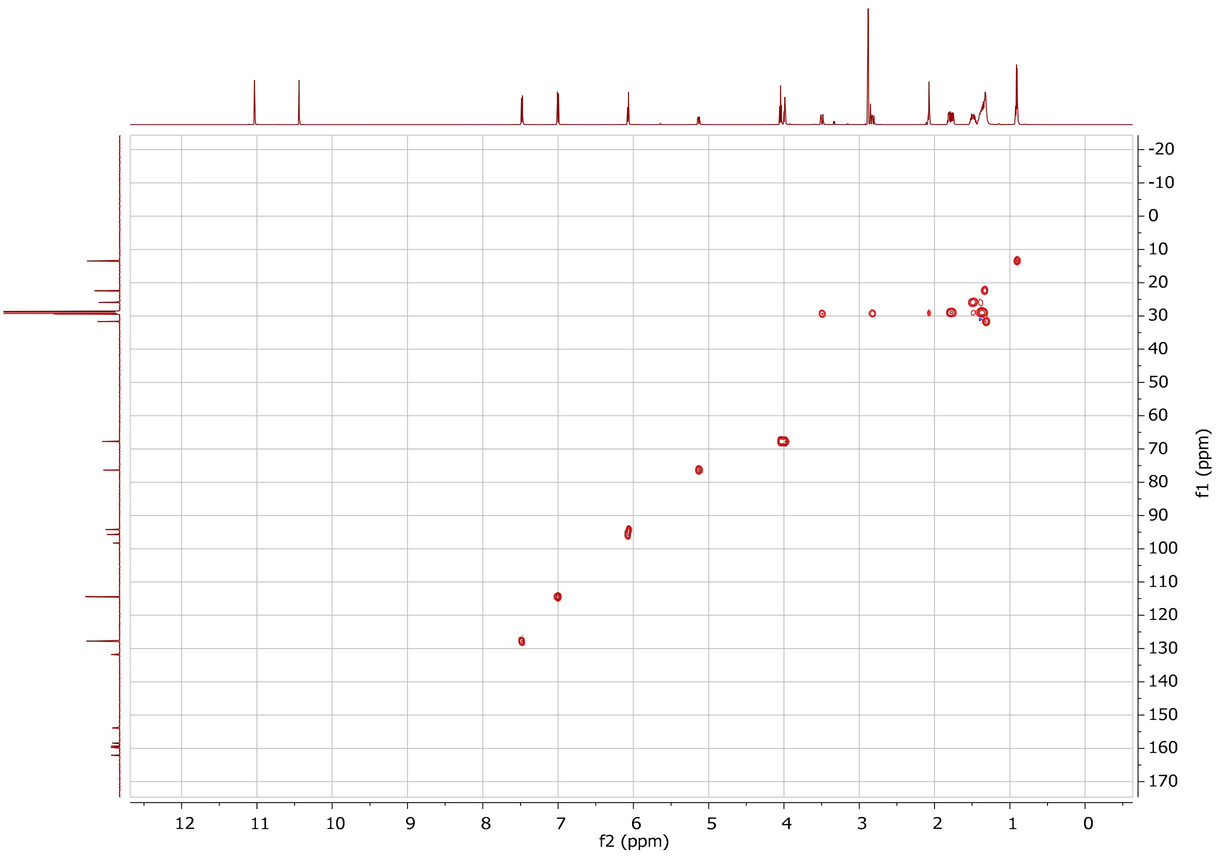

Figure S48. HSQC NMR (150 MHz, acetone-d_6) spectrum of 7,4′-di-*O*-octylnaringenin oxime (**B6**)

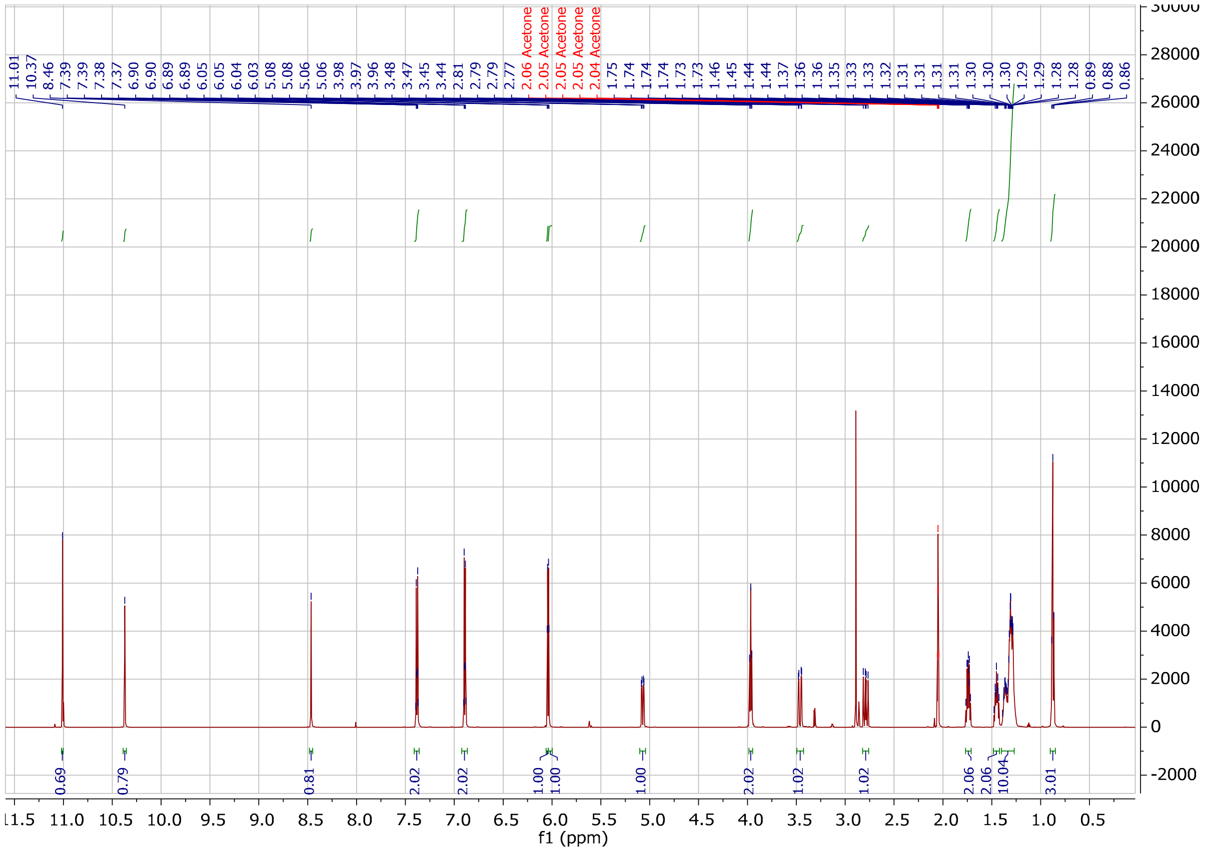

Figure S49. ^{1}H NMR (600 MHz, acetone-d_6) spectrum of 7-*O*-nonylnaringenin oxime (**B7**)

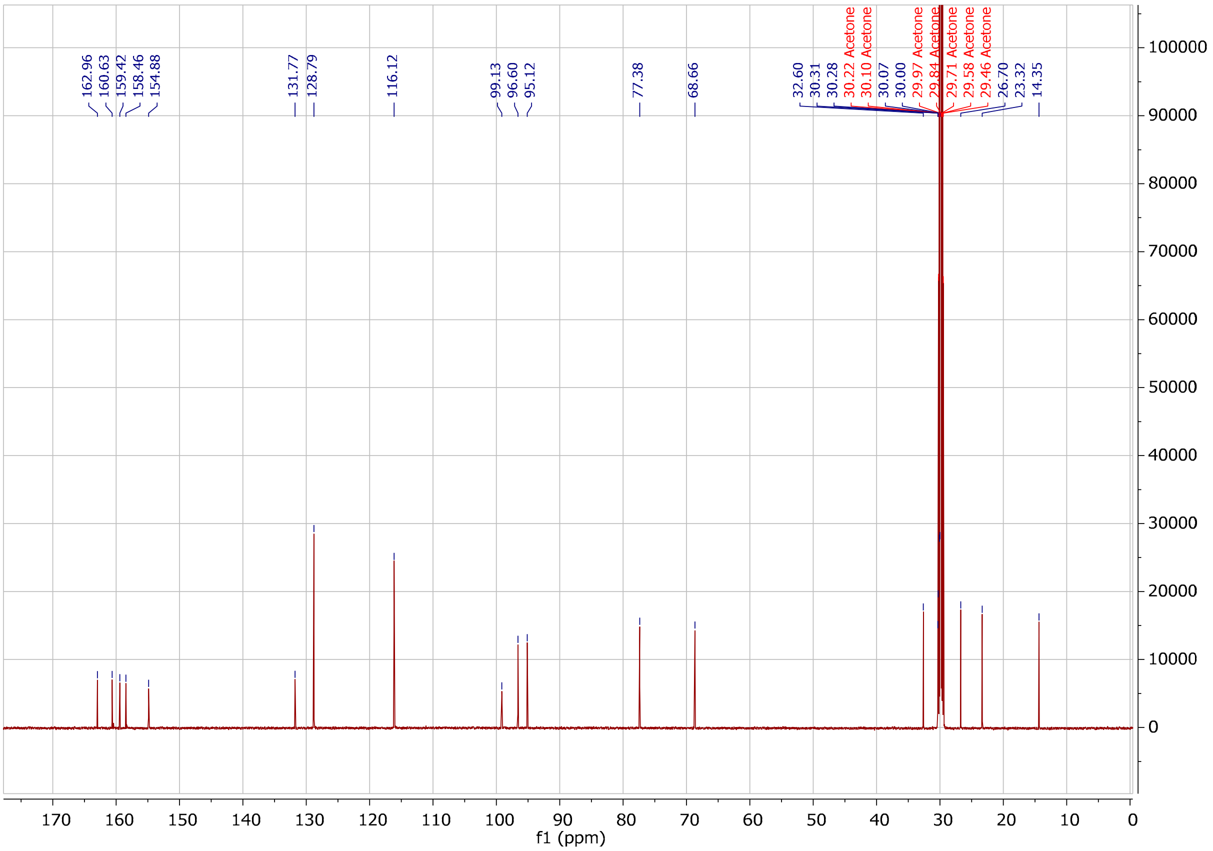

Figure S50. ^{13}C NMR (150 MHz, acetone-d_6) spectrum of 7-*O*-nonylnaringenin oxime (**B7**)

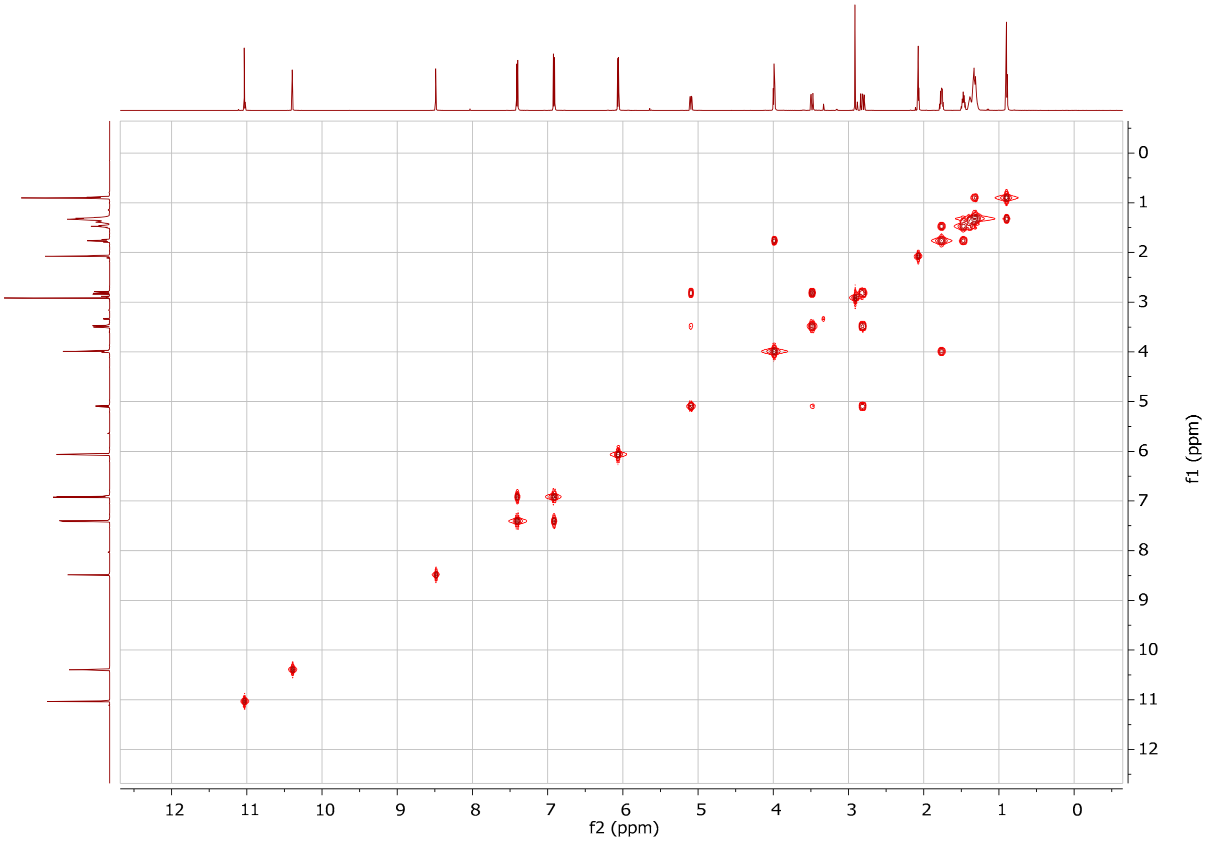

Figure S51. COSY NMR (150 MHz, acetone-d_6) spectrum of 7-*O*-nonylnaringenin oxime (**B7**)

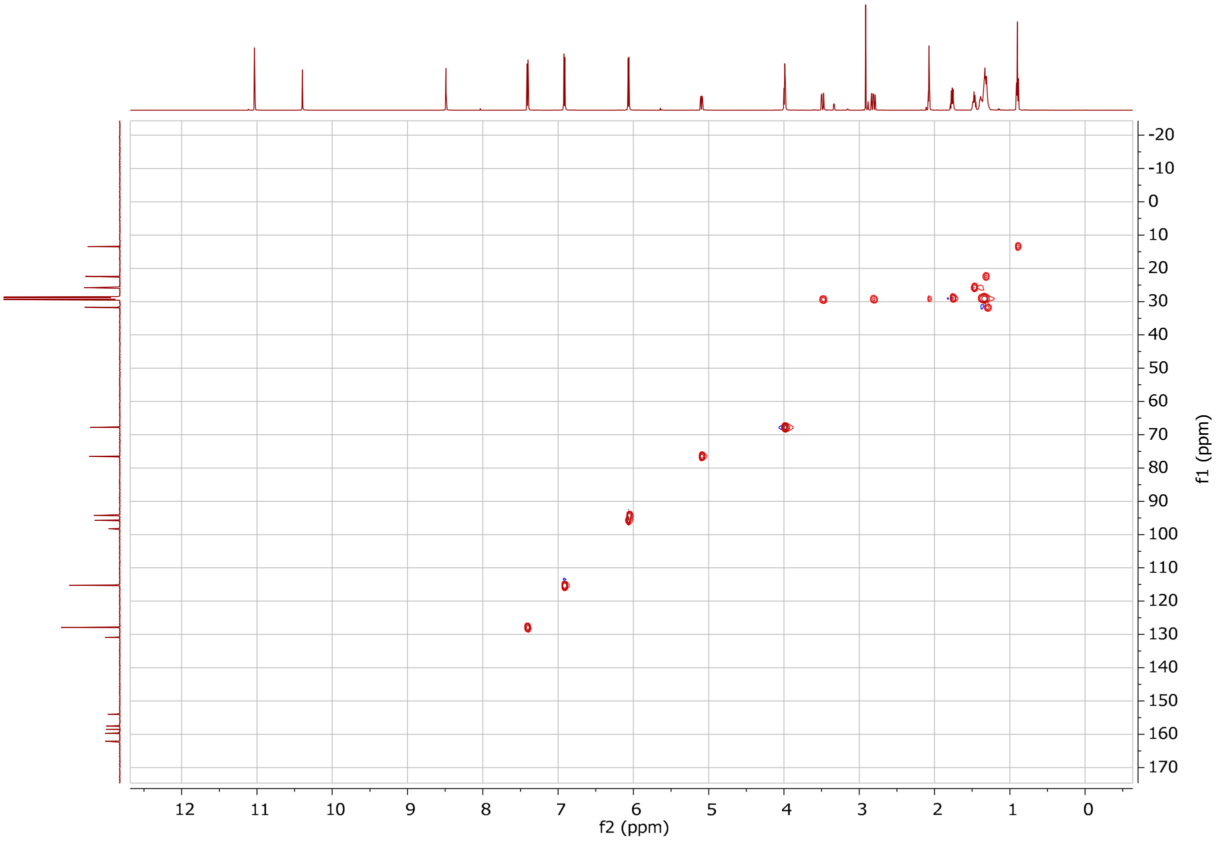

Figure S52. HSQC NMR (150 MHz, acetone-d_6) spectrum of 7-*O*-nonylnaringenin oxime (**B7**)

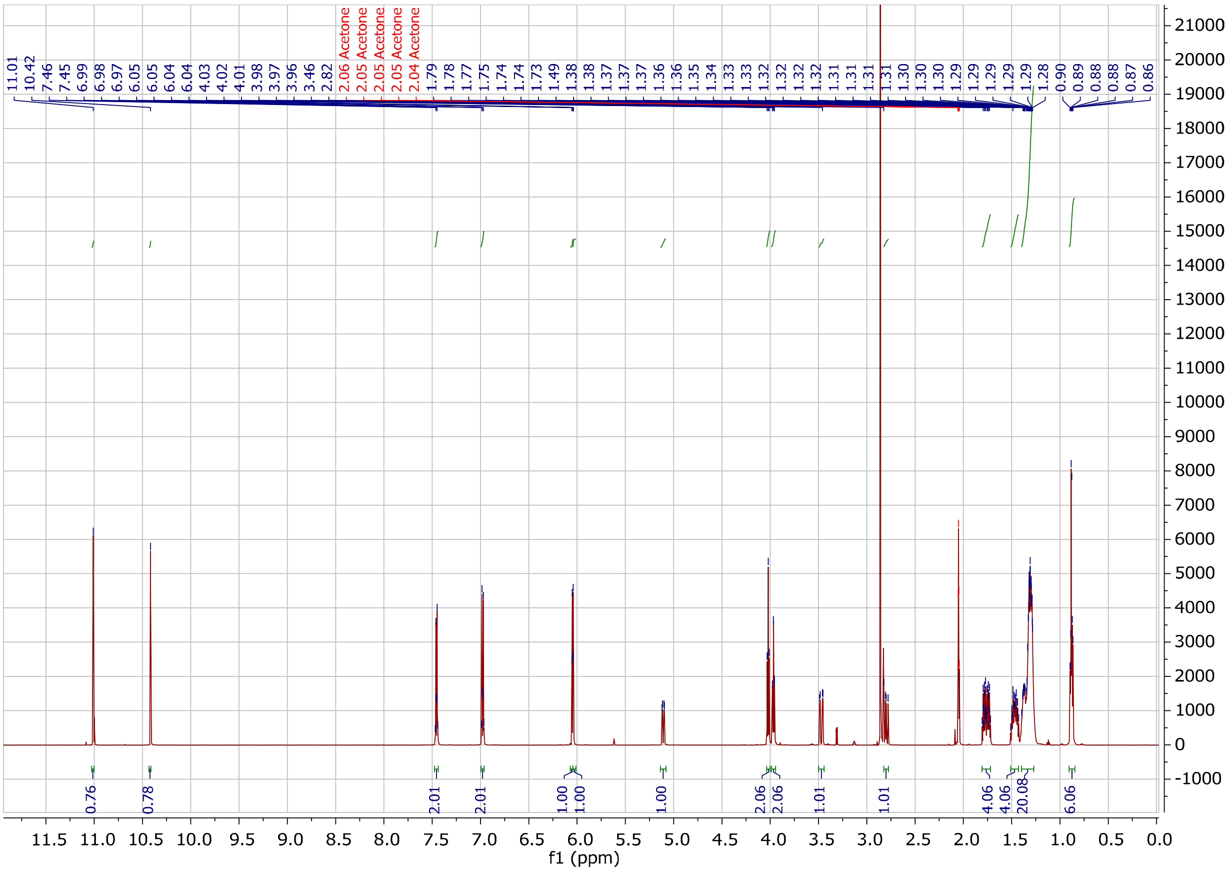

Figure S53. ^{1}H NMR (600 MHz, acetone-d_6) spectrum of 7,4′-di-*O*-nonylnaringenin oxime (**B8**)

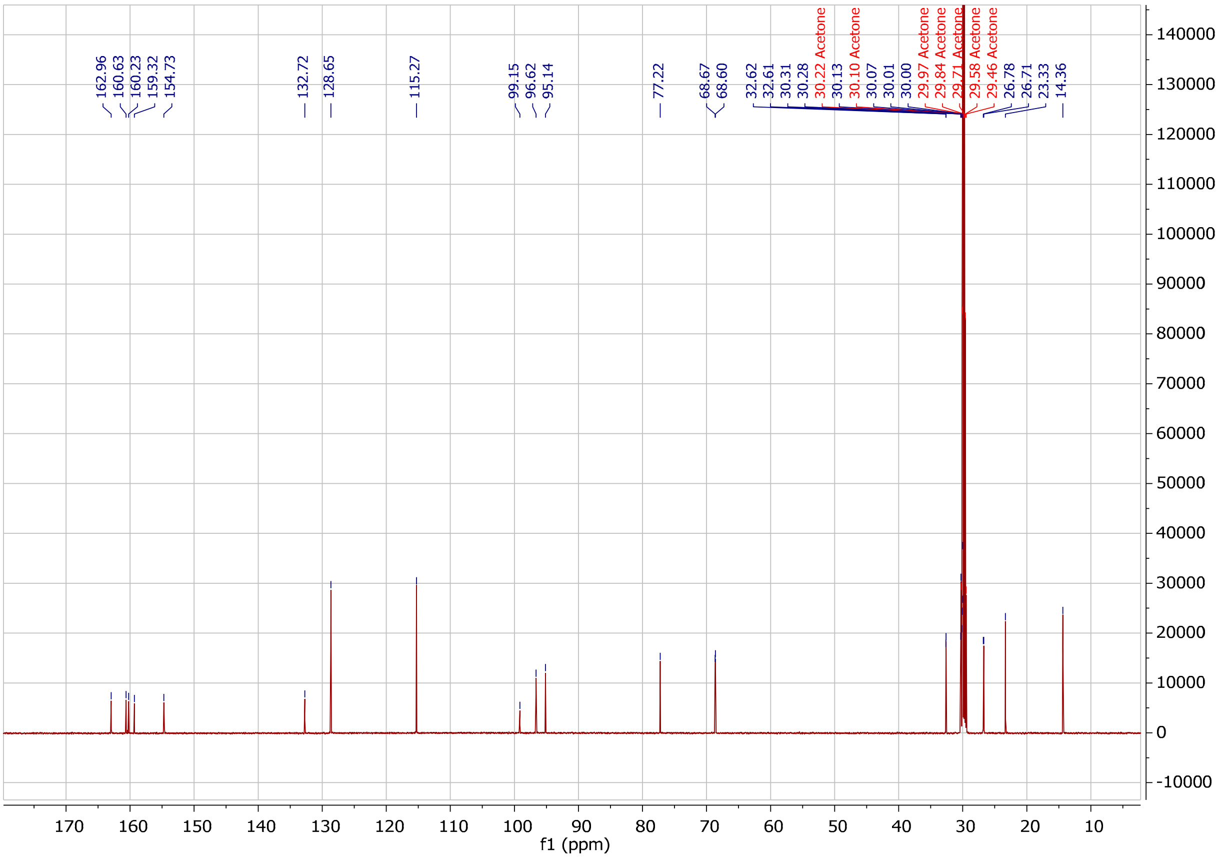

Figure S54. ^{13}C NMR (150 MHz, acetone-*d*$_6$) spectrum of 7,4′-di-*O*-nonylnaringenin oxime (**B8**)

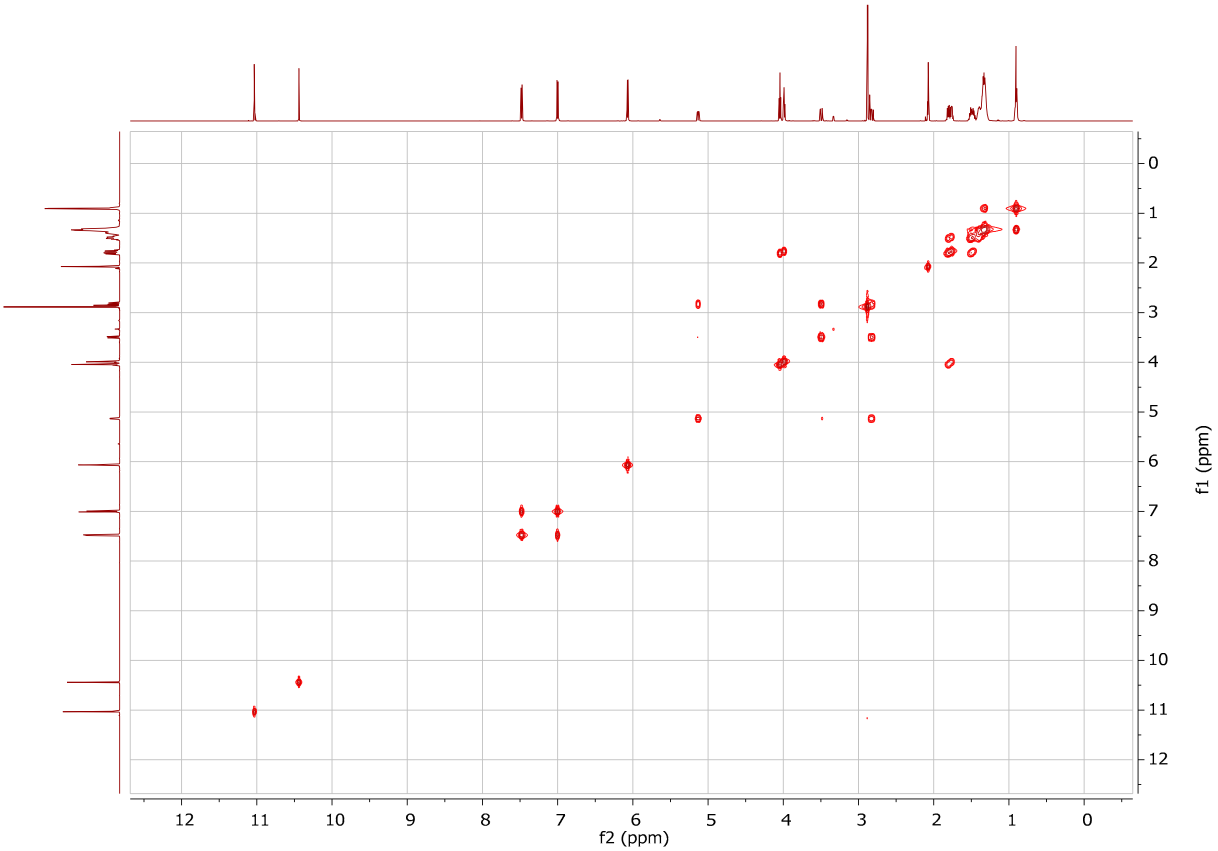

Figure S55. COSY NMR (150 MHz, acetone-*d*$_6$) spectrum of 7,4′-di-*O*-nonylnaringenin oxime (**B8**)

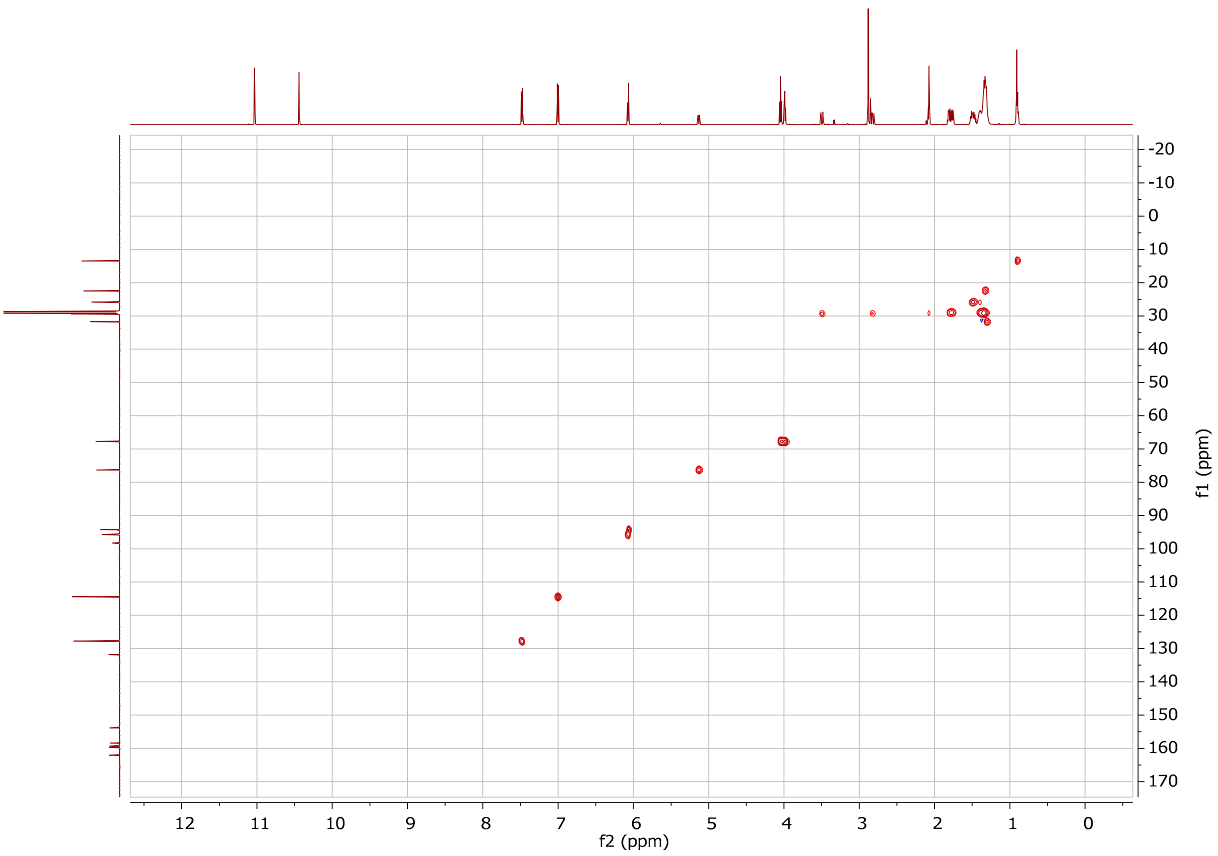

Figure S56. HSQC NMR (150 MHz, acetone-d_6) spectrum of 7,4′-di-*O*-nonylnaringenin oxime (**B8**)

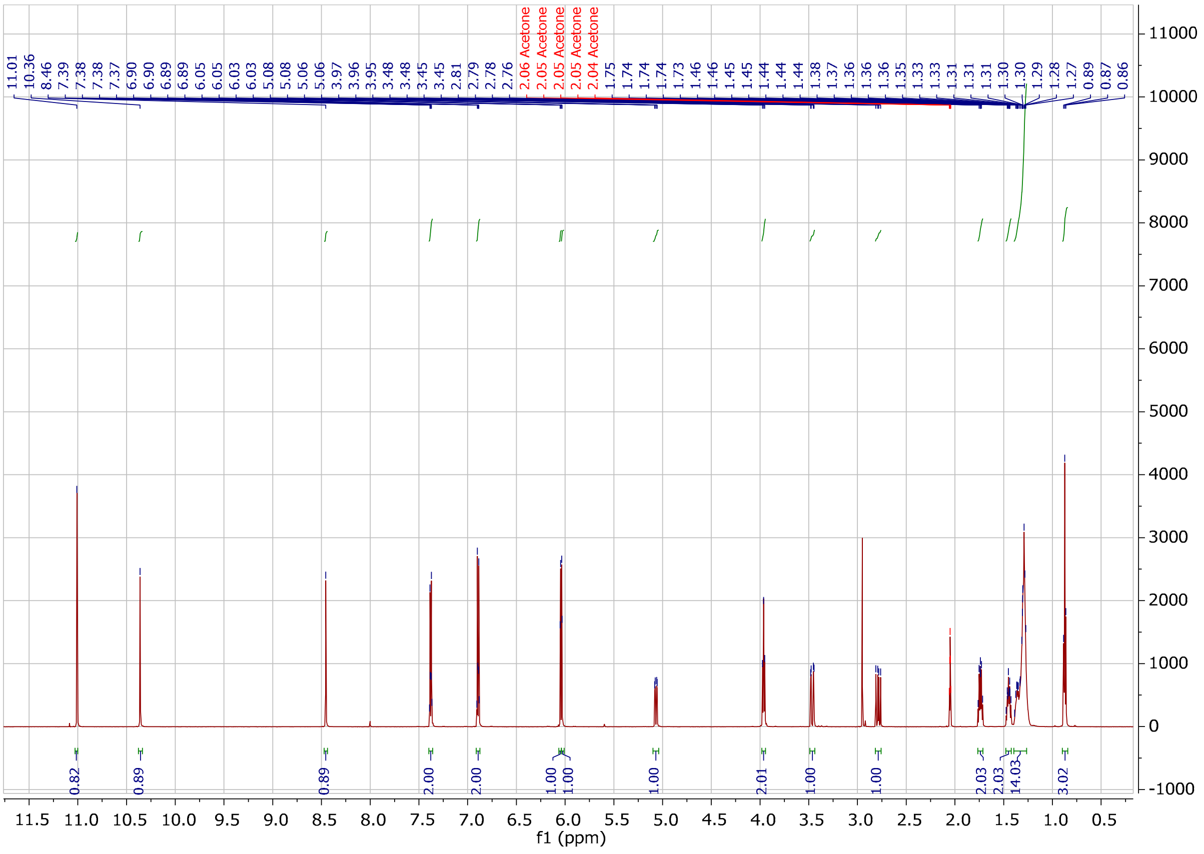

Figure S57. ^{1}H NMR (600 MHz, acetone-d_6) spectrum of 7-*O*-undecylnaringenin oxime (**B9**)

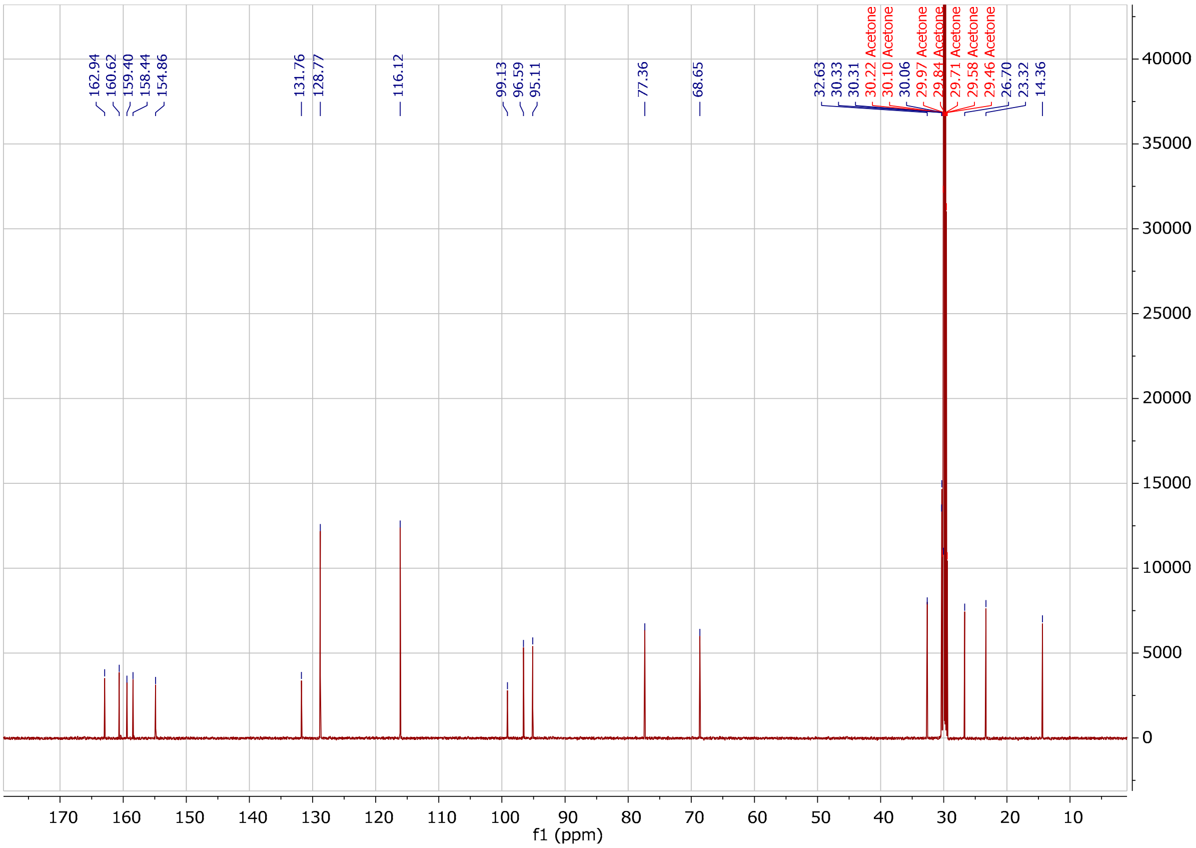

Figure S58. ^{13}C NMR (150 MHz, acetone-d_6) spectrum of 7-*O*-undecylnaringenin oxime (**B9**)

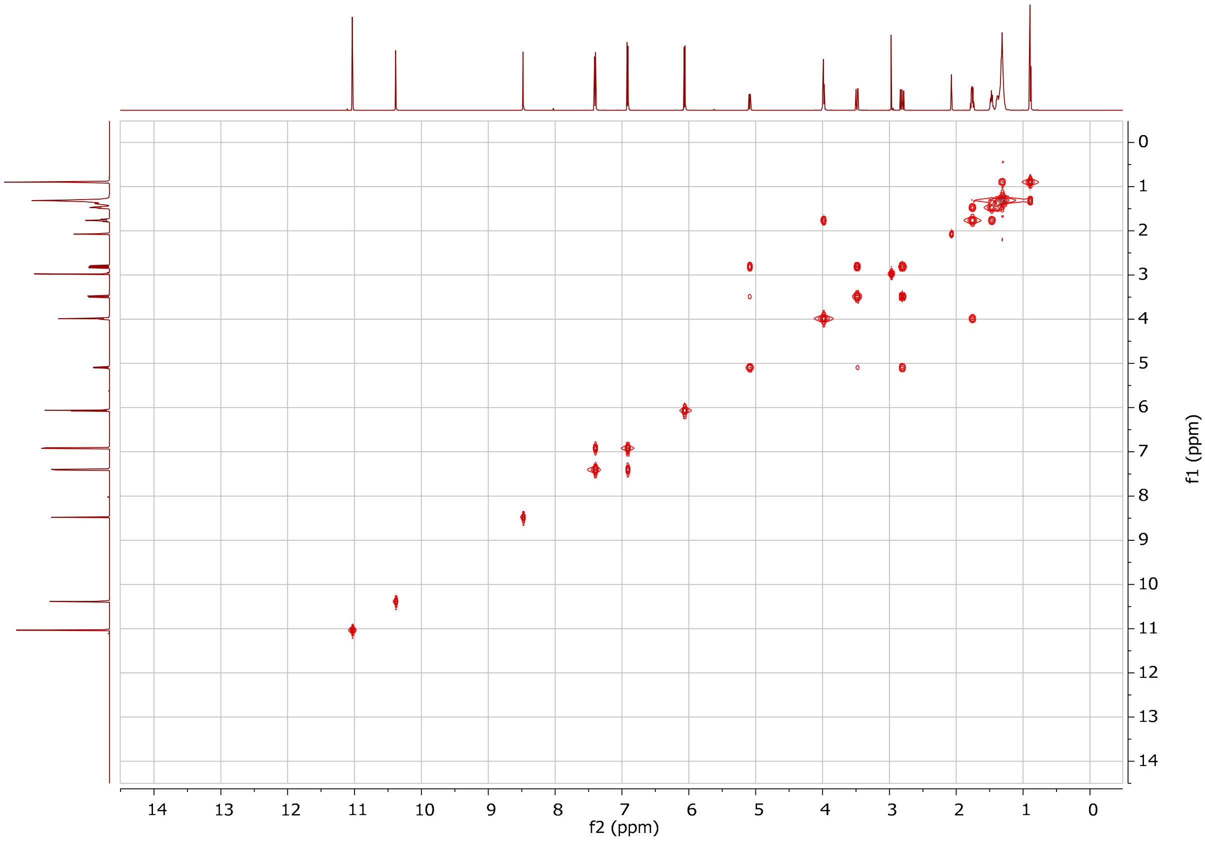

Figure S59. COSY NMR (150 MHz, acetone-d_6) spectrum of 7-*O*-undecylnaringenin oxime (**B9**)

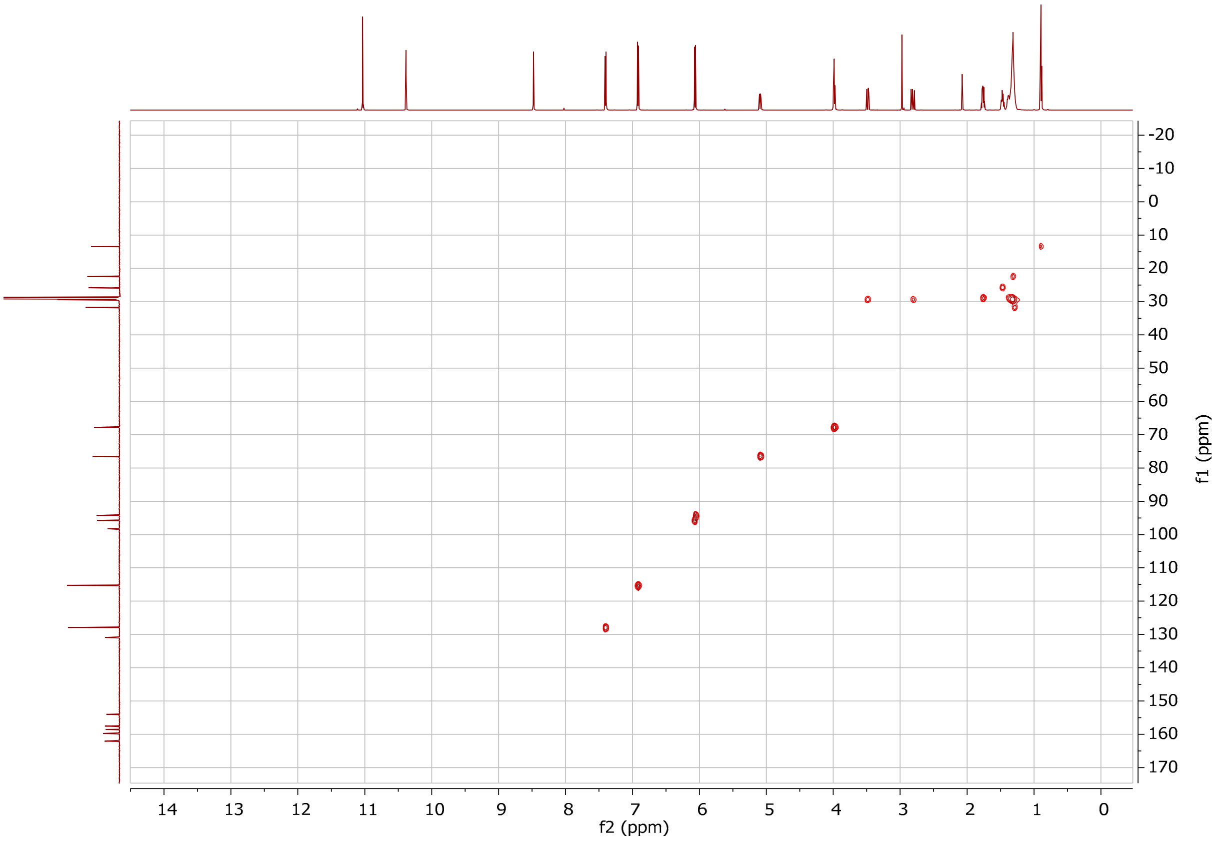

Figure S60. HSQC NMR (150 MHz, acetone-d_6) spectrum of 7-*O*-undecylnaringenin oxime (**B9**)

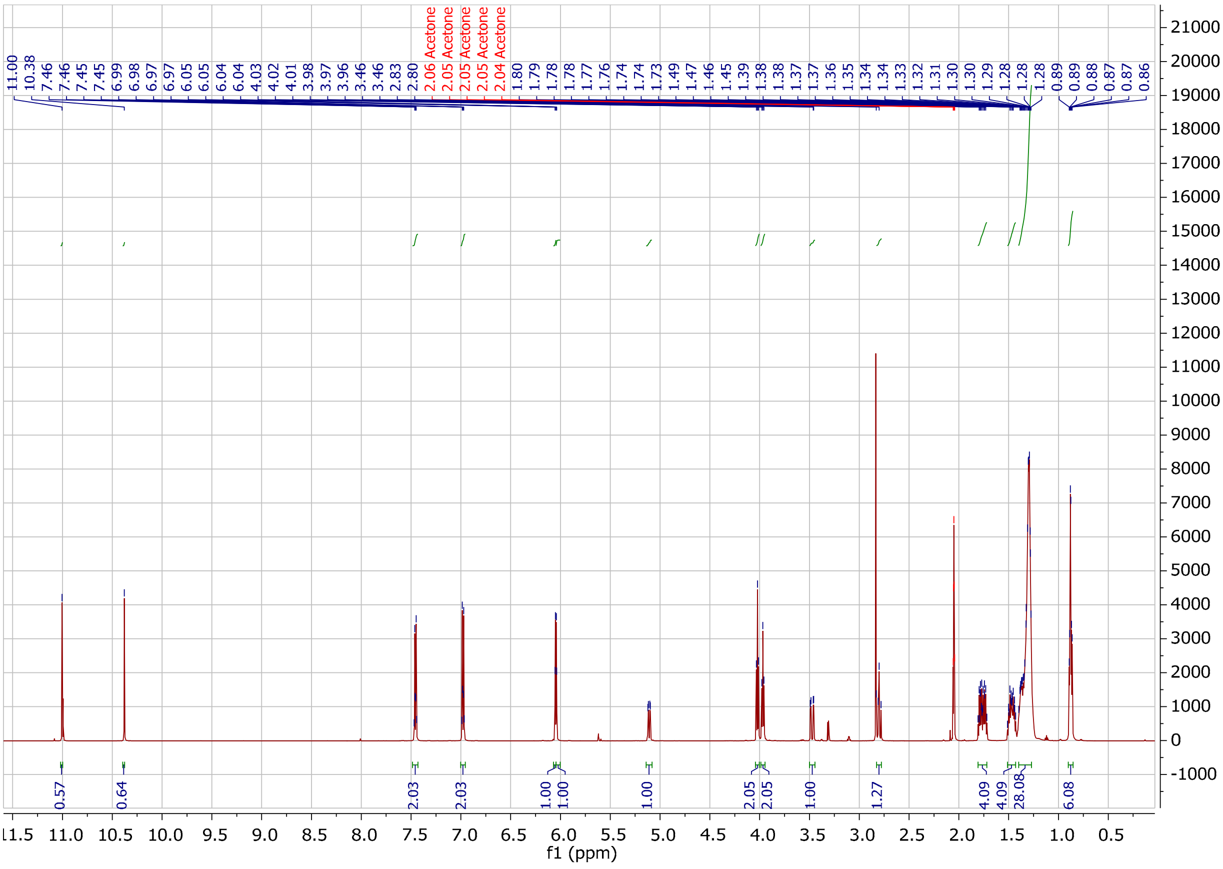

Figure S61. ^{1}H NMR (600 MHz, acetone-d_6) spectrum of 7,4′-di-*O*-undecylnaringenin oxime (**B10**)

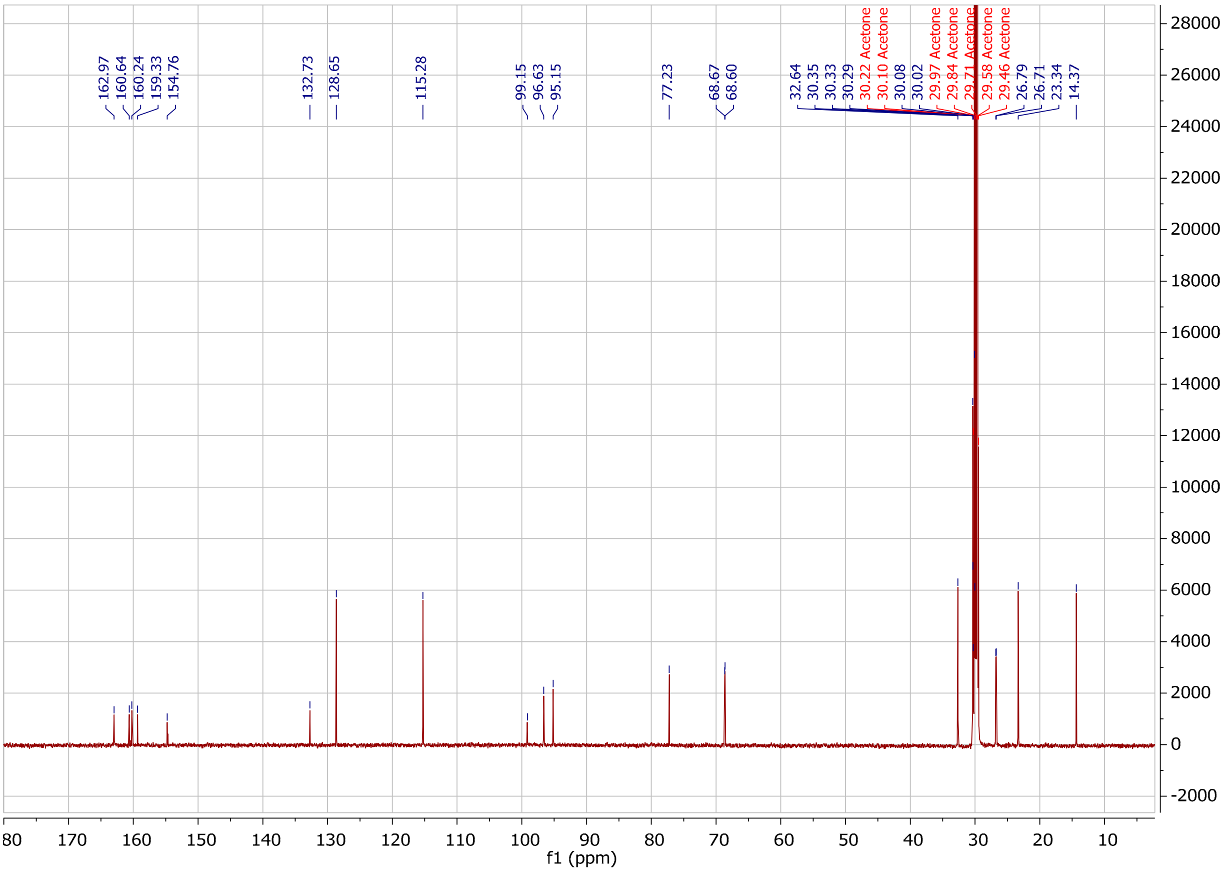

Figure S62. ^{13}C NMR (150 MHz, acetone-d_6) spectrum of 7,4′-di-*O*-undecylnaringenin oxime (**B10**)

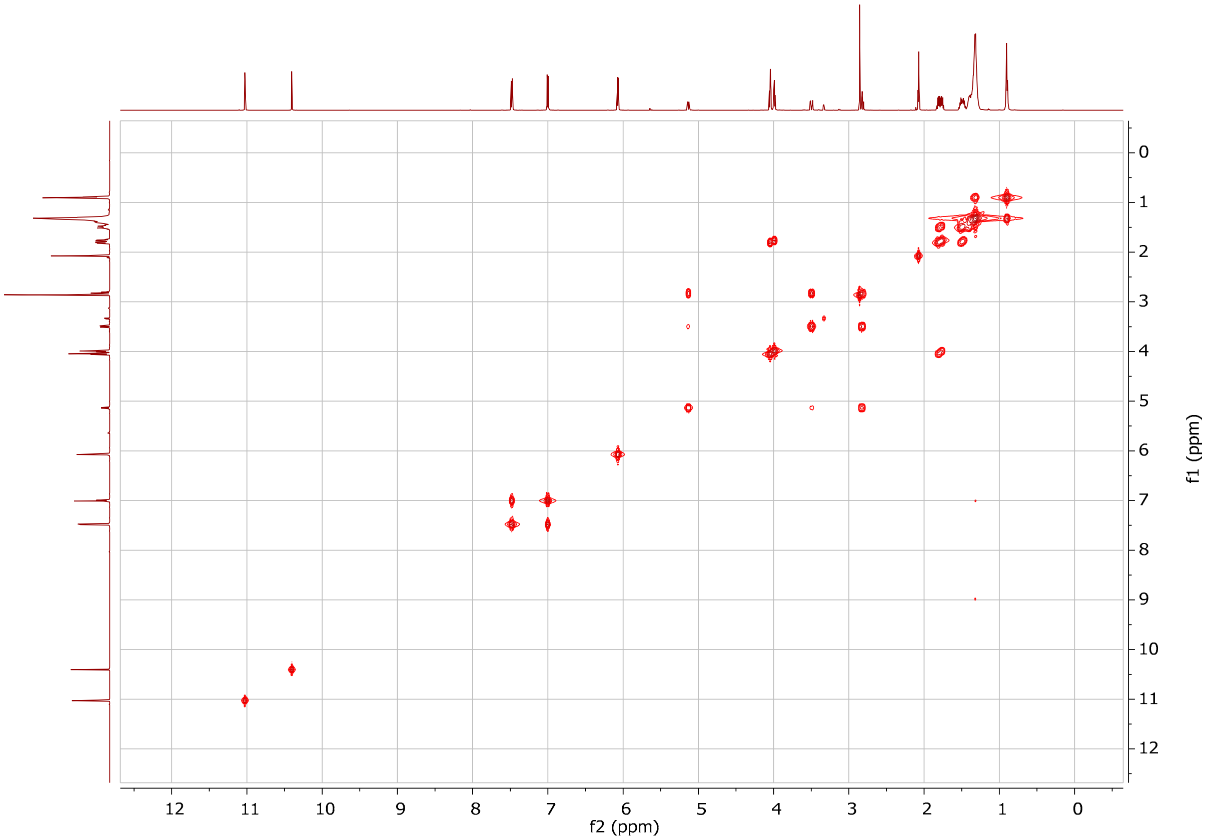

Figure S63. COSY NMR (150 MHz, acetone-d_6) spectrum of 7,4′-di-*O*-undecylnaringenin oxime (**B10**)

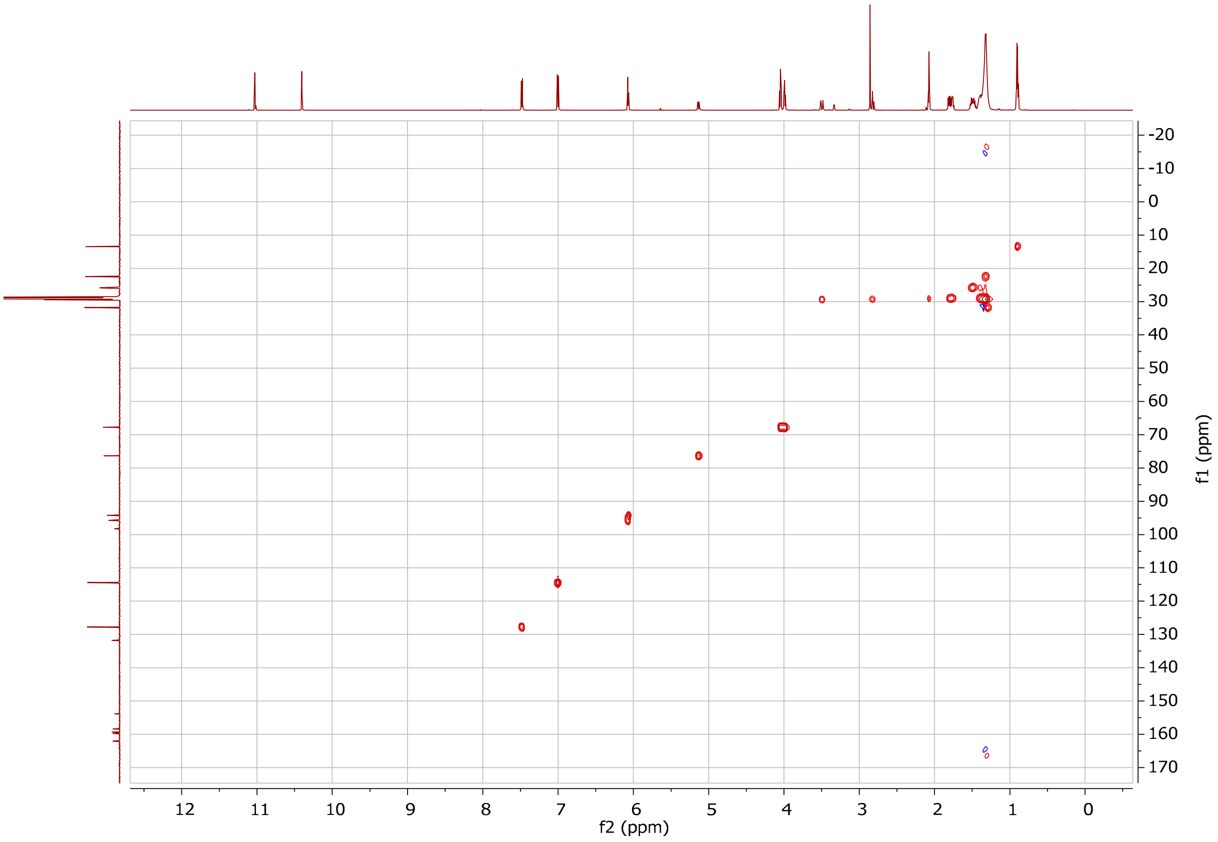

Figure S64. HSQC NMR (150 MHz, acetone-d_6) spectrum of 7,4′-di-*O*-undecylnaringenin oxime (**B10**)
